# *In extracto* cryo-EM reveals eEF2 as a major hibernation factor on 60S and 80S particles

**DOI:** 10.7554/eLife.110114

**Published:** 2026-09-03

**Authors:** Zahra Seraj, Ximena Zottig, ChunYing Huang, Anna B Loveland, Stephen Diggs, Emily Sholi, Nikolaus Grigorieff, Andrei A Korostelev

**Affiliations:** 1 RNA Therapeutics Institute, UMass Chan Medical School Worcester United States; 2 Howard Hughes Medical Institute, UMass Chan Medical School Worcester United States; Stanford University School of Medicine, Howard Hughes Medical Institute United States; Stanford University United States

**Keywords:** human, *H. sapiens*, mammalian, rabbit, *Oryctolagus cuniculus*, RRL, Other

## Abstract

Cryogenic electron microscopy (cryo-EM) made impressive progress in resolving cellular macromolecules and their detailed interactions. Single-particle cryo-EM traditionally relies on purified macromolecules and lacks the complexity of cellular environments, whereas *in situ* cryo-EM and cryogenic electron tomography (cryo-ET) require extensive sample preparation and data acquisition, presenting challenges in achieving high resolution. We describe cryo-EM of cellular lysates—*in extracto* cryo-EM—allowing the flexibility and high-resolution of cryo-EM in the context of cellular components. High-resolution 2D template matching (2DTM) yields ~2.2 Å maps of the mammalian translational apparatus. Elongating ribosome abundances in primate cell lines (MCF-7 and BSC-1) and rabbit reticulocyte lysates range from ~70% to ~10%, reflecting translational stress responses. Non-translating (hibernating) ribosomes carrying no mRNA feature numerous proteins shielding ribosomal functional centers. Elongation factor 2 (eEF2) is the most abundant hibernation factor bound to >95% of 80S ribosomes and, unexpectedly, to 60S subunits. eEF2•GDP is stabilized by interactions with the sarcin-ricin loop and protein uL14. Hibernating ribosomes also feature La-related protein 1 (LARP1) involved in initiation and mTOR signaling, eIF5A implicated in elongation and termination, and other factors, exposing the variety of hibernation scenarios. Our work underscores the efficiency and potential of *in extracto* cryo-EM to discover native cellular complexes and mechanisms at near-atomic resolution.

## Introduction

The renaissance of structural biology, thanks to high-resolution cryogenic electron microscopy (cryo-EM) (Bai et al., 2013; Liao et al., 2013; Campbell et al., 2012), is rapidly expanding our understanding of biological processes, from visualizing dynamic macromolecules to discovering new cellular compartments (Heydari and Liu, 2025; Chua et al., 2022; Young and Villa, 2023; Carter et al., 2020). Transmission electron microscopy is employed to study biological samples in two major applications: single-particle (*in vitro*) cryo-EM of purified macromolecules and reconstituted complexes, and cellular/tissue (*in situ*) cryo-EM, the most notable approach being cryogenic electron tomography (cryo-ET). Each approach has advantages and limitations. Single-particle cryo-EM allows elucidation of structural details of interactions and reactions. Here, analyses of large datasets (hundreds of thousands to >1 million particles) separate conformationally and compositionally distinct states (‘classes’), yielding near-atomic resolution (~2–3.5 Å) insights into macromolecular dynamics and interactions. Furthermore, datasets can be collected at different time points of a reaction, allowing time-resolved cryo-EM to uncover transient intermediates in the absence of inhibitors (Klebl et al., 2025; Banari et al., 2025; Korostelev, 2022). For example, recent studies revealed the dynamics of the ribosome and translation factors during translation initiation (Kaledhonkar et al., 2019), elongation (Carbone et al., 2021; Loveland et al., 2020), termination (Fu et al., 2019; Dadhwal, 2024), and recycling (Fu et al., 2016; Bhattacharjee et al., 2024). However, because samples are usually assembled from a limited set of purified components, this approach does not account for yet-to-be-discovered cellular interactions that may be important for the processes under investigation. To fill that gap, electron microscopy can be performed on cellular samples. Cryo-ET is one of the most popular approaches, providing impressive three-dimensional (3D) views of cellular compartments and large macromolecular complexes *in situ* (Nogales and Mahamid, 2024; de Teresa-Trueba et al., 2023; Zhang et al., 2022). Because the cellular environment is densely filled with macromolecules, electron microscopy of cellular samples remains a challenge and requires laborious sample preparation, data collection, and processing (Nogales and Mahamid, 2024). For example, focused ion beam (FIB) milling of frozen cells to generate the number of thin areas (lamellae) necessary to collect large datasets can take days if not weeks. Furthermore, sub-tomogram averaging of specific macromolecular densities usually does not achieve the near-atomic resolution of similar macromolecules analyzed by single-particle cryo-EM. Finally, it remains challenging to manipulate cells to study a particular aspect of a cellular process, such as a specific step of a multi-step pathway, without using modulators/inhibitors that may bias cells off pathway.

In this work, we explore the advantages of both methods by exploiting cellular extracts to elucidate novel cellular complexes and interactions at high resolution. We demonstrate that high-resolution 2D template matching (2DTM) (Lucas et al., 2021) enables the reconstructions of mammalian 80S complexes at ~2.2 Å resolution, similar to single-particle cryo-EM from purified ribosomes. We studied 80S translation complexes in several mammalian lysates, starting with the optimization of the cryo-EM/2DTM pipeline on lysates obtained from a human breast cancer cell line MCF-7 and a monkey kidney cell line BSC-1. Our aim was to compare the translational states of ribosomes in distinct cell types and to visualize how cellular stress affects these states in MCF-7. We find that MCF-7 and BSC-1 lysates feature 60–70% of elongation-like complexes, echoing the analyses of mammalian translation in cells (Gemmer et al., 2023; Zheng et al., 2025). In nutrient-deprived MCF-7, the abundance of elongation complexes is reduced by ~15%, consistent with inhibition of translation upon eIF2α phosphorylation (Harding et al., 2000; Donnelly et al., 2013).

We also characterized the widely used rabbit reticulocyte lysates (RRLs), the predominant model system to study translation since the 1970s (Pelham and Jackson, 1976). RRL is a flexible and tunable system that allows monitoring of translation of a single mRNA (e.g. a reporter mRNA encoding a luciferase). Studies in RRL have led to discoveries or mechanistic descriptions of translation factors (Pestova and Kolupaeva, 2002), internal ribosome entry sites (Quade et al., 2015; Yamamoto et al., 2015), and other translational regulators (Rifo et al., 2007; Thoms et al., 2020). Yet, the composition of translation complexes in RRL remains uncharacterized. Unlike purified ribosome complexes, we find more multi-part compositions of 80S complexes, bringing new insights into translation regulation. For example, we find that after a few minutes of translation in RRL, only a small fraction of ribosomes is involved in translating a reporter mRNA (10–12%), whereas the majority of 80S complexes are in ‘hibernating’ complexes bound with eEF2 but lacking mRNA. In addition, these complexes contain eIF5A, primarily implicated in translation elongation and termination (Schuller et al., 2017), La-related protein 1 (LARP1) implicated in regulation of terminal oligopyrimidine (TOP)-containing mRNAs (Saba et al., 2024; Wolin et al., 2025), and previously identified hibernation factors SERBP1, CCDC124, and IFRD2 (Du et al., 2024; Wells et al., 2020; Brown et al., 2018). These findings reveal that all functional centers of the ribosome are shielded in 80S hibernating complexes.

Simple lysate preparation and straightforward data processing make ‘*in extracto* cryo-EM’ an efficient approach, allowing visualization of cellular complexes at near-atomic resolution. Furthermore, some parameters, such as substrate (e.g. mRNA) composition and concentration, can be tuned to assemble the desired complex(es) without the need to reconstitute the specific complex(es) or to perturb cells by modulators/inhibitors. Our approach therefore offers a flexible method to study translation, and potentially other processes, in the context of authentic cellular components that may affect these processes.

## Results and discussion

### 2DTM resolves translating and hibernating ribosomes in fresh cell lysates

In our recent work describing angiogenin activation by ribosomes in RRLs (Loveland et al., 2024), we investigated how the angiogenin-ribosome interaction is affected by other cellular components. Typical lysate preparations produce dense and viscous samples, making it challenging to prepare cryo-EM grids with thin sample layers and to analyze them by standard cryo-EM methods. Indeed, standard data-processing workflows using Relion and *cis*TEM (Scheres, 2012; Grant et al., 2018), which we and others routinely use to process single-particle data, failed to yield high-quality maps of distinct classes. Extensive culling of micrographs, manual particle picking, and 2D classification to remove low-quality particles was required, but this eventually resulted in small, good-quality particle stacks (~6000 or ~17,000), yielding only three distinct ribosome classes with reconstructions at ~3.2–4.4 Å average resolution. To overcome this problem, we used high-resolution 2DTM, which was recently shown to successfully identify ribosomes in dense cellular environments (Lucas et al., 2021). This method substantially improved the number of picked particles (~84,000), yielding higher-resolution maps (3 Å), and separating tens of classes from the same dataset (Methods and Supplementary file 1A).

Inspired by the success of the 2DTM approach on a previously published dataset (Loveland et al., 2024), we used this approach on freshly prepared mammalian cell lysates. We analyzed the breast-cancer-derived MCF-7 cell line under normal and nutrient-deprived conditions, testing whether the expected downregulation of the translational apparatus can be observed in lysates. We also analyzed the African Green Monkey kidney cell line BSC-1 under normal growth conditions to compare the distribution of translational complexes across distinct organisms and tissues.

**Figure 1.**
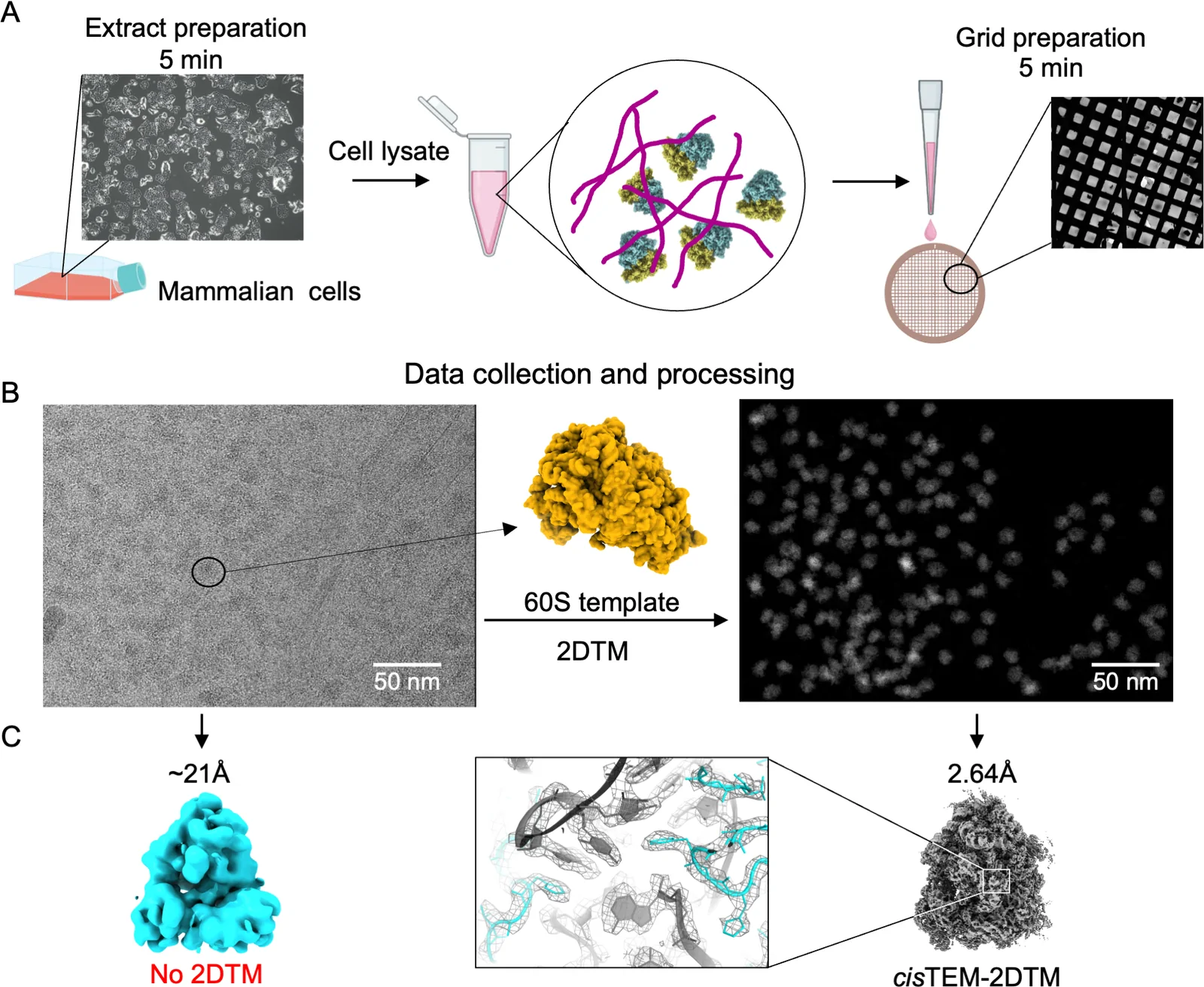
*In extracto* cryogenic electron microscopy (cryo-EM) workflow for sample preparation and data collection. (**A**) Sample and grid preparation from primate semi-permeabilized cells. Examples of an MCF-7 cell culture (left) and a grid with cell lysate (right) are shown. (**B**) Representative lysate micrograph (left) and two-dimensional template matching (2DTM) processing yielding positions and orientations of 60S subunits (right). (**C**) Examples of averaged maps (prior to classification) from typical single-particle picking pipelines (cyan) and from 2DTM processing (middle and gray). The close-up view highlights densities of 28S rRNA nucleotides (gray) and ribosomal protein residues (cyan). Also see Figure 2—figure supplement 1.

We used a mild lysis procedure (Figure 1A) relying on permeabilization of cellular membranes by the non-ionic detergent digitonin (Miyamoto et al., 2008; Weigel et al., 1983; Fan and Heerklotz, 2017), allowing preservation of translation-competent ribosome states (Behrmann et al., 2015). In the first approach, MCF-7 cells were permeabilized by a digitonin-containing lysis buffer, and the cytosol was collected from cell-containing wells and applied to cryo-EM grids. In the second approach, BSC-1 cells were detached from wells, incubated in the lysis buffer, briefly centrifuged to remove cell debris, and the supernatant was applied to cryo-EM grids. Preparation of the lysate in both cases was fast (under 10 min), and the resulting grids were directly suitable for cryo-EM data collection. In cryo-EM images, collected using standard data collection procedures employed in single-particle EM, cellular filaments can be seen next to ribosomes in the cytosol of permeabilized cells (Figure 1—figure supplement 1). Even in the lysates separated from cell debris, additional cellular components are readily visible, including mitochondria (Figure 1—figure supplement 1). Thus, a simple and streamlined approach can be used to visualize ribosomes and other cellular components.

**Figure 1—figure supplement 1.**
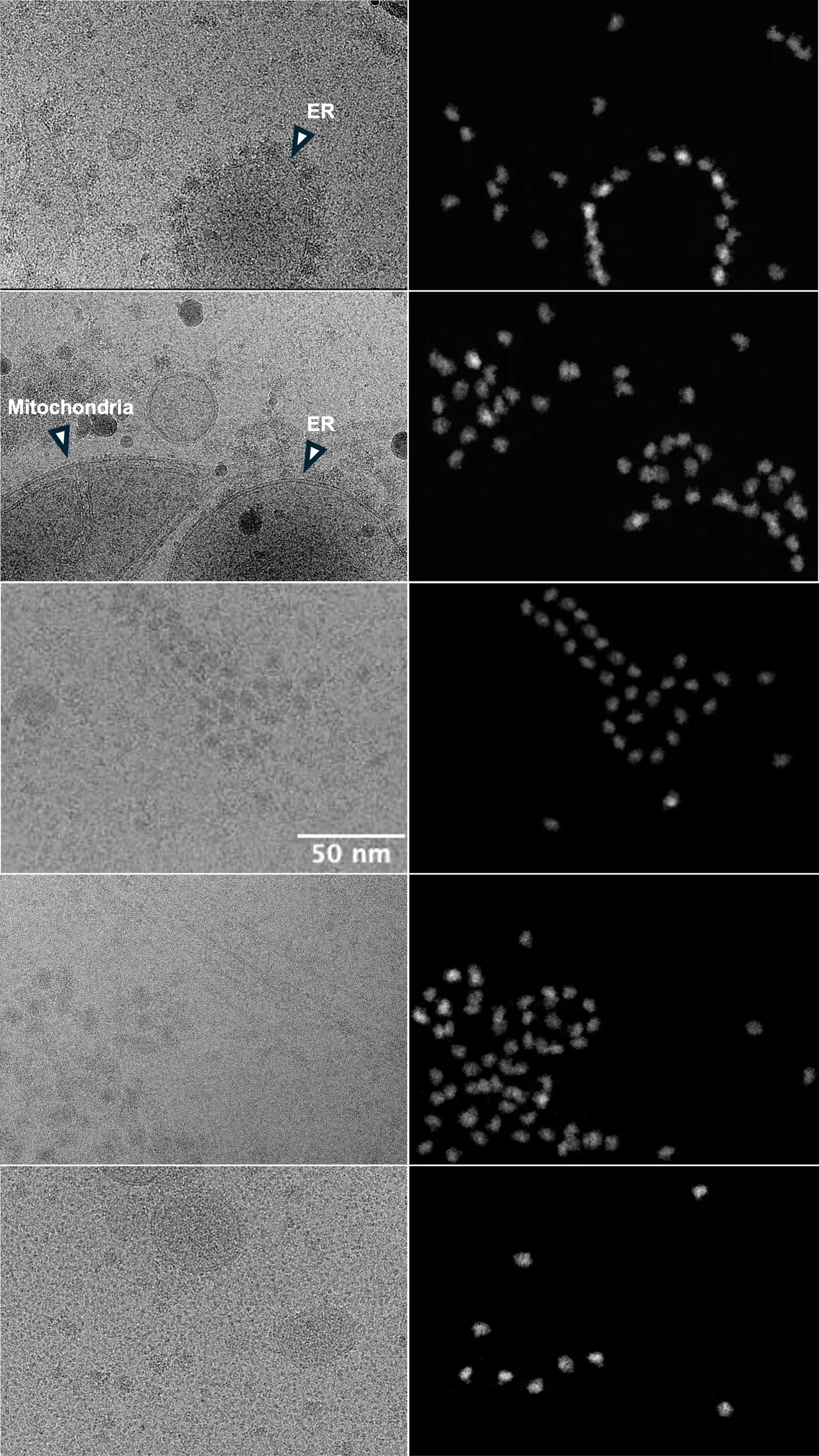
Examples of micrographs showing cellular context in BSC-1 lysates. Scale bar for all micrographs is 50 nm.

**Figure 1—figure supplement 2.**
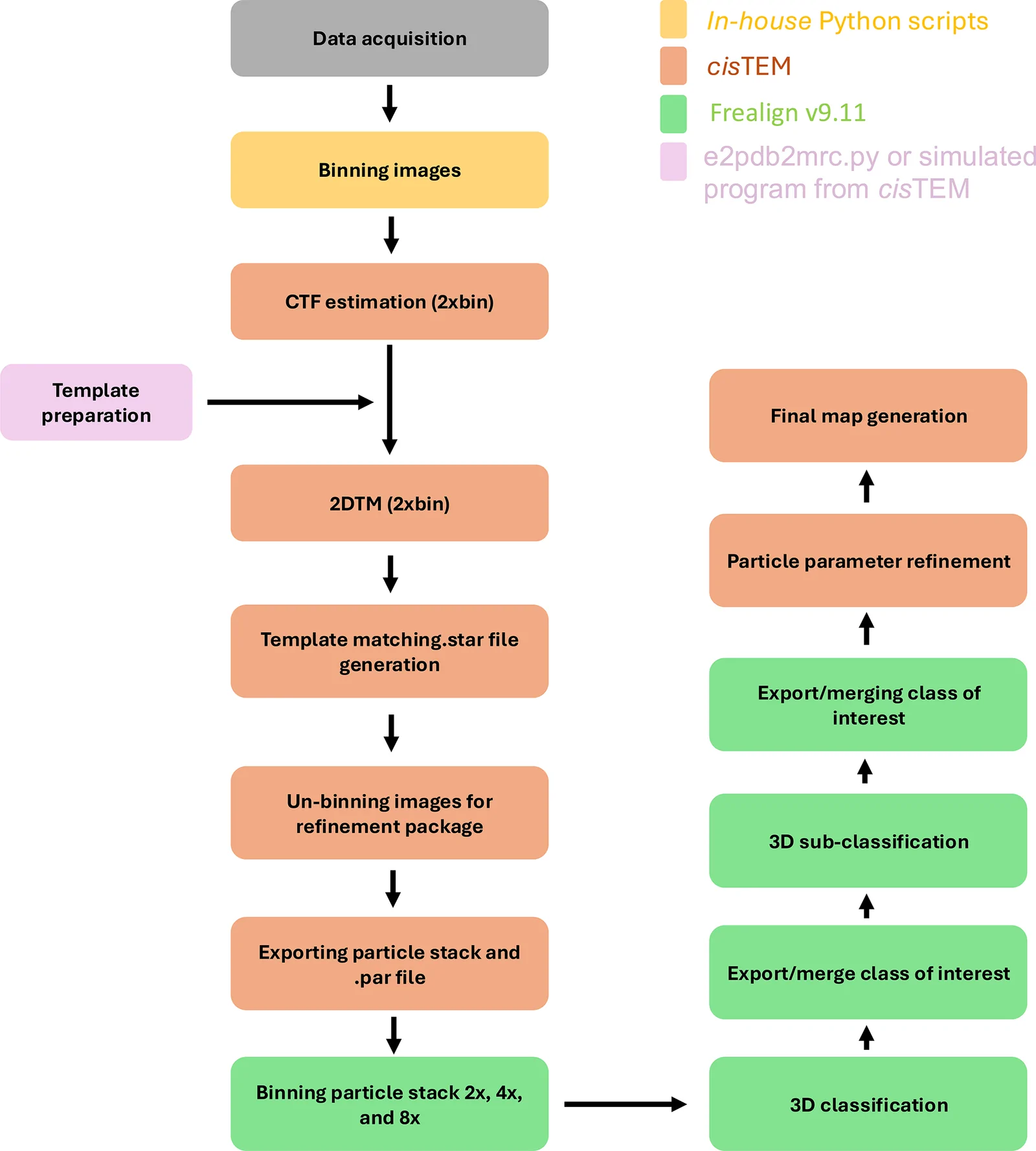
Data-processing workflow. Micrographs were binned using in-house Python script and then contrast transfer function (CTF) parameters were estimated from 2× binned micrographs using *cis*TEM, followed by two-dimensional template matching (2DTM) on binned data. To prepare the templates, we initially used both e2pdb2mrc.py and *cis*TEM’s simulate program to generate templates (see Methods). Subsequently, we switched to exclusively using the simulate program since it performed slightly better. Our goal in determining and optimizing search parameters for 2DTM was a balance between particle search precision and computational cost (see Methods). In practice, users must weigh the benefit of finer sampling against the substantial increase in runtime, particularly for large datasets. In some cases, the defocus search was turned off, and it was sufficient to use in-plane and out-of-plane angular steps of approximately 4.5° and 3.5°, respectively, and binned images with ~2 Å/pixel, to still obtain enough matches to obtain high-resolution reconstructions. To determine when defocus search was needed, micrographs were selected based on their fit resolution obtained during CTF estimation. From each dataset, we selected 10 micrographs representing the highest and lowest fit resolutions. Template matching was then performed using identical parameters, once with defocus search enabled and once with it disabled. The number of detected particles for each micrograph under both conditions was compared. When a significant difference was observed, most commonly for icy micrographs with low fit resolution, we enabled defocus search for that group of images. We found that these images/datasets appeared to have a higher background compared to *in vitro* reconstituted samples but less than *in situ* samples. The template matching results from these micrographs were subsequently combined with results from groups processed with defocus search disabled. After 2DTM, .star files were generated using *cis*TEM. The pixel size and directory to un-binned images were changed in this .star file, now called edited.star. A dummy project with a small group of micrographs was made and after performing 2DTM and generating a dummy .star file, the dummy .star file was substituted with edited .star. Then, we generated the refinement package, corresponding particle stacks, and .par files. Un-binning refers to generating particle stacks from the original micrographs at the original pixel size. Particle stacks and .par files were exported from *cis*TEM, and to speed up processing, binned image stacks (e.g. 2×, 4×, or 8×) were prepared using resample .exe, which is part of the Frealign v9.11 distribution. Initial 3D classification was carried out in Frealign v9.11. For each resulting class, per-class particle stacks and .star files were generated, and subsequent rounds of focused or global 3D classification were performed (see Methods). Software packages used at each step are indicated by color coding.

Particles were picked in the collected datasets using a high-resolution template obtained from a vacant (i.e. without translation factors) mammalian 60S ribosomal subunit (Jaako et al., 2022), and they were classified by 3D maximum-likelihood classification and a focus mask on the ribosomal A site (Methods; Figure 2—figure supplements 1–3). Classification yielded primarily 80S ribosomes, and most 60S particles contained additional bound factors, underscoring that picking with the vacant 60S template yielded largely template-unbiased results (Figure 2—figure supplement 1). This result echoes our concurrent finding that using 40S or partial 40S templates yields a variety of initiation complexes and 80S classes, revealing densities beyond those in the template (Zottig et al., 2025). The reconstruction of individual maps achieved a resolution of ~2.2 Å for the larger BSC-1 dataset (~525,000 particles) and ~3 Å for each of the MCF-7 datasets featuring much lower numbers of particles (~80,000).

**Figure 2.**
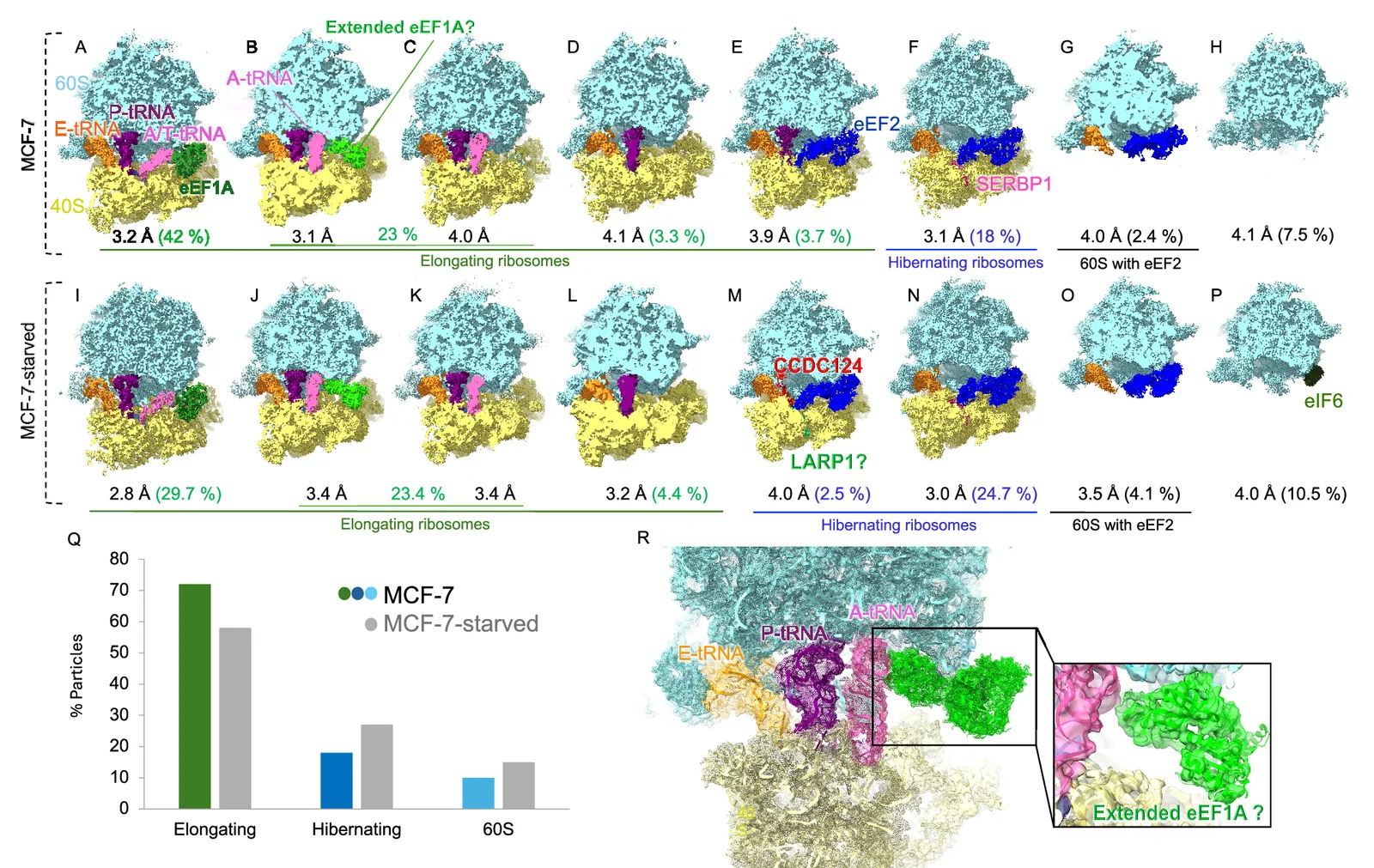
Ribosome and 60S particle distributions in control MCF-7 and starved MCF-7 cells. (**A–H**) Eight cryogenic electron microscopy (cryo-EM) maps correspond to elongating ribosomes (**A–E**): codon sampling with eEF1A and A/T tRNA (**A**), non-rotated with A/A, P/P, E/E tRNAs and putative extended eEF1A (**B**), non-rotated pre-translocation with A/A, P/P, E/E tRNAs (**C**), rotated pre-translocation with hybrid-state A/P and P/E tRNAs (**D**), and post-translocation ribosome with eEF2, P, and E tRNAs (**E**); hibernating rotated ribosome with eEF2, SERBP1, and P/E tRNA (**F**); 60S subunits with eEF2 and E-tRNA (**G**); and vacant 60S subunits (**H**). (**I–P**) Eight cryo-EM maps corresponding to nutrient-deprived MCF-7 cell lysates comprise elongation states (**I–L**) similar to those in panels A–D; hibernating head-swiveled state with eEF2, CCDC124, pe/E tRNA, and putative La-related protein 1 (LARP1) (**M**), hibernating rotated ribosome with eEF2, SERBP1, and P/E tRNA (**N**); 60S with eEF2 and E-tRNA (**O**); 60S with eIF6 (**P**). (**Q**) Particle distributions among functional ribosome states in normal and nutrient-deprived MCF-7 cells. (**R**) Putative extended eEF1A conformation; the close-up box shows density with the extended eEF1A model fitted (green; PDB ID:8B6Z; Gemmer et al., 2023).

Data obtained from MCF-7 and BSC-1 grown under normal growth conditions yielded predominantly translation elongation states containing mRNA and tRNAs (~70% in MCF-7 and ~60% in BSC-1, Supplementary file 1B). Most of these particles are in an mRNA-decoding state, containing canonical eukaryotic elongation factor 1A (eEF1A) with A/T tRNA (A/ternary complex) docked into the small-subunit A site or with density resembling extended eEF1A (Gemmer et al., 2023) with A, P, and E tRNAs (Figure 2A, B, and R). The remaining elongation classes contain mRNA with at least two tRNAs within a non-rotated or rotated (post-translocation) ribosome. A translocated 80S ribosome contains eEF2 in the A site, in the presence of P and E tRNAs, similar to the post-translocation state (POST-3: PDB ID 6GZ5) (Flis et al., 2018; Figure 2E). These maps closely resemble the structures of cellular elongation-state ribosomes identified in other cell types via cryo-ET (Gemmer et al., 2023; Hoffmann et al., 2022; Xing et al., 2023) or cryo-EM (Carbone et al., 2021; Zheng et al., 2025; Behrmann et al., 2015; Demo et al., 2021). Notably, all MCF-7 ribosome and 60S classes contain ErbB3-binding protein 1 (EBP1) bound next to the polypeptide exit tunnel, whereas BSC-1 also features membrane-bound ribosomes with Sec translocon and translocon-associated protein (Figure 3—figure supplement 1). Previously implicated in transiently binding hibernating/vacant ribosomes and regulating translation under stress (Squatrito et al., 2006; Wells et al., 2020; Bhaskar et al., 2021), EBP1 appears to be a prevalent protein on both translating and non-translating ribosome populations.

**Figure 2—figure supplement 1.**
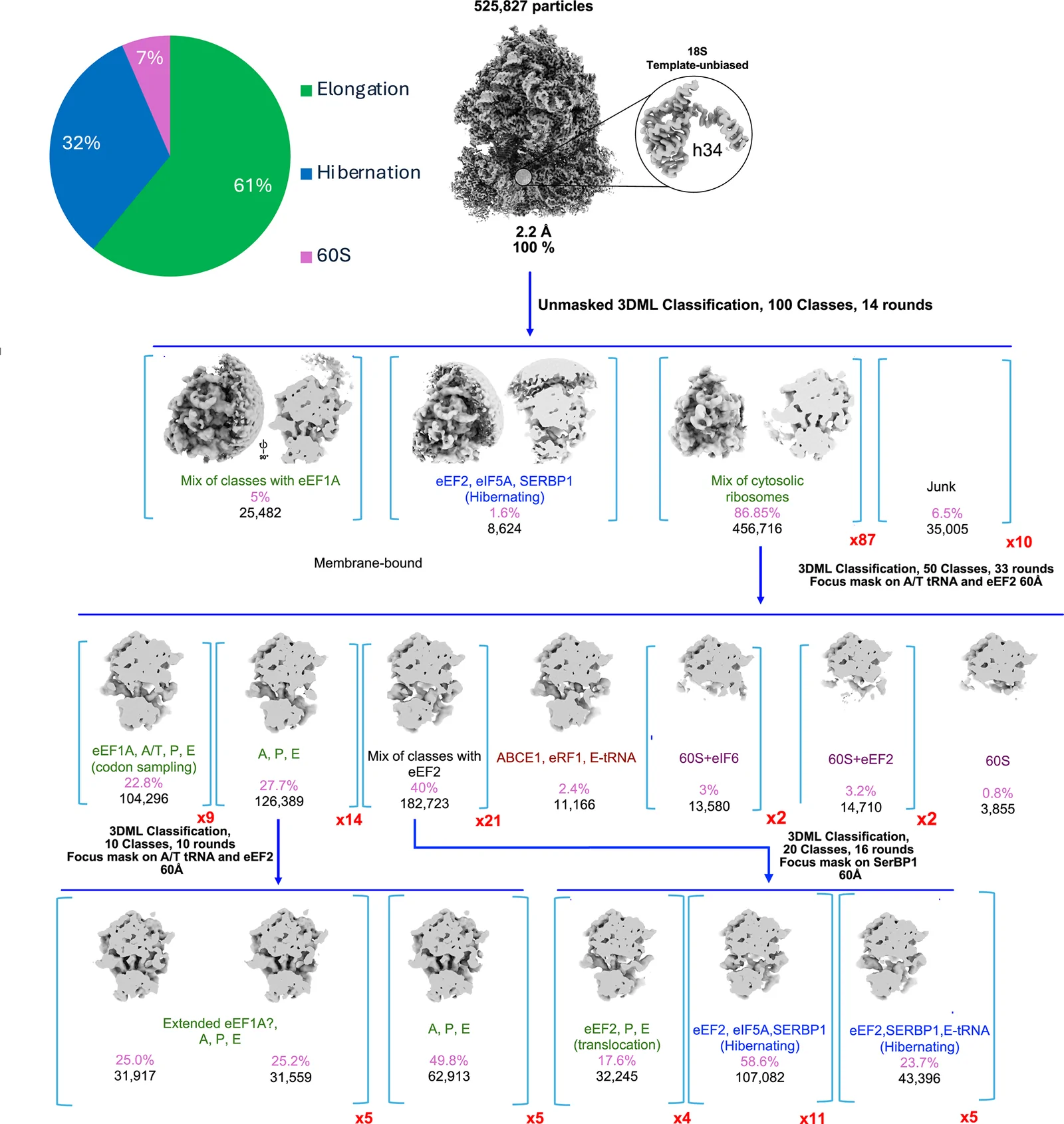
Maximum-likelihood three-dimensional (3D) classification of the dataset collected from BSC-1 cell extracts. Classification was performed on 8× binned stacks with particle alignment disabled.

**Figure 2—figure supplement 2.**
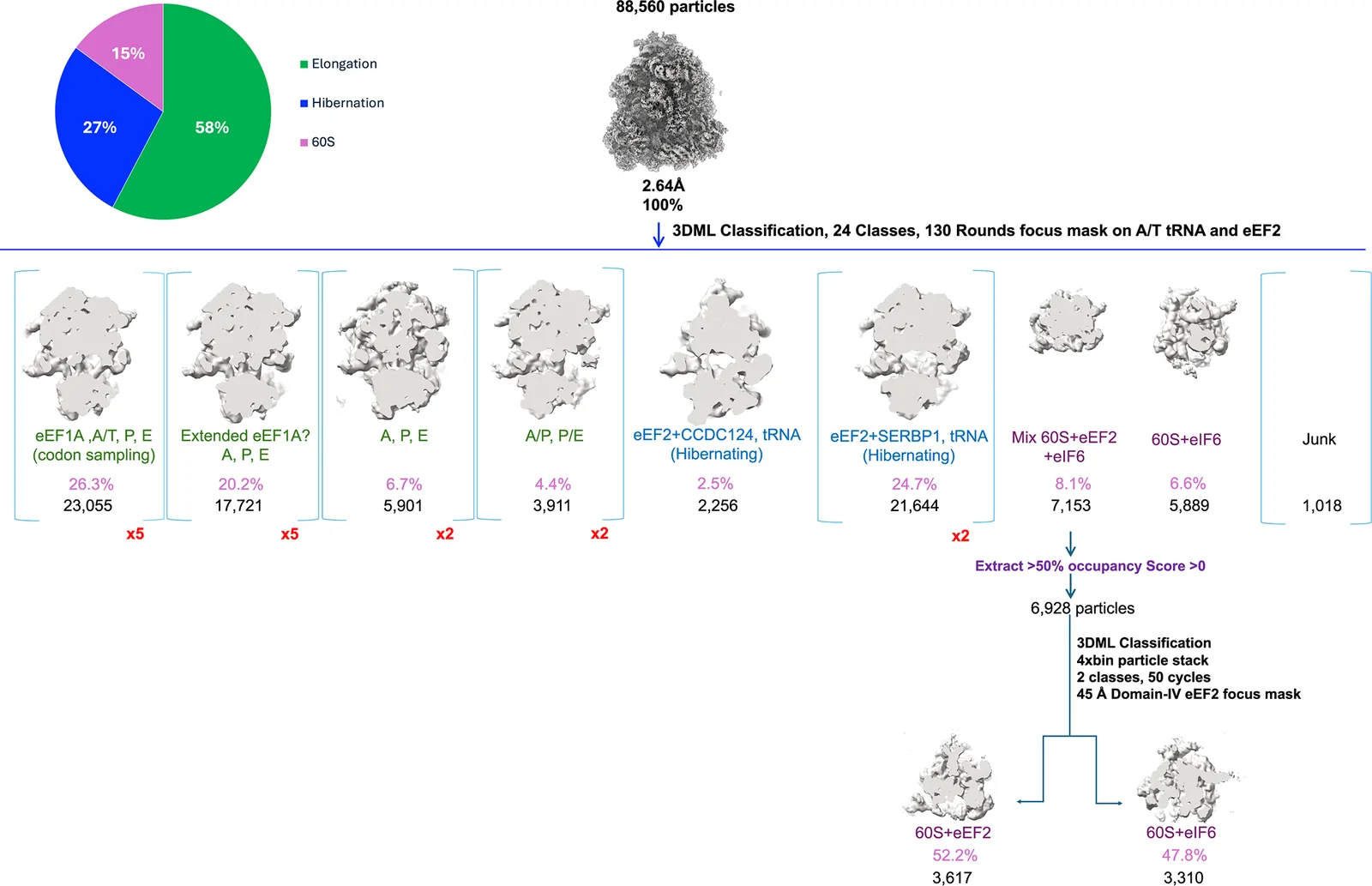
Maximum-likelihood three-dimensional (3D) classification of the nutrient-deprived MCF-7 extract. Classification was performed on 8× and 4× binned stacks with particle alignment disabled.

**Figure 2—figure supplement 3.**
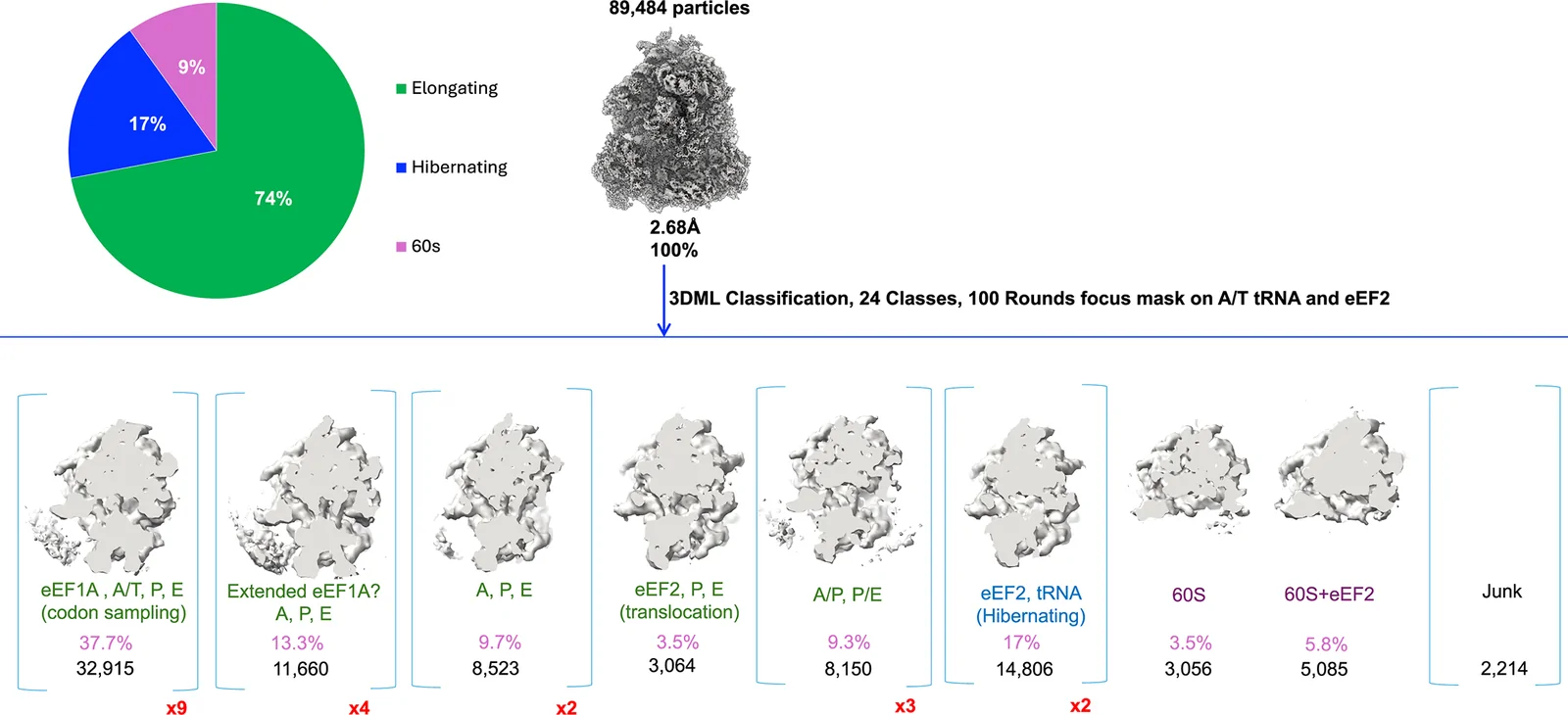
Maximum-likelihood three-dimensional (3D) classification of the MCF-7 extract cryo-EM data. Classification was performed on 8× and 4× binned stacks with particle alignment disabled.

In lysates made from nutrient-deprived MCF-7 cells, the percentage of elongation-like ribosomes bound to mRNA and tRNAs decreased to 58% (Figure 2I–L and Q), consistent with reduced translation. The abundance of unassociated 60S subunits increased from ~10% to ~15% (Figure 2G, H, O, P, and Q), in keeping with depletion in translation initiation. In both datasets, a fraction of 60S subunits is bound with elongation factor eEF2 (Figure 2G and O). This is unexpected because the primary function of eEF2 is to translocate mRNA and tRNAs on fully assembled 80S ribosomes (Kaul et al., 2011). Indeed, eEF2 has been predominantly found on 80S ribosomes in this and previous studies. We discuss the implications of eEF2 binding to the 60S subunit in the next section.

Another notable difference between normal and nutrient-deprived MCF-7 cells concerns non-translating 80S ribosomes formed without an mRNA, also termed ‘hibernating’ ribosomes (Brown et al., 2018; Ekemezie and Melnikov, 2024; Smith et al., 2022). Their content rises from 18% to 27% upon nutrient deprivation (Figure 2F, M, N, and Q). All hibernating ribosome classes feature eEF2. In addition, these maps contain Serpine1 mRNA-binding protein 1 (SERBP1) (Figure 2F and N), suggested to be the predominant eukaryotic hibernation factor (Smith et al., 2022). In nutrient-deprived cells, an additional hibernation class appeared, which contains coiled-coil-domain protein CCDC124 in the ribosomal P site (Figure 2M), resembling CCDC124-bound ribosomes purified from stressed cells (Wells et al., 2020) and in stressed ER-bound ribosomes (Gemmer et al., 2023). Helical density in the mRNA tunnel in this class does not align with SERBP1 and likely corresponds to a different protein, such as La-related protein 1 (LARP1), as discussed below. Similarly, the non-translating 80S classes contain eEF2 in the BSC-1 dataset, with SERBP1 being the second most abundant hibernation factor with ~32% of the total population (Figure 2—figure supplement 1).

To further investigate the potential functional roles of eEF2 on 60S subunits and hibernating 80S ribosomes, we obtained larger datasets of commercial RRLs, which contain larger fractions of non-translating 80S ribosomes.

### RRL contains non-translating ribosomes with eEF2 and SERBP1 or LARP1

**Figure 3.**
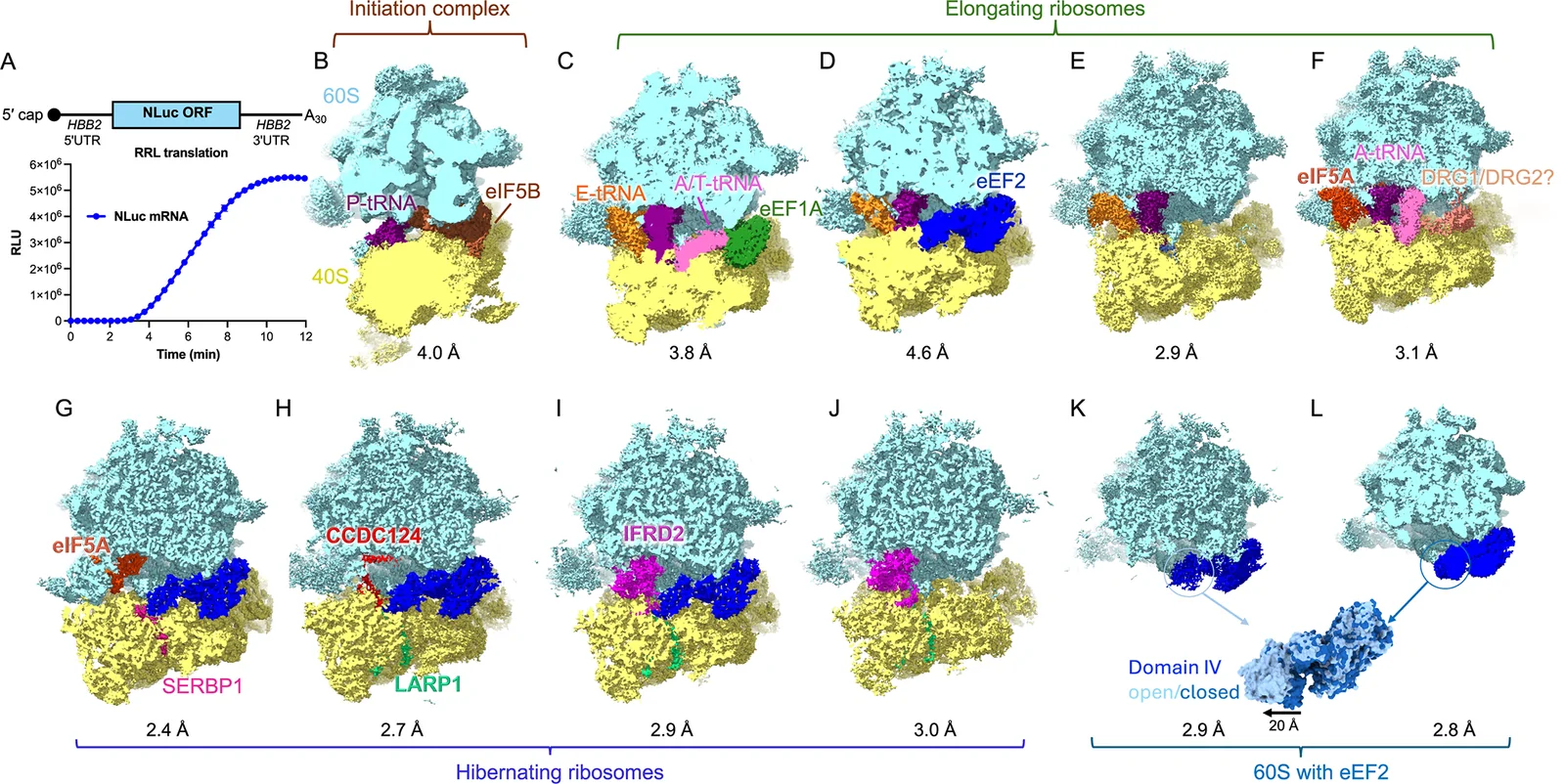
*In extracto* cryogenic electron microscopy (cryo-EM) of rabbit reticulocyte lysates. (**A**) NLuc mRNA construct used to monitor NanoLuciferase translation (top) and real-time translation kinetics (bottom). The graph represents the mean ± SEM from three independent experiments. (**B–L**) Cryo-EM maps of the major classes resulting from three-dimensional (3D) classification: (**B**) initiation complex with eIF5B; (**C–F**) elongating ribosomes: codon-sampling with eEF1A (**C**), post-translocation with eEF2 ap/P and pe/E tRNAs (**D**), pre-translocation hybrid state (**E**), and with GTPase density putatively assigned to DRG1/DRG2 next to A/A tRNA (**F**). (**G–J**) Hibernating ribosomes: 40S-rotated ribosome with eEF2, eIF5A, and SERBP1 (**G**); 40S-head-swiveled ribosome with eEF2, CCDC124, and LARP1 in the mRNA tunnel (**H**); 40S head-swiveled with eEF2, IFRD2, and LARP1 in the mRNA tunnel (**I**); 40S-head-swiveled with IFRD2 and LARP1 (**J**); 60S subunit with eEF2 domain IV open (**K**); 60S with eEF2 domain IV closed (**L**): the extent of domain IV movement in 60S-bound eEF2.

2DTM with the 60S template, as described above, yielded ~1.1 million particles in cryo-EM data collected from commercially available RRLs (Figure 3—figure supplement 2). Such preparations are derived from nuclease-treated lysates to inhibit translation from endogenous mRNAs, allowing studies of reporter constructs (Susorov et al., 2020). Indeed, we collected data from translationally active lysates, as evidenced by luminescence from a NanoLuciferase reporter mRNA (Figure 3A). After translating a reporter mRNA for 10 min, only ~12.4% of ribosomes are bound with mRNA and tRNAs, indicating a depletion of translationally active states (Figure 3, Figure 3—figure supplement 2). This finding coincides with biochemical estimates of ~10% of RRL ribosomes remaining on a reporter mRNA following a burst of translation (Susorov et al., 2020). The predominant elongating ribosomes feature eEF1A, A/T, P, and E tRNAs (Figure 3C) or eEF2 with chimeric ap/P and pe/E tRNAs resembling the nearly post-translocated state (POST-2: PDB ID 6GZ4 Flis et al., 2018; Figure 3D). In addition, an unusual class emerged with density at the GTPase center that could not be assigned to canonical elongation factors (Figure 3F, Figure 3—figure supplement 3B). The shape of the density most closely resembles developmentally regulated GTP-binding proteins DRG1 or DRG2, whose homologs (Rbg1/2) were recently visualized on yeast ribosomes (Zeng et al., 2021; Pochopien et al., 2021). The protein interacts with the A-site tRNA—as if stabilizing the acceptor arm docked at the peptidyl transferase center—consistent with the proposed role of DRG and Rbg proteins in restoring elongation on stalling-prone sequences, such as poly-proline (Zeng et al., 2021; Daugeron et al., 2011). Whereas the overall shape and secondary structure resemble DRG1 or DRG2, the local resolution is insufficient to distinguish between these or other similarly structured proteins. Both yeast and mammalian counterparts are reported to function with a companion factor (Tma146p or Gir2 in yeast; or DFRP1 and DFRP2 in mammals), but our maps do not contain density that could correspond to DFRP1/2 near the putative DRG1/2 density. Future work will elucidate the function of these or other DRG-like GTPases in the context of an elongation complex.

**Figure 3—figure supplement 1.**
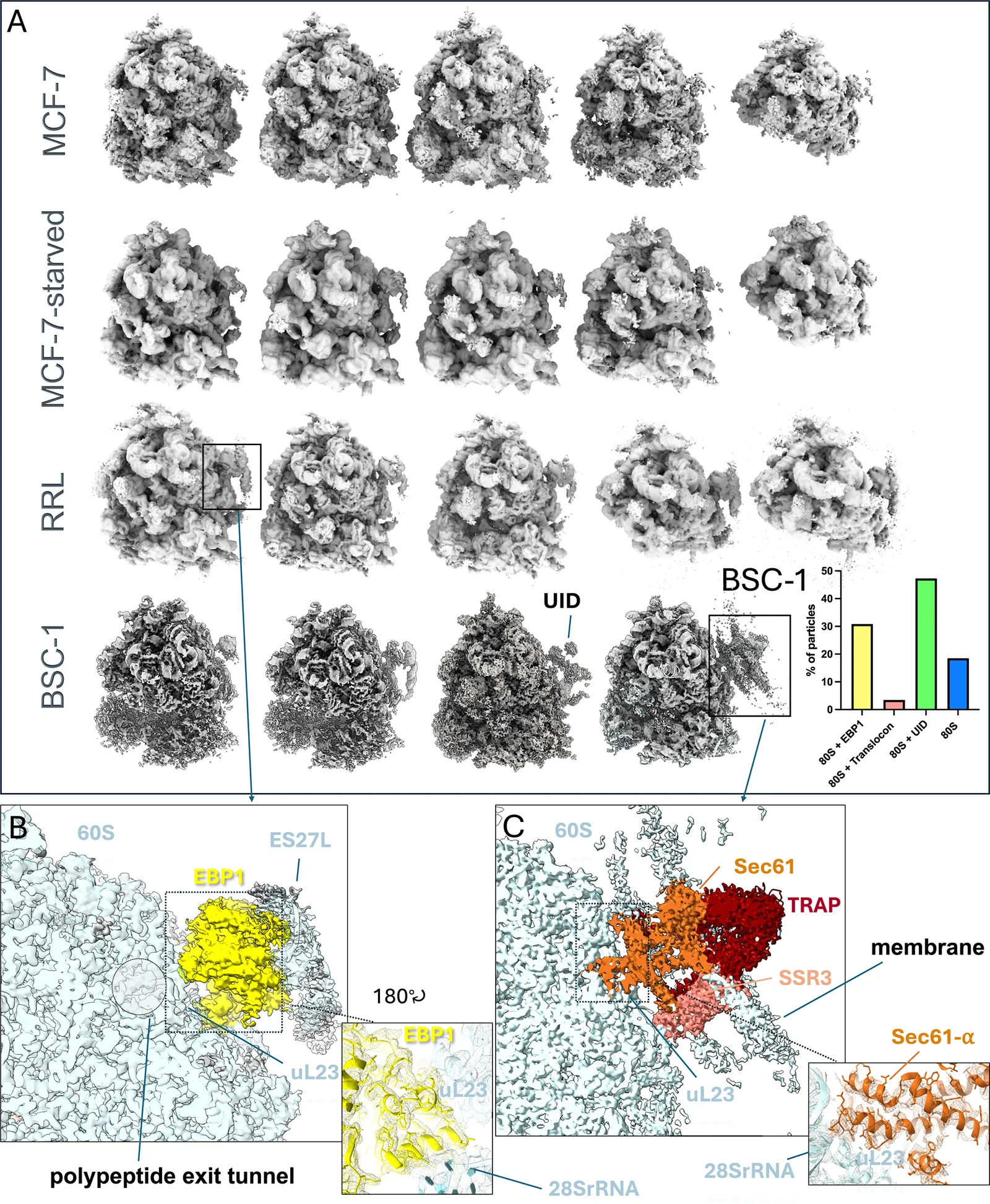
Ribosome classes feature EBP1, the Sec translocon, or unidentified density (UID) next to the polypeptide exit tunnel. (**A**) Representative classes obtained by three-dimensional (3D) classifications with a mask covering the binding site of EBP1 yield 80S predominantly bound with EBP1 in MCF-7 and rabbit reticulocyte lysate (RRL), and additional classes consistent with the Sec translocon or unidentified (likely heterogeneous) densities in BSC-1 lysates. The bar graph shows particle percentages corresponding to different classes in BSC-1. (**B**) A close-up view of EBP1 in RRL 80S ribosomes with eEF2, eIF5A, and SERBP1. (**C**) A close-up view of membrane-bound ribosomes in BSC-1, with the structural models of Sec61, translocon-associated protein (TRAP), and signal sequence receptor subunit 3 (SSR3) shown (PDB ID 8BTK; Jaskolowski et al., 2023).

**Figure 3—figure supplement 2.**
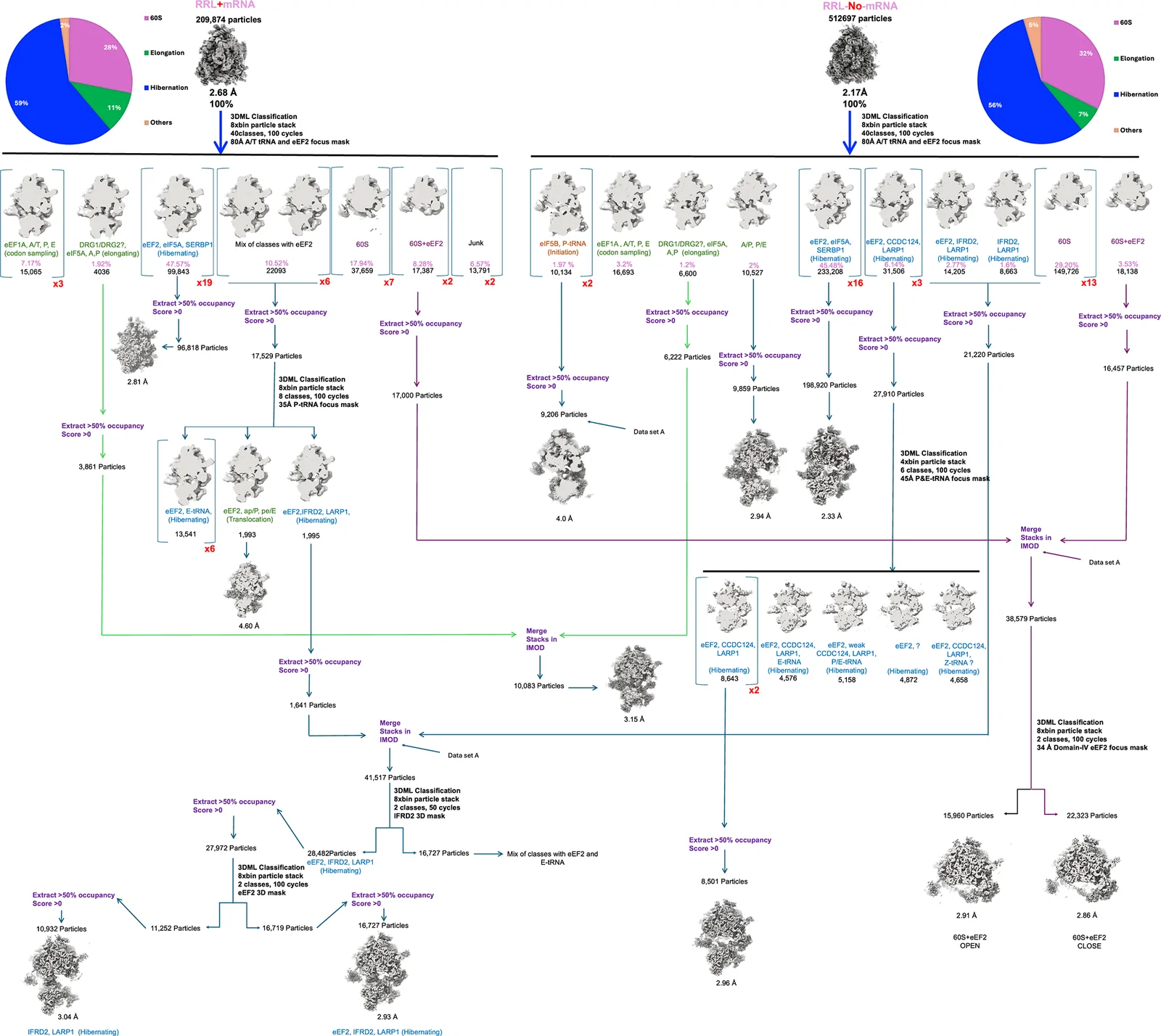
Maximum-likelihood three-dimensional (3D) classification of rabbit reticulocyte lysate (RRL) datasets with or without nanoluciferase-encoding mRNA. Classification was performed on 8× binned stacks with particle alignment disabled.

**Figure 3—figure supplement 3.**
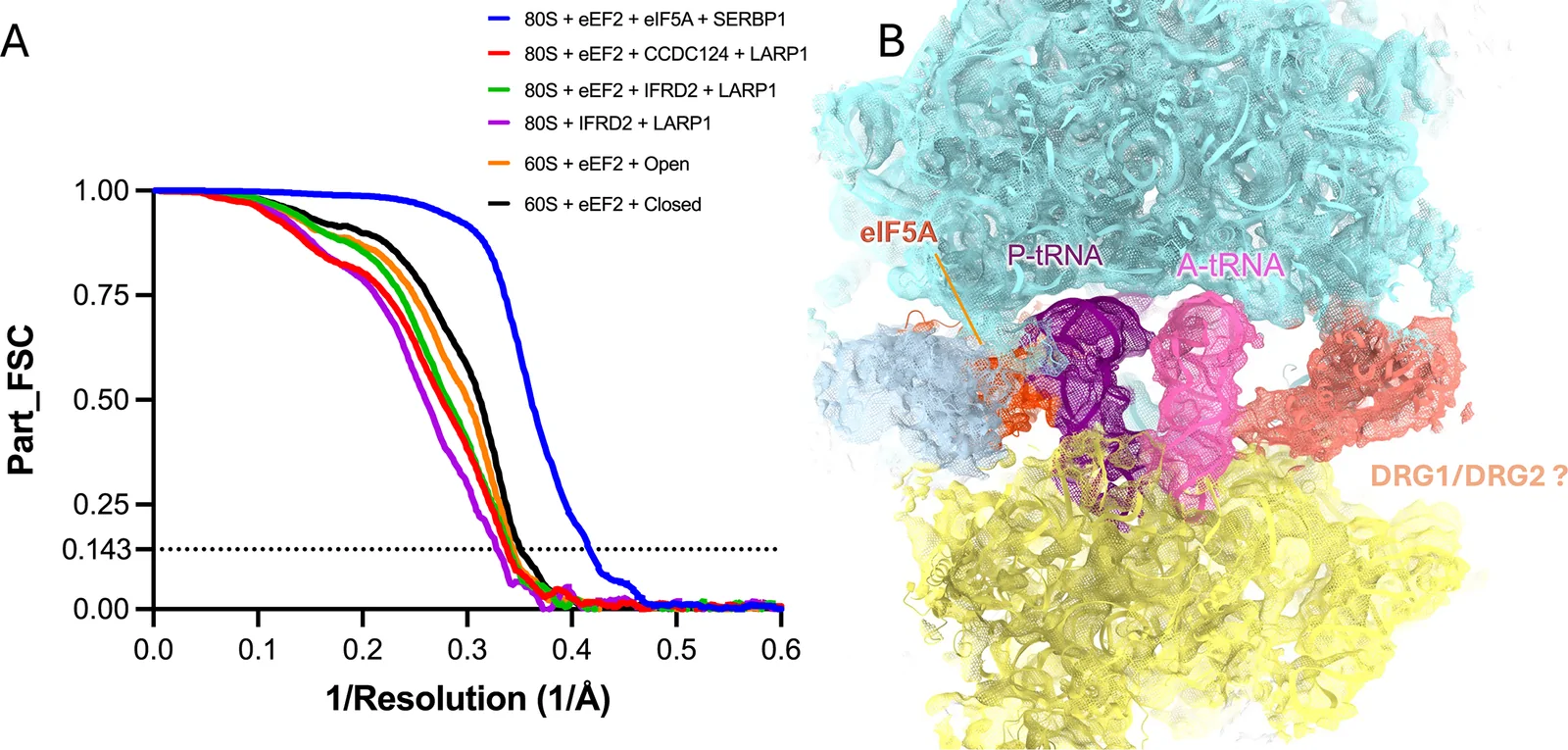
RRL dataset cryo-EM map statistics and a reconstruction example. (**A**) Fourier shell correlation (FSC) curves for rabbit reticulocyte lysate (RRL)-derived maps (masked), as a function of inverse resolution. (**B**) RRL 80S ribosomes with putative DRG1 (or DRG2), eIF5A in the E site, and tRNAs in the A and P sites.

**Figure 3—figure supplement 4.**
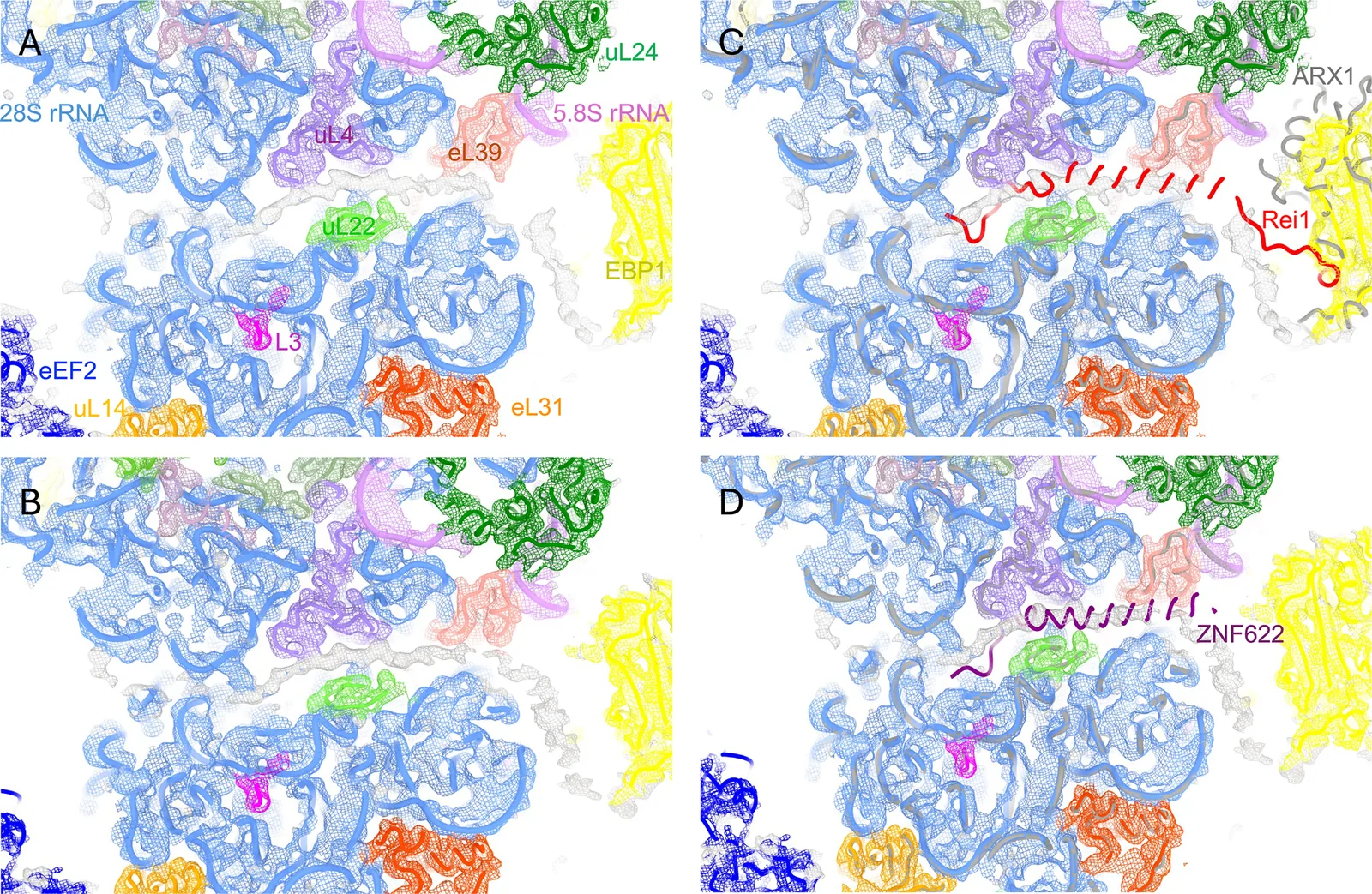
60S subunit polypeptide tunnel is occupied with an uncharacterized protein resembling Rei1 (rabbit reticulocyte lysate [RRL]). (**A**) Cryogenic electron microscopy (cryo-EM) map (mesh) and model of 60S with eEF2 open. Density corresponding to the tunnel protein is shown in gray. (**B**) Cryo-EM map (mesh) and model of 60S with eEF2 closed. (**C**) Superposition of the yeast 60S biogenesis factor Rei1 (PDB ID 5APN; Greber et al., 2016) with the tunnel density (gray). Rei1 is shown in red, and the rest of the yeast 60S model is in gray. Cryo-EM maps were softened with the B-factor of 50 Å^2^. 60S structure superpositions were performed in ChimeraX. (**D**) Superposition of the mammalian 60S biogenesis factor ZNF622 (PDB ID 9GMO; Akers et al., 2025) with the unidentified tunnel density (gray). ZNF622 is shown in purple, and the rest of the superposed 60S model is in gray.

**Figure 3—figure supplement 5.**
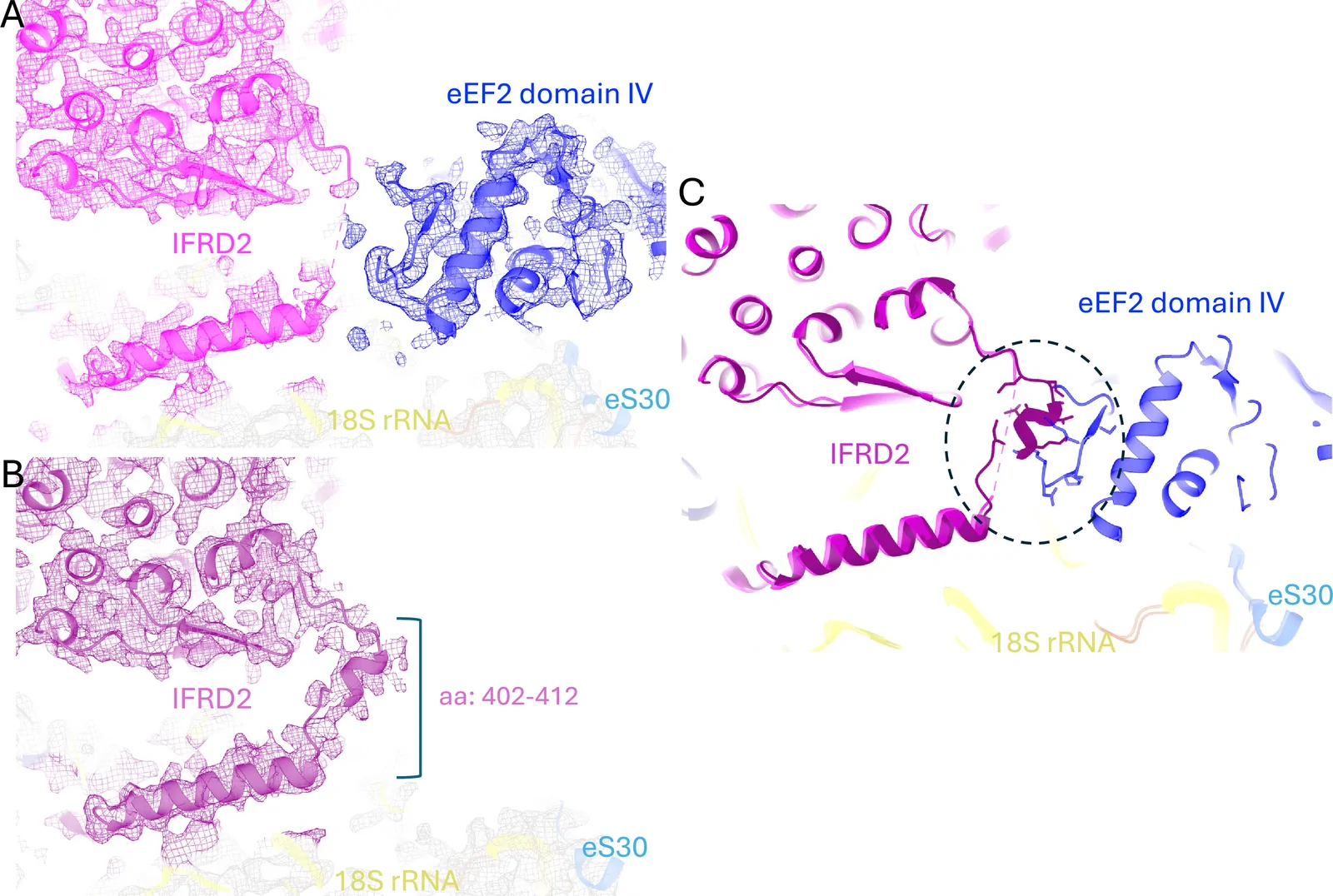
The linker helix (aa 402–412) of IFRD2 adopts different conformations in the presence and in the absence of eEF2. (**A, B**) Cryogenic electron microscopy (cryo-EM) maps (mesh) and models of 80S with LARP1 and IFRD2, in the presence of eEF2 (**A**) and in the absence of eEF2 (**B**). (**C**) Superposition of models from panels A and B. IFRD2 in the presence of eEF2 is shown in magenta, and in the absence of eEF2 is shown in purple. The steric clash between the IFRD2 linker helix and domain IV of eEF2 is indicated by a circle.

Most ribosome particles in RRL—nearly 60%—are in non-translating states, evidenced by the absence of mRNA density. Like in primate lysates described above, eEF2 is the predominant translation factor bound to 97% of hibernating ribosomes (Figure 3). The most abundant class represents nearly half of hibernating ribosomes and adopts a partially rotated ribosome conformation. Here, the small subunit body is rotated by ~5° and the head is swiveled by ~16° relative to a non-rotated decoding state (5LZS; Table C, Supplementary file 1C), so that the ribosome resembles a late translocating ribosome with two ‘chimeric’ ap/P and pe/E tRNAs and eEF2 or EF-G (Ramrath et al., 2013). The most abundant hibernating ribosome, however, features no tRNA, and instead, three proteins overlap with the three tRNA-binding sites: eEF2 spanning from the sarcin-ricin loop (SRL) on the large subunit to the decoding center (DC) on the small subunit, SERBP1 occupying the mRNA tunnel extending into the P site, and eIF5A extending from the E site into the peptidyl transferase center (PTC) (Figure 3G). As such, the ribosomal RNA at the key functional centers—SRL, DC, and PTC—are inaccessible to other proteins, which was proposed to serve as protection against nucleolytic cleavage that may occur in stressed cells (Koli and Shetty, 2024; Shedlovskiy et al., 2017; Willi et al., 2018; Lipońska and Yap, 2021).

**Figure 4.**
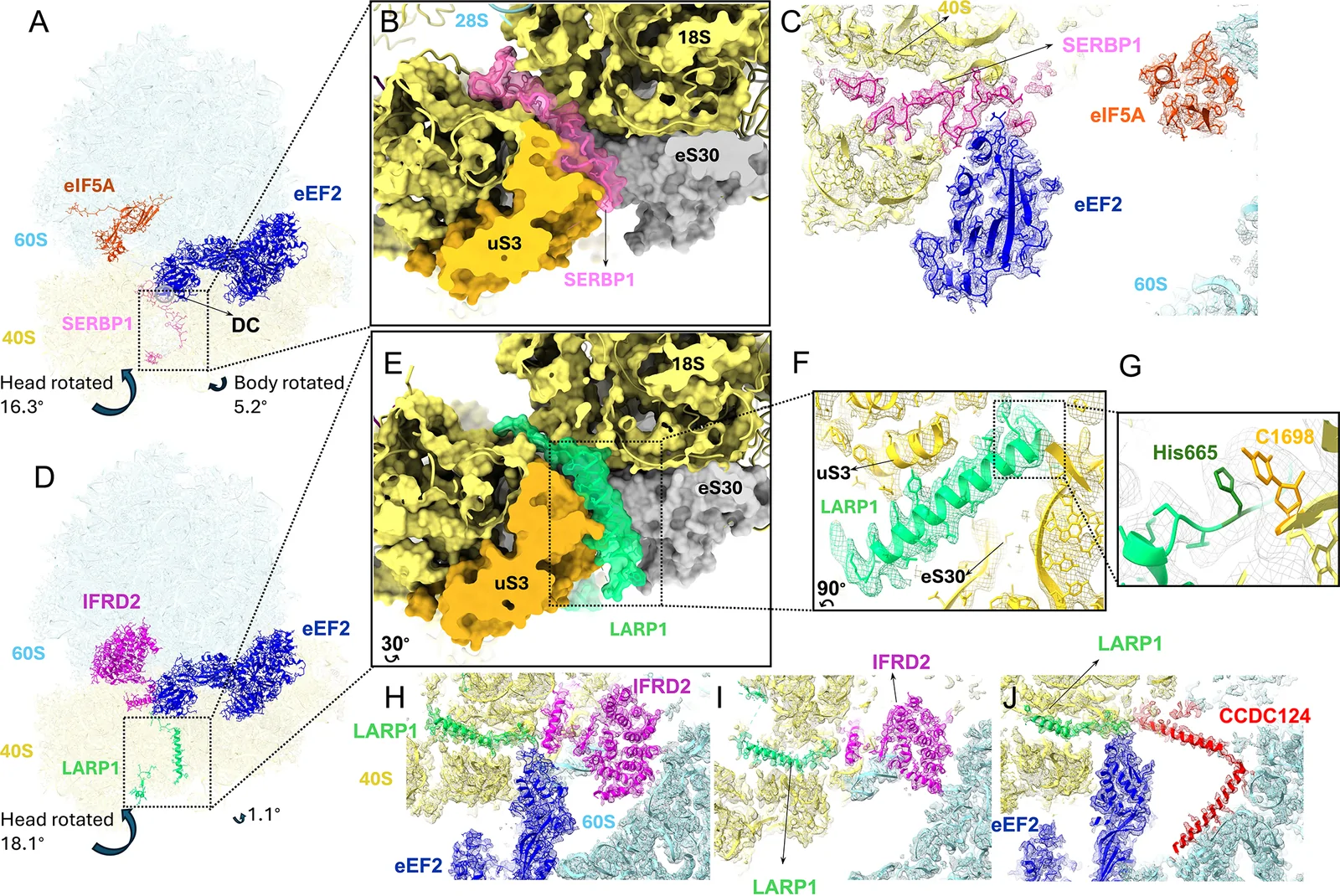
The occupancy of the mRNA tunnel in hibernating ribosomes from rabbit reticulocyte lysate (RRL). (**A**) The 80S ribosome with eEF2, eIF5A, and SERBP1 (DC: decoding center). 40S body and head rotation degrees are shown relative to 80S with eEF1A•aa-tRNA and P-tRNA (PDB 5LZS). (**B**) Close-up view of cryogenic electron microscopy (cryo-EM) density for SERBP1 in the mRNA tunnel. (**C**) Density for SERBP1 interaction with eEF2. (**D**) The 80S ribosome with eEF2, IFRD2, and LARP1. (**E**) Close-up view of LARP1 density in the mRNA tunnel. (**F–G**) Views of the density for the LARP1 helix interacting with 40S ribosomal proteins and RNA. (**H–J**) Similar densities for LARP1 in CCDC124- and IFRD2-bound ribosomes.

Our classification also yielded three, to our knowledge previously unreported, states of hibernating ribosomes that adopt non-rotated conformations (body rotation –1°), in which the head of the small subunit is swiveled by ~18° (Figure 3H–J). Like in the predominant state described above, the ribosomal functional centers are protected by the simultaneous binding of eEF2 with CCDC124 (Figure 3H) or eEF2 with IFRD2 (interferon-related developmental regulator 2; Figure 3I). Remarkably, helical density in the mRNA tunnels in these maps differs from the coiling SERBP1 (Figure 4) and most closely resembles LARP1 (residues 659–724), which was recently imaged by cryo-EM on vacant 40S ribosomes (Saba et al., 2024; Wolin et al., 2025). LARP1 is a component of RRL (Absmeier et al., 2023) and is implicated in translation initiation on pyrimidine-rich mRNAs (Fonseca et al., 2015). LARP1 was shown to bind 80S ribosomes (Saba et al., 2024), although no structure was reported.

**Figure 4—figure supplement 1.**
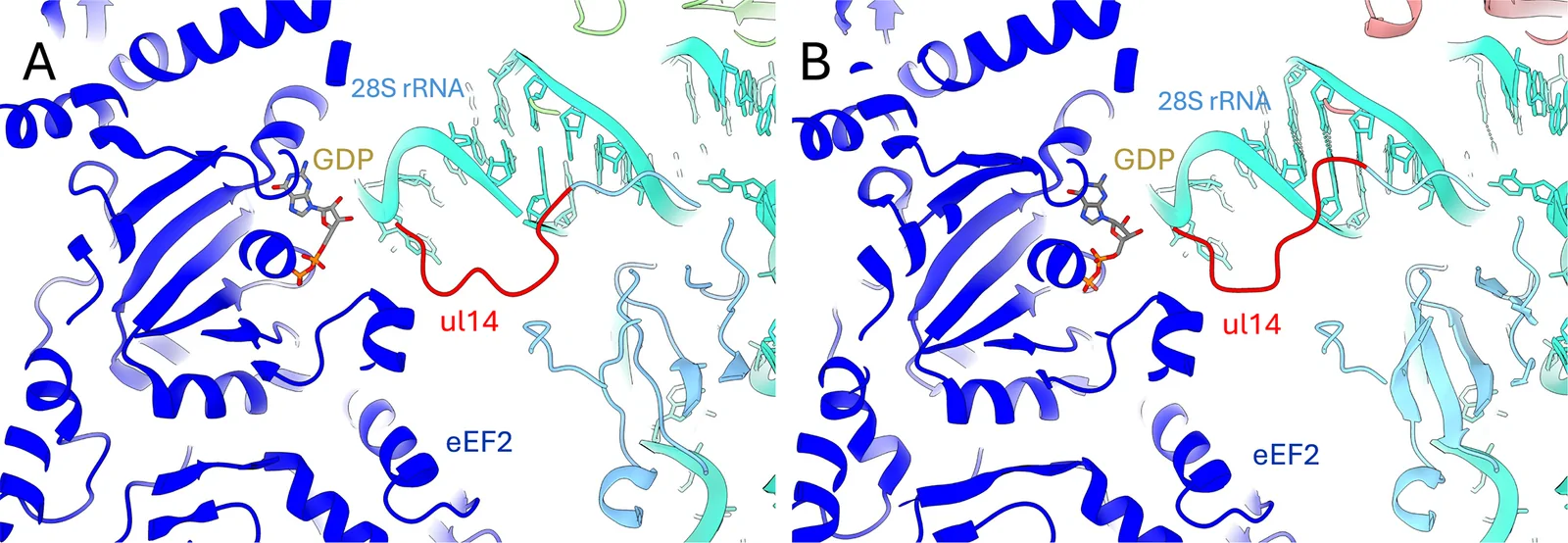
N-terminal tail of uL14 in the presence of eEF2. (**A**) Structure with eIF5A, eEF2, and SERBP1 (this work, rabbit reticulocyte lysate [RRL]). (**B**) *Gallus gallus* eEF2 bound to translocation-like ribosomes (PDB ID 8Q87 Nurullina et al., 2024).

**Figure 4—figure supplement 2.**
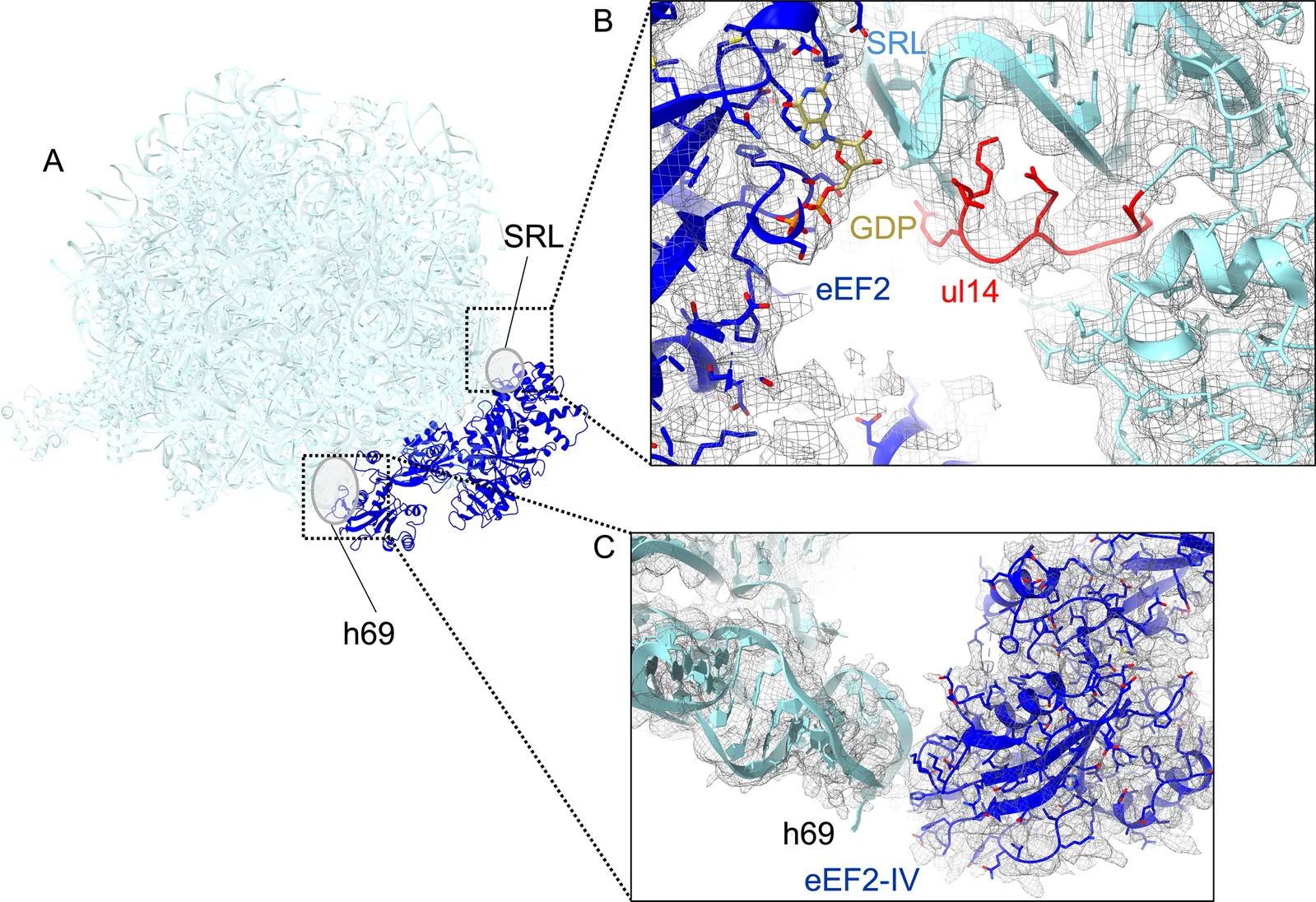
Interaction of eEF2 with the isolated 60S subunit (rabbit reticulocyte lysate [RRL]). (**A**) eEF2 in a compact conformation (relative to the second, more open conformation observed in this work). (**B**) Interaction with the sarcin-ricin loop (SRL) and uL14. The cryogenic electron microscopy (cryo-EM) map (mesh) was softened with a B-factor of 25 Å^2^. (**C**) Domain IV of eEF2 binds next to helix 69 of 28S rRNA. The map was softened with a B-factor of 30 Å^2^.

Our finding suggests that instead of SERBP1, LARP1 or another protein with LARP1-like binding mode binds the hibernating 80S ribosomes adopting a head-swiveled state in the presence of eEF2 with CCDC124 or IFRD2. Since density features closely resemble the abundant LARP-family protein LARP1 (Figure 4) and LARP1 is regulated during stress (see below), we hypothesize that the density corresponds to LARP1. The protein interacts simultaneously with eEF2 and CCDC124 or IFRD2 in the DC (Figure 4), and this interaction likely accounts for the ribosome rotational state. The functional interplay between CCDC124 and LARP1, as well as between IFRD2 and LARP1, is interesting because their simultaneous recruitment to the hibernating ribosomes may enhance their individual roles as translational repressors during stress and may be cell- or tissue-specific. In addition, as LARP1 controls translation of mRNAs with a TOP motif, which encode ribosomal proteins, the protein’s recruitment into hibernating ribosomes may help downregulate ribosome expression during stress. Finally, the binding of the extra-ribosomal DM15 domain of LARP1 to TOP mRNAs may prepare these mRNAs for translation upon stress relief. Indeed, like SERBP1, LARP1 is the substrate of TORC1 (Hong et al., 2017). TORC1 was shown to stimulate translation at least in part by activating dormant ribosomes via phosphorylation of Stm1, the yeast counterpart of SERBP1 (Shetty et al., 2023). LARP1 binding to and release from 80S ribosomes may similarly be controlled by TORC1 and Akt/S6K1 (Hong et al., 2017) to switch between stress and proliferation conditions.

The hibernating ribosomes imaged directly in RRL lysates differ from ribosomes purified from RRL (Brown et al., 2018) in that the latter only contained eEF2 with SERBP1, or tRNA with or without IFRD2, but not eIF5A, or eEF2 with IFRD2/CCDC124, and LARP1. While some of these proteins may be low in abundance and may have fallen below the detection limit of the previous study, the absence of highly abundant eIF5A and eEF2 in the corresponding classes indicates that they likely dissociated from ribosomes during purification. Indeed, fractions of IFRD2-bound ribosomes with or without eEF2 in the RRL lysate data (Figure 3I–J) suggest transient eEF2 interactions with some types of hibernating ribosomes. An internal loop of IFRD2 (aa 402–412), ordered in the absence of eEF2, is displaced by the tip of eEF2 domain IV, suggesting that steric hindrance may account for the partial occupancy of eEF2 in IFRD2-bound ribosomes (Figure 3—figure supplement 5). Nevertheless, the most abundant class with eEF2, eIF5A, and SERBP1 is readily detected in cellular cryo-EM and single-particle cryo-EM studies from other mammalian cell lines (Du et al., 2024), whereas no CCDC124- or IFRD2-bound ribosomes have been reported with LARP1. These findings underscore the importance of imaging ribosomes within cells or lysates to enable the identification of cellular interactions.

The 60S classes contain eEF2•GDP bound next to the SRL, featuring open (Figure 3J) and compact (Figure 3K) conformations. eEF2 and its bacterial counterpart EF-G rearrange between open and compact conformations on the complete translocating ribosome (Carbone et al., 2021; Xue et al., 2022) or when free in solution (Salsi et al., 2015). Capturing these conformations in the 60S subunit likely reflects an equilibrium that eEF2 is spontaneously sampling, resembling that of free EF-G (Ling and Ermolenko, 2016). A potential role of 60S•eEF2 complexes may be to protect the SRL from damage in free subunits under stress.

In addition, 60S classes feature density spanning the polypeptide tunnel (Figure 3—figure supplement 4A–B), resembling 60S maturation factor ZNF622 in mammals (Akers et al., 2025) and its homolog Rei1 in yeast (Greber et al., 2016). The density differs from both factors on the 60S periphery, where it approaches EBP1 (Figure 3—figure supplement 4C–D), suggesting a different conformational state of ZNF622 or another protein, whose role may be to occlude the PTC and tunnel periphery during 60S hibernation. Future studies will bring insights into the roles of the protein(s) and into the functions and transitions of 60S•eEF2 complexes to the pool of translating ribosomes.

### Structural basis for eEF2•GDP binding to hibernating ribosomes

The essential cellular function of eEF2, and of its better-studied bacterial counterpart EF-G, is to catalyze translocation of mRNA and tRNA during elongation (Korostelev, 2022; Rodnina et al., 2011; Voorhees and Ramakrishnan, 2013; Ling and Ermolenko, 2016; Noller, 2024). To this end, the translocase binds to a pre-translocation ribosome containing peptidyl-tRNA in the hybrid A/P state and deacyl-tRNA in the P/E state (in the tRNA hybrid-state nomenclature, the first letter denotes the position on the small subunit and the second letter on the large subunit). The ribosome adopts a rotated state in which the small subunit is rotated by ~10° relative to its position in a non-rotated ribosome harboring the ‘classical-state’ A and P tRNAs (aka A/A and P/P). Upon binding, the GTPase domain of eEF2/EF-G docks at the universally conserved SRL critical for triggering GTP hydrolysis (Shi et al., 2012; Gao et al., 2009; Abeyrathne et al., 2016; Carbone et al., 2021). The translocase domain (domain IV) is placed next to the A site on the small subunit. Due to the ribosome’s inherent propensity to undergo subunit rotation (Cornish et al., 2008), the spontaneous reversal of the small subunit results in the arrival of translocase domain IV to the A site (also known as the DC) and the movement of tRNA-mRNA helix from the A to P site on the small subunit (Ermolenko and Noller, 2011; Wasserman et al., 2016). The enzyme dissociates upon the completion of subunit rotation and translocation after GTP hydrolysis and P_i_ release from the GTPase domain, resulting in the departure of this domain from SRL (Carbone et al., 2021).

While the roles of eEF2 and GTP hydrolysis in tRNA-mRNA translocation are reasonably well understood, their functions in ribosome hibernation remain a puzzle. Unlike the preferred translocase substrate—the pre-translocation ribosome—hibernating ribosomes do not contain peptidyl-tRNA or mRNA in the A site, which could stabilize eEF2 on the ribosome. Furthermore, previous studies captured eEF2 on hibernating ribosomes in the presence of SERBP1 in the DC, suggesting that the latter might contribute to eEF2 stabilization on the ribosome (Smith et al., 2022). Indeed, SERBP1, spanning the mRNA tunnel, contacts domain IV of eEF2 in the A site in this (Figure 4A–C) and previous studies (Smith et al., 2021). Our work, however, also identifies eEF2 on hibernating ribosomes in the presence of several other factors, and in their absence (on the 60S subunit), suggesting novel insights into the function of eEF2 on hibernating ribosomes.

In hibernating 80S ribosomes, eEF2 interacts with SERBP1 or LARP1 (Figure 4), arguing that the latter can replace SERBP1 in its function to stabilize eEF2 in the A site of the small subunit. By contrast, eEF2 binding to free 60S in this work, as well as binding of eEF2/EF-G to rotated ribosomes with a single tRNA (Brown et al., 2018; Flis et al., 2018; Carbone et al., 2021) placed away from domain IV, argue that the contact in the 40S A site is not necessary for eEF2 binding. Instead, binding on the 60S subunit may be the predominant interaction to stabilize eEF2.

**Figure 5.**
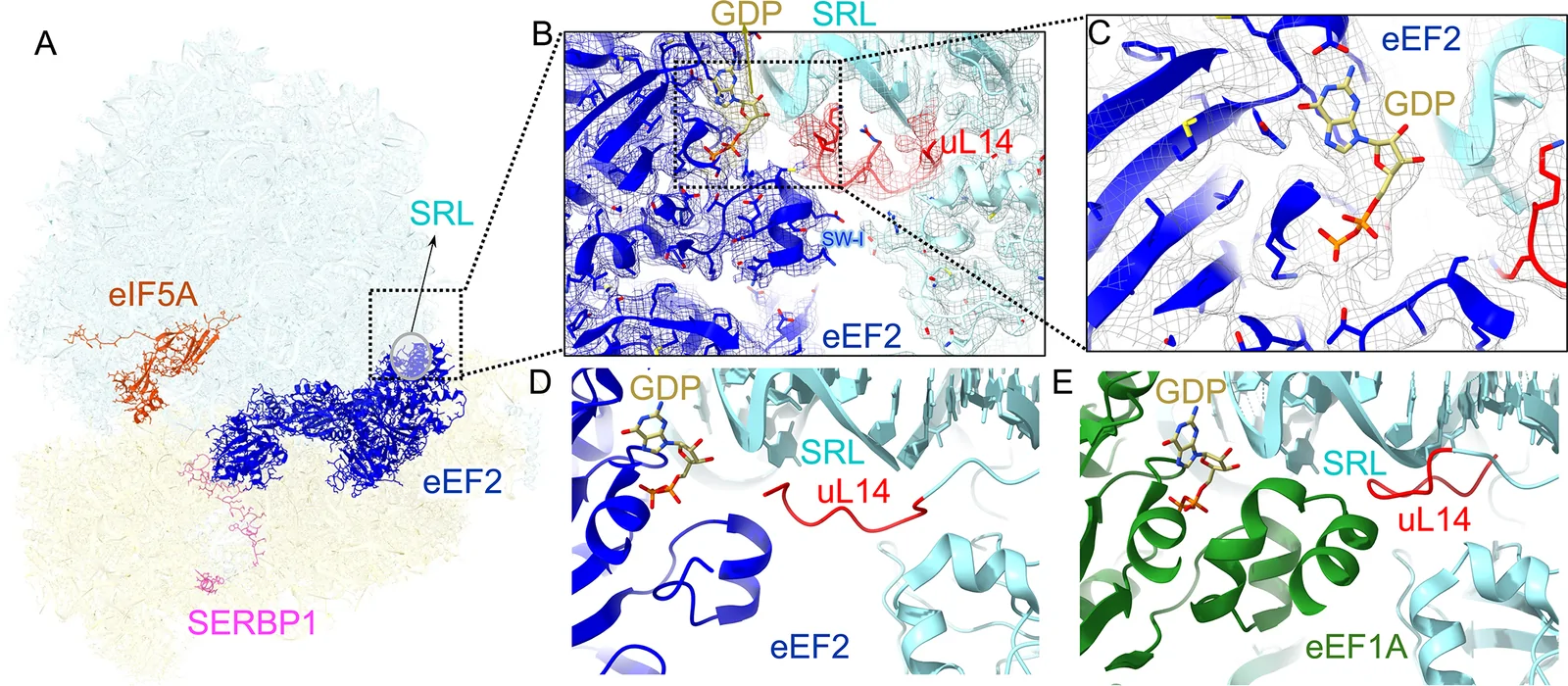
Interactions of eEF2 with the GTPase-activating center in hibernating ribosomes. (**A**) Overall view of the 80S structure with eEF2, eIF5A, and SERBP1. (**B**) Cryogenic electron microscopy (cryo-EM) density and model of the eEF2 GTPase center at the sarcin-ricin loop (SRL). (**C**) GDP density in the GTPase center of the predominant hibernating ribosomes. (**D, E**) Distinct conformations of the N-terminal tail of uL14, highlighted in red, in ribosomes bound with eEF2•eIF5A•SERBP1 (**D**) and with eEF1A•GDP (PDB ID, 5LZS) (**E**).

eEF2 binds to the hibernating ribosomes or 60S subunits similarly to that in translocation complexes, in which the GTPase domain is placed next to the SRL (Figure 5). The binding of eEF2/EF-G to pre-translocation ribosomes requires a GTP-bound conformation (e.g. GTP, non-hydrolyzable GTP analogs; Djumagulov et al., 2021; Taylor et al., 2007, or GDP+Pi; Carbone et al., 2021), as the ordered GTPase switch loops bridge the large subunit with the rotated small subunit (Carbone et al., 2021). On translocating ribosomes, the release of P_i_ after GTP hydrolysis and subunit rotation is coupled with switch-loop disordering and translocase dissociation (Carbone et al., 2021). Remarkably, we find that hibernating 80S ribosomes, in both rotated and non-rotated conformations, feature eEF2 with GDP (Figure 5A), and that the eEF2 switch loops remain ordered on the rotated ribosome.

Our examination of the eEF2-containing cryo-EM maps revealed that the GTPase domain is held at the SRL near ribosomal protein uL14. The N-terminal tail of uL14 has been unmodeled in previous structures of mammalian ribosomes with GTPases, likely due to disorder. Indeed, in cryo-EM maps of ribosomes bound with eukaryotic elongation factor 1A (eEF1A) (Shao et al., 2016) or release factor 3 (eRF3) (Shao et al., 2016) harboring homologous GTPase centers, density for the first 10 residues of uL14 is absent or placed farther from the GTPase centers. By contrast, continuous density is evident in our maps of eEF2-bound ribosomes and indicates that the N-terminus is reaching within 5 Å of GDP in the GTPase center of eEF2 (Figure 5B). The switch-I (SW-I) helix of eEF2 (aa 55–66) contacts residues 4–7 of uL14 (Figure 5B). This interaction resembles that in recent structures of *G. gallus,* eEF2 bound to translocation-like ribosomes (Nurullina et al., 2024), although the path of the N-terminal tails of uL14 was modeled differently (Figure 4—figure supplement 1). Similarly, continuous density is observed for the N-terminal tail of uL14 in our 60S•eEF2 complexes, where domain IV also contacts helix 69 of 28S rRNA (Figure 4—figure supplement 2), suggesting additional stabilization of eEF2. In sum, our findings demonstrate that SERBP1 is not required to stabilize eEF2 in the absence of mRNA and tRNA. The N-terminus of uL14, which adopts different conformations in the presence of different translational GTPases, may be involved in stabilizing eEF2 on the ribosome or isolated 60S subunits.

### Conclusions

We demonstrate that *in extracto* cryo-EM enables high-resolution visualization of ribosome complexes in the presence of cellular components. In comparison with canonical single-particle cryo-EM (usually involving purified macromolecules) and cellular cryo-EM/ET, this method offers the advantages of both methods (Table 1). First, the preservation of cytoplasmic components in lysates enables the identification of interactions among cytoplasmic macromolecules. Indeed, the identification of eEF2 and other novel factors in 60S and 80S complexes in our work contrasts with approaches in which ribosomes were purified prior to cryo-EM analyses. Furthermore, the comparison of fresh lysates prepared from normally treated MCF-7 cells and nutrient-deprived cells demonstrates the reduction of actively translating ribosomes and an increase in non-translating ribosomes and individual 60S subunits. Additional optimization of buffer conditions may be required to more accurately represent the translation states observed in cells, as ionic conditions are known to affect the conformation of the ribosomes (e.g. rotated/non-rotated) and binding of protein factors (Fedry et al., 2024; Choi and Puglisi, 2017; Spirin, 2009; Korostelev et al., 2008). For cells or samples with lower abundances of ribosomes or other macromolecules/complexes of interest, a lysate concentration step or collection of a larger dataset may be considered. Nevertheless, our initial analyses demonstrate the expected changes in the translational machinery, consistent with translational repression during nutrient deprivation in cells (Martínez-Pastor and Estruch, 1996; Ashe et al., 2000; Yamamoto and Izawa, 2013; Dever et al., 1992).

**Table 1.** Comparison of *in extract*o cryogenic electron microscopy (cryo-EM) with traditional *in vitro* and *in situ* cryogenic electron tomography (cryo-ET) approaches to structural biology.

Cryo-EM method *Features*	*In vitro* (traditional single particle)	*In extracto* (this work)	*In situ* (e.g. cryo-ET)
*Interactions with cellular components*	– *	+/–	+
*Purified macromolecules*	+	+/– (can be added)	–
*Time-resolved cryo-EM*	+	+	–
*Fast sample and grid preparation, and data collection to enable near-atomic resolution*	+ (hours to an overnight session)	+ (1–3 overnight sessions)	– (days/weeks)
*Efficient particle identification (low # of false positives*)	+	+	+/–

*+ Means straightforward, – difficult/impossible, +/– possible.

*In extracto* cryo-EM is a flexible method allowing to add purified components, such as an mRNA reporter (Figure 3), to lysates at different concentrations and time points, enabling equilibrium and time-resolved cryo-EM studies resembling *in vitro* single-particle cryo-EM (Carbone et al., 2021; Fu et al., 2019; Loveland et al., 2024). Although purified components could also be added to cells, their intracellular concentrations and time course are difficult to control and measure, making conditions in *in situ* cryo-EM more challenging to control than in *in extracto* cryo-EM. While this manuscript was in preparation, canonical single-particle cryo-EM analyses of bacterial lysates were reported, revealing numerous 70S ribosome structures (May et al., 2026). Similarly to mammalian lysates, bacterial systems are widely used with different reporters and translation factor combinations (Flügel et al., 2024; Kosaka et al., 2025), further underscoring the flexibility and broad applicability of *in extracto* cryo-EM.

Finally, we demonstrate that fast grid preparation with lysates (~10 min) enables data collection similar to or even faster than traditional single-particle cryo-EM, which requires sample purification. The resulting sample layers are thicker than those of purified macromolecules, necessitating the use of template matching, such as 2DTM and GisSPA (Cheng et al., 2023), which are slower than conventional particle picking methods implemented in most cryo-EM software packages. Yet, since no FIB milling of cellular samples is necessary for lysates, the overall throughput, including sample preparation and fast GPU-accelerated data processing, is superior to *in situ* cryo-ET or cryo-EM. Furthermore, although 2DTM may be computationally more expensive than traditional template-based particle picking employed with *in situ* samples, the latter is often subject to a large number of false positives (Pyle and Zanetti, 2021) that have to be removed in subsequent image-processing steps.

In this work, we use cryo-EM to grant a unique glimpse into the translation system that has been used in hundreds of studies in the past five decades (Koch et al., 1975; Lodish, 1973). In comparison with freshly made lysates from MCF-7 cells featuring ~55–70% ribosomes bound to an mRNA (Figure 2), the translationally active commercial nuclease-treated RRL contains a much smaller ~12% fraction of elongating ribosomes following the translation of an added mRNA construct. These findings are consistent with previous estimates of mRNA-bound ribosomes (Rifo et al., 2007) and likely indicate the expedient exhaustion of the added resources, degradation of mRNA, and/or other time-sensitive aspects of the cell-free lysate.

Nevertheless, thanks to the large fraction of non-translating ribosomes in RRL, our analyses have substantially expanded the understanding of the translational hibernation repertoire. We find that eEF2 is bound to the majority of hibernating ribosomes, highlighting that eEF2 functions beyond its canonical role of the translocase. The binding of eEF2 to hibernating 80S and 60S can occur in the absence of the previously identified abundant hibernation factor SERBP1, underscoring that the stabilization by SERBP1 is not required for this binding. Instead, eEF2 appears to be stabilized predominantly by the interactions with the SRL region on the large subunit. These interactions feature the N-terminus of uL14 approaching the GDP molecule in the eEF2 active site. The conformation of the N-terminal uL14 tail differs from those in eEF1- and eRF3-bound complexes, suggesting that the tail may contribute to stabilizing eEF2.

We also find that LARP1 is an abundant hibernation factor occupying the mRNA tunnel in 80S ribosomes. As discussed above, the simultaneous recruitment of LARP1 with IFRD2 or CCDC124, two other translational repressors, may serve to both protect all ribosome active centers and enhance their translational repression potential. Furthermore, LARP1 may co-localize TOP mRNAs with hibernating ribosomes to expediently resume translation of ribosomal proteins when stress subsides.

**Figure 6.**
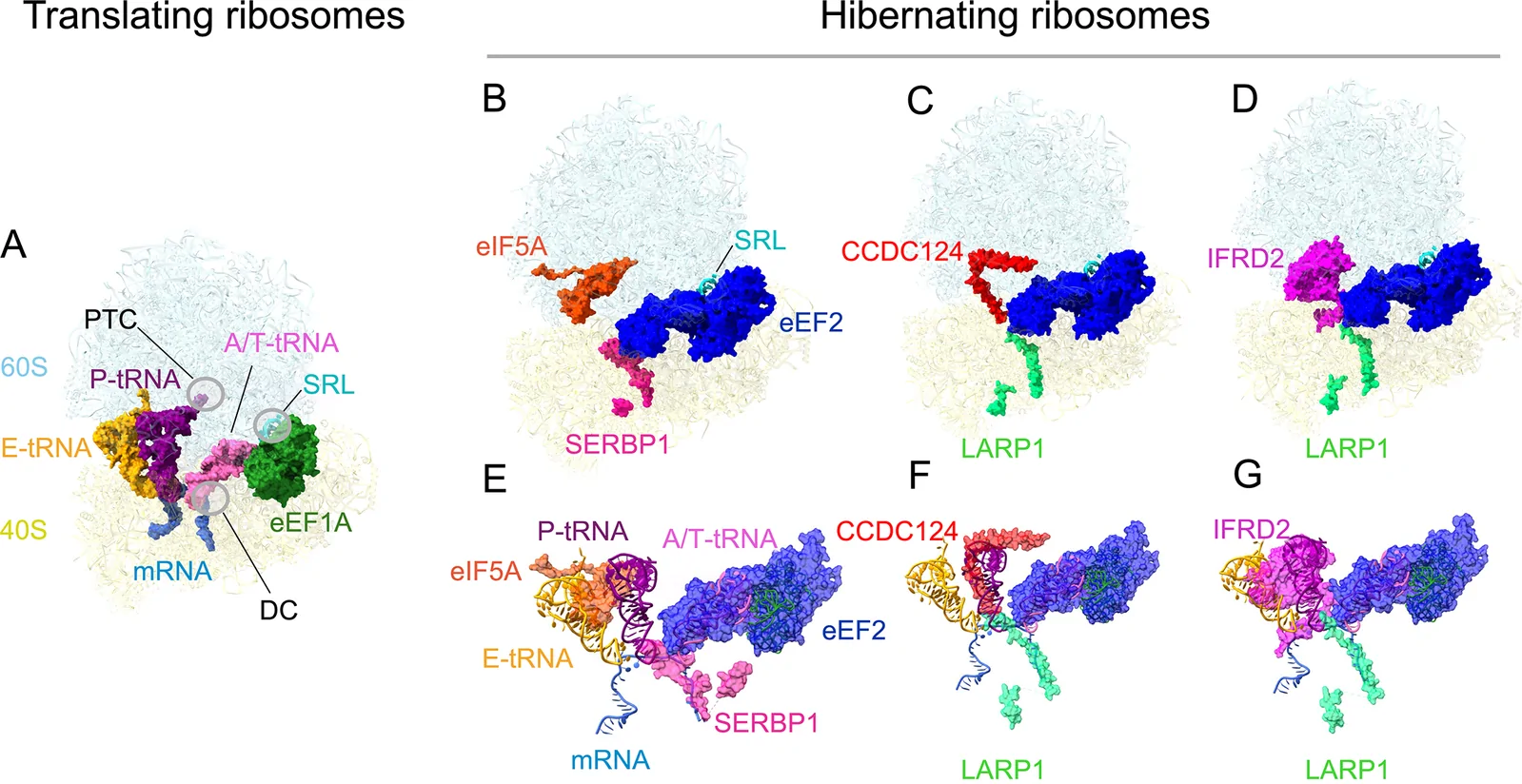
Binding sites of hibernation factors overlap with ribosomal functional centers. (**A–D**) Comparison of a translating ribosome (PDB 5LZS and mRNA from PDB 4V6F Jenner et al., 2010; panel A) with hibernating ribosomes identified in this work. (**E–G**) Superposition of translating and hibernating ribosomes illustrates that the key ribosomal functional centers are all shielded by hibernation factors (eEF1A, A/T-tRNA, P-tRNA, and E-tRNA are from PDB 5LZS and mRNA from PDB 4V6F). Panel E shows the view from panels B–D rotated by 36°.

In summary, although the composition of hibernation factors differs among hibernating ribosomes, their binding to the SRL (eEF2), mRNA tunnel and DC (SERBP1 or LARP1), P site and peptidyl-transferase center (eIF5A, CCDC124, or IFRD2) make these sites inaccessible for nucleolytic or other enzymes (Figure 6). These strategic positions are consistent with the proposed function of hibernating ribosome complexes being kept ‘in reserve’ to enter the pool of translating ribosomes when necessary (Usachev et al., 2020; Gohara and Yap, 2018). Together, our findings underscore the opportunities to uncover novel cellular interactions using *in extracto* cryo-EM.

## Methods

### Optimization of 2DTM in RRL data

To study angiogenin interactions with translating ribosomes, we collected a test cryo-EM dataset of translationally competent commercial lysate supplemented with recombinant angiogenin as described (Loveland et al., 2024). The sample contained 33% RRL (without nuclease treatment; Green Hectares), 1 µM angiogenin, and 2.5 ng/µl nanoluciferase mRNA and was applied to grids and vitrified using standard single-particle cryo-EM conditions (Loveland et al., 2024). During grid screening, we readily distinguished ribosomes in thin ice areas of the grid and performed overnight collection yielding 10,084 micrographs (Loveland et al., 2024). Standard single-particle cryo-EM pipelines, however, either failed to yield high-resolution reconstructions or discarded most of the particle data (Supplementary file 1A). We first utilized our standard pipeline aligning movies in IMOD (Li et al., 2013), importing micrographs into *cis*TEM for contrast transfer function (CTF) estimation and particle picking (272,478 particles), and searching alignment angles at low resolution (30–60 Å) against a 25 Å low-pass-filtered rabbit ribosome map (EMDB 4729 Shanmuganathan et al., 2019) in Frealign 9.11 (Lyumkis et al., 2013). The reconstructions displayed low resolution (>30 Å) and were dominated by a preferred orientation. Limiting the micrographs to ones where CTF Thon rings could be fitted to better than 3.2 Å resolution (600 micrographs with 7915 particles) and manual particle picking (removing areas of thick carbon or crystalline ice, recentering some picks) were required to accumulate a 6126-particle stack, yielding a reconstruction at 3.1 Å resolution (FSC 0.143). The cryo-EM map was further classified into six or eight classes, yielding four types of classes: 80S+angiogenin+strong ternary complex (80S+Ang+strong TC), 80S+angiogenin+weak ternary complex (80S+Ang+weak TC), hibernating 80S with eEF2, and 60S at 3.4–4.2 Å resolution. We also tried to use a Relion v3 (Zivanov et al., 2018) pipeline with CTF correction in GCTF, particle picking using the Laplacian-of-Gaussian picker (140,721 particles), with two rounds of sorting and eight rounds of 2D classification to remove ice contaminants and particles too small to be ribosomes, then aligning picked particles to a reference derived from EMD-4729, yielding an initial reconstruction at lower than 6.64 Å resolution (4× binned particle stack) from 52,580 particles. Two rounds of 3D classification yielded the same three classes as the *cis*TEM pipeline and at similar resolutions (up to 3.2 Å) from ~16,480 particles. Thus, despite imaging in cell lysates, with manual particle picking, we could distinguish and align some ribosome particles and find a novel, high-resolution state (80S+Ang+TC).

Inspection of the micrographs suggested that many ribosome particles were missed by the manual picking process. We therefore employed particle picking with 2DTM (Lucas et al., 2021). We started with a limited particle stack of the 1600 best micrographs from a total of 10,084 micrograph total and picked with a high-resolution template prepared from the 60S of the 80S•poly(GR) complex (PDB:7TOR; Loveland et al., 2022). The coordinates corresponding to the 60S subunit were saved as a separate PDB and converted into a .mrc map with the e2pdb2mrc.py program from EMAN2 (Tang et al., 2007) using a super-sampled pixel size of 0.415 Å, box size of 1024, and specifying that coordinates should be centered. The map was 2× binned using resample.exe from *cis*TEM (Lucas et al., 2021) and B-factor filtered using bfactor.exe (available at https://grigoriefflab.umassmed.edu/bfactor), applying a B-factor of 80 Å^2^ as described previously (Lucas et al., 2021). Particle picking with 2DTM in *cis*TEM yielded a stack of 37,859 particles, of which >95% belonged to high-resolution classes. 3D classification of this larger stack revealed states that were not previously separated in the *cis*TEM/Frealign v9.11 nor Relion 3 pipelines.

We optimized high-resolution template matching procedures for faster performance and maximum particle number with high signal/noise ratio. To this end, we performed a grid search for out-of-plane angles, in-plane angles, and defocus (z direction) search parameters on a limited set of micrographs. We found that increasing default values of out-of-plane angles from 2.5 to 3.5 and in-plane search angles from 1.5 to 2.5, omitting the defocus search, and resampling the micrographs and the search template to a 1.5 Å/pixel optimally speeds up the procedure without substantially sacrificing the number of picked particles. Using these parameters, we picked the full set of micrographs, excluding off-target grid locations (black images, images with broken ice, images on thick carbon), with the 60S template yielding 88,488 particles. The new dataset was rich in substates, including collided ribosomes in the translationally competent 80S•eEF2 and different positions or identities of ternary complexes in 80S•Ang•TC (see Supplementary file 1A). Thus, high-resolution template matching identifies ribosome particles in micrographs from cell lysates and reveals an abundance of novel states for analysis as compared to traditional single-molecule processing from select micrographs.

We found it beneficial to collect data from grid regions with thicker ice, where the population of ribosomes is higher than in thinner-ice regions of the grid, which contrasts the strategies for conventional single-particle grid and data optimization. To this end, we optimized the use of defocus search for each dataset or a batch of micrographs. We first grouped micrographs based on their CTF fit resolutions and selected 10 micrographs representing the highest and lowest fit resolutions. Template matching was then performed with the defocus search enabled or disabled, and the number of picked particles for each micrograph was compared. When a meaningful difference (in some cases, twofold or more) was observed—commonly for thicker-ice micrographs—we enabled defocus search for the corresponding groups of images. This approach allows maximizing the dataset size to enable extensive classification and improved resolutions of the resulting reconstructions.

### Cell cultures

MCF-7 cells (obtained from Dr. Michael Green’s lab; Bhatnagar et al., 2014) were cultured in DMEM (Gibco) supplemented with 20% FBS (Gibco) and 10% of Penicillin-Streptomycin (Gibco). BSC-1 cells (ATCC) were cultured in DMEM (Invitrogen) supplemented with 10% HI-FBS, 2 mM GlutaMAX (Gibco), and 50 µg/ml of Penicillin-Streptomycin (Gibco). Cell culture identity has been authenticated for both cell lines, using STR profiling (ATCC), and the cell lines tested negative for mycoplasma contamination (mycoplasma PCR detection kit, Cat No. G238, Applied Biological Materials).

### Preparation of MCF-7 and BSC-1 cell extracts

Cell extracts were prepared from MCF-7 cells by using digitonin buffer extraction, as in previous studies, with modifications (Behrmann et al., 2015; Hirashima and Kaji, 1970; Stephens and Nicchitta, 2007). To prepare non-stressed cells, MCF-7 cells were seeded in 75 cm^2^ flasks, with the media refreshed every 24 hr and 6 hr before cell collection, for 48 hr. For cells with nutrition stress, the media was not changed within 48 hr. Four flasks of cells were used for lysate preparation with a total of 8 ml of semi-permeabilization buffer (25 mM HEPES pH 7.2, 110 mM KOAc, 15 mM Mg(OAc)_2_, 1 mM dithiothreitol [DTT], 0.015% digitonin, 2× protease inhibitor [Roche], 40 U/ml RNase-In [Promega], 1 mM EGTA). Cells were washed twice with ice-cold PBS buffer (Gibco) and then 1 ml of semi-permeabilization buffer was added to each flask. After a 5 min incubation with the semi-permeabilization buffer, the cytosol-containing mixture was collected. This step was repeated to maximize sample collection (all this procedure was done in the cold room at 4°C). 8 ml of sample were transferred to 30 kDa MWCO Amicon filters and concentrated until the RNA concentration (A_260_; NanoDrop One Spectrophotometer, Thermo Fisher Scientific) exceeded 1000 ng/µl. Concentrated cell extract was directly added to the grids and vitrified as described below (Plunge freezing). To skip the concentration step, 200 μl of semi-permeabilization buffer (instead of 1 ml) can be added to each flask and collected for direct application to a grid.

BSC-1 cells were seeded in a 75 cm^2^ flask and cultured to confluence. After being washed twice with pre-warmed PBS, the cells were detached with Trypsin-EDTA (Gibco) and collected by centrifugation at 300×*g* for 4 min. The cell pellet was resuspended in 100 μl of semi-permeabilization buffer (25 mM HEPES, 110 mM KOAc, 15 mM Mg(OAc)_2_, 1 mM DTT, 0.015% digitonin, 2× protease inhibitor cocktail [Roche], 40 U/ml RNaseIn [Promega], 1 mM EGTA) at 4°C for 5 min. Following centrifugation at 1000×*g* for 5 min, the supernatant was collected for use in grid preparation (see below). RNA concentration was quantified prior to grid preparation as a quality control measure, with no dilution of the extract performed.

### Preparation of RRL samples for cryo-EM

Commercial micrococcal-nuclease-treated RRLs (Promega; L4960) were supplemented with the following buffers and reagents to reach the final concentration of 50% RRL: 30 mM HEPES-KOH, pH 7.5, 50 mM KOAc, 1 mM Mg(OAc)_2_, 0.2 mM rATP, 0.2 mM GTP, 0.02 mM amino acid mix minus Met (Promega), 0.02 mM amino acid mix minus Cys (Promega), and 5 mM DTT added with MilliQ-Water.

For grid preparation, two samples of RRL were prepared: (1) with mRNA encoding NanoLuciferase (see below) and (2) without mRNA. After thawing, 50% RRL (final concentration, prepared according to vendor instructions) was incubated for 5 min at 4°C. Then, a solution of NLuc mRNA (final concentration 25 ng/µl or ~100 nM, in water) or an equivalent volume of water was added and incubated for 10 min at 30°C. The mixture was directly applied to the grids and vitrified (see Plunge freezing).

### Preparation of *in vitro* transcribed NanoLuciferase mRNAs

A plasmid carrying the coding sequence for NanoLuciferase flanked by the 5′- and 3′-UTRs of rabbit *HBB2* was synthesized by Azenta (vector: pUC-GW-Kan), as designed and described (Susorov et al., 2020). DNA templates for *in vitro* transcription were PCR-amplified from the plasmid using Phusion High-Fidelity DNA Polymerase (NEB; M0530L) and primers (Integrated DNA Technologies) containing the T7 promoter (5′-TTTTTTAATACGACTCACTATAGGGAGAACACTTGCTTTTGACACAACTGTG-3′) and a 30-nt poly-A-tail (5′-TTTTTTTTTTTTTTTTTTTTTTTTTTTTTTGCAATGAAAATAAATTTCCTTTATTAGCC-3′). After PCR, DNA templates were purified by phenol/chloroform extraction and dissolved in nuclease-free Milli-Q water. DNA concentrations were measured using a NanoDrop One Spectrophotometer (Thermo Fisher Scientific). The presence of a single band as DNA template was confirmed by agarose gel electrophoresis (1% [wt/vol] agarose in TAE buffer). *In vitro* transcription reactions were carried out using 4 μg of purified DNA templates and purified recombinant T7 polymerase in transcription buffer (166 mM HEPES-KOH, pH 7.5; 20 mM MgCl_2_; 40 mM DTT, 2 mM spermidine, 25 mM each of ATP, GTP, CTP, and UTP; and 40 U/μl RNase Inhibitor [NEB; M0314S]) in an 80 μl reaction. After incubation at 37°C for 3.5 hr, magnesium pyrophosphate precipitate was removed by centrifugation (14,000×*g*, 5 min), and mRNA was precipitated from the supernatant by adding LiCl (2.5 M final concentration) and incubating at –80°C overnight. The next day, mRNA was pelleted by centrifugation (21,300×*g*, 15 min at 4°C), washed with cold 80% ethanol, and pelleted again. This washing step was repeated three times. After discarding the supernatant, the mRNA pellet was air-dried and dissolved in nuclease-free Milli-Q water. To attach a 5′ cap, capping reactions were performed using the Vaccinia Capping System (NEB; M2080S) following the protocol. The 5′ capped mRNA was then purified by LiCl precipitation as described above and dissolved in nuclease-free Milli-Q water. mRNA concentration was determined from A_260_ absorbance using a NanoDrop One Spectrophotometer (Thermo Fisher Scientific). The size and integrity of the *in vitro* transcribed mRNA were examined by denaturing agarose gel electrophoresis (1% [wt/vol] agarose in MOPS buffer with 1.11% [vol/vol] formaldehyde) alongside an ssRNA ladder (NEB; N0362S). The stock solution was stored at −80°C.

### *In vitro* translation in RRLs

*In vitro* translation was performed using a commercial micrococcal-nuclease-treated RRL (Promega; L4960) with modifications as described below. Translation reactions were carried out in the presence of 50% RRL, 30 mM HEPES-KOH (pH 7.5), 50 mM KOAc, 1.0 mM Mg(OAc)_2_, 0.2 mM ATP, 0.2 mM GTP, 0.04 mM of 20 amino acids (Promega), 5 mM DTT, and 1% furimazine NanoLuciferase substrate (Promega; N113A). To initiate translation, 11 µl of reactions were preincubated at 30°C for 3 min before adding *in vitro* transcribed mRNA encoding Nanoluciferase (100 nM final concentration) to a final reaction volume of 12 µl. Translation kinetics were measured over time by recording NanoLuciferase luminescence using an Infinite m1000 pro microplate reader (Tecan) at 30°C for 15 min.

### Plunge freezing

For MCF-7 and RRL lysates, Quantifoil R2/1 holey-carbon grids coated with a thin layer of carbon (Electron Microscopy Service) were glow-discharged with 20 mA current with negative polarity for 30 s in a PELCO easiGlow glow discharge unit. The Vitrobot Mark IV (Thermo Fisher Scientific) was pre-equilibrated to 4°C and 100% relative humidity, and the blot force was set to zero. For all samples, 2.5 µl of lysate for each blotting was applied to the grid (with no waiting time), blotted two or three times for 3–7 s, force zero, and plunged into liquid-nitrogen-cooled liquid ethane. Grids were stored in liquid nitrogen.

For BSC-1 cells, Quantifoil gold (Au) mesh grids with a holey SiO_2_ film (R 2/2) were glow-discharged using an EMITECH K100X system, applying a negative coating current of 25 mA for 45 s. Following treatment, the grids were blotted from the reverse side and rapidly plunged into liquid ethane at –184°C using a Leica EM GP plunger (Leica Microsystems) maintained at 15°C with 85% relative humidity. Blotting times were set to 8 s. The vitrified grids were subsequently stored in liquid nitrogen within sealed boxes until further processing.

### Cryo-EM data collection and analysis

All data were collected at the UMass Chan Medical School Cryo-EM Facility. Data for all samples except for starved MCF-7 cell lysates were acquired using a Krios electron microscope (Thermo Fisher Scientific) operating at 300 kV, equipped with a Gatan Image Filter (slit width, 20 eV) and a K3 direct electron detector (Gatan). Data from starved MCF-7 lysates were collected on the Talos, 200 kV. Data collection was automated with SerialEM (Mastronarde, 2005) using beam-image shift to collect multiple videos (e.g. five videos per hole at four holes) at each stage position, and targeting 0.7–μm underfocus.

The starved MCF-7 cells dataset contained 10,382movies collected with a total exposure of 40 e^−^/Å^−2^ and yielded 88,560 particles. For MCF-7 cells with no stress, the dataset comprised 10,022 movies with an exposure of 46 e^−^/Å^−2^ and yielded 89,484 particles. The movie frames were aligned during data collection using IMOD (Kremer et al., 1996) to decompress frames, apply the gain reference, and correct for image drift and particle damage. 2DTM was performed on parallelized Nvidia GPU processors (RTX A5000 and A6000), with the match_template program (Lucas et al., 2021) implemented in *cis*TEM on motion-corrected images (pixel size 0.83 Å for non-starved and 0.87 Å for starved cells), using an in-plane angular step of 2.5° and an out-of-plane step of 3.5°, with the defocus search turned on for micrographs with thicker ice. To generate the template, eIF6 was removed from PDB 7OW7, and the remaining 60S subunit was converted to a density map using EMAN2 (Tang et al., 2007) with the box size matching the stack, and the B-factor of 80 Å² was applied using bfactor.exe. Template-matched x, y coordinates, Euler angles, and defocus values from CTFFIND5 (Elferich et al., 2024) were extracted using the MT package in the *cisTEM* GUI, generating a .star file. The MT package was used to import the .star file, and the *cis*TEM refine package was utilized to prepare the particle stack with a box size of 608×608×608 pixels. The refine package stacks were exported into the Frealign format for maximum-likelihood classifications (Figure 2—figure supplements 2 and 3). Initial 3D reconstructions were generated using the Generate 3D tool in *cis*TEM. Further refinement of particle alignments included one cycle of x, y shifts, followed by a cycle of Euler angles, and another cycle of defocus and beam tilt refinement, all performed manually using the *cis*TEM GUI.

Four datasets were collected from RRL samples: two datasets from one grid prepared with mRNA, and two datasets from one grid without mRNA. The first two datasets comprised 13,127 and 14,697 movies (26 frames, with an exposure of 1.515 e⁻ Å⁻² per frame, resulting in a total exposure of 39.39 e⁻ Å⁻² per sample or exposure of 1.538 e⁻ Å⁻² per frame, resulting in a total exposure of 39.35 e⁻ Å⁻² per sample, respectively). The data were combined to comprise 27,924 movies to be processed with 2DTM. The datasets for RRL samples without mRNA contained 29,855 movies (dataset A: 26 frames, with an exposure of 1.5167 e⁻ Å⁻² per frame, resulting in a total exposure of 39.4338 e⁻ Å⁻² per sample) and 44,983 movies (dataset B: 22 frames, with an exposure of 1.3745 e⁻ Å⁻² per frame, resulting in a total exposure of 30.238 e⁻ Å⁻² per sample). All data collections targeted 0.7–2 μm underfocus. 2DTM was performed with the match_template program (Lucas et al., 2021) implemented in *cis*TEM on 2× binned images (pixel size 1.66 Å). To generate the template, eIF6 was removed from PDB 7OW7, and the remaining 60S subunit was converted to a density map using EMAN2 (Tang et al., 2007) with the box size matching the stack, and the B-factor of 200 Å^2^ was applied using bfactor.exe. The dataset was divided into blocks of 10,000 images (for each data collection) for 2DTM searches, yielding 209,874 particles from 27,924 movies of RRL with mRNA, 356,530 (dataset A) and 512,697 (dataset B) identified targets using an in-plane angular step of 3.5° and an out-of-plane step of 4°, without defocus search. Template-matched x, y coordinates, Euler angles, and defocus values from CTFFIND5 (Elferich et al., 2024) were extracted using the MT package in the *cis*TEM GUI, generating a .star file. These .star files were subsequently merged with an in-house Python script, and the 2× binned image paths were replaced with the corresponding 1× binned image paths. The MT package was used to import the updated .star file, and the *cis*TEM refine package was utilized to prepare the particle stack with a box size of 800×800×800 pixels. The refine package stack was exported into a Frealign v9.11 (Lyumkis et al., 2013) format for 3D classification (Figure 3—figure supplement 2).

Initial 3D reconstructions for the parent maps were generated using the Generate 3D tool in *cis*TEM. Further refinement of particle alignments included one cycle of x, y, followed by a cycle of Euler angles and another cycle of defocus and beam tilt refinement, all performed manually using the *cis*TEM GUI.

Data from BSC-1 samples were acquired using a Krios electron microscope (Thermo Fisher Scientific) operating at 300 kV, equipped with a Gatan Image Filter (slit width, 20 eV) and a K3 direct electron detector (Gatan), with a target defocus range of 1.0–1.5 μm. Automated data collection was conducted using SerialEM (Mastronarde, 2005), employing beam-image shift to acquire multiple movies (five per hole across nine holes) at each stage position. Zero-loss peak refinement was performed every 90 min at a unique location to avoid dark areas. The dataset from BSC-1 cells consisted of 24,550 movies, each containing 30 frames, with an exposure of 1.02 e⁻ Å^–2^ per frame, resulting in a total exposure of 30.5 e⁻ Å^–2^ per sample.

MotionCor2 (Zheng et al., 2017) was used for motion correction, gain reference application, exposure weighting, and binning of super-resolution pixels by factors of 2 and 4 to yield final pixel sizes of 0.83 Å and 1.66 Å, respectively. CTF estimation was performed using CTFFIND5 (Elferich et al., 2024) through the *cis*TEM graphical user interface (GUI) (Grant et al., 2018). For 2DTM, 3D templates were generated from a trimmed 5LZV PDB model containing only the 60S ribosomal subunit, using the simulate program (Himes and Grigorieff, 2021) from *cis*TEM to produce a 3D volume. A pixel size of 1.66 Å and a linear scaling factor of PDB B-factor of 1 were applied.

2DTM was performed with the match_template program (Lucas et al., 2021) implemented in *cis*TEM on 4× binned images (pixel size 1.66 Å). The dataset was divided into blocks of 2000 images for 2DTM searches, yielding 525,827 identified targets using an in-plane angular step of 1.5° and an out-of-plane step of 2.5°, without defocus search. Template-matched x, y coordinates, Euler angles, and defocus values from CTFFIND5 were extracted using the MT package in the *cis*TEM GUI, generating a .star file. These .star files were merged using an in-house Python script, and the 4× binned image paths were replaced with the corresponding 2× binned image paths. The MT package was used to import the updated .star file, and the *cis*TEM refine package was utilized to prepare the particle stack with a box size of 560×560×560 pixels.

Initial 3D reconstructions were generated using the Generate 3D tool in *cis*TEM. Further refinement of particle alignments included one cycle of x, y, followed by a cycle of Euler angles and another cycle of defocus and beam tilt refinement, all performed manually using the *cis*TEM GUI. The final alignment parameters and defocus values were exported in Frealign format.

### Cryo-EM data classification

#### MCF-7 lysates

Particle classifications were performed in Frealign v9.11 (Lyumkis et al., 2013). For both MCF-7 datasets, the box size was 608×608×608 pixels. To speed up processing, binned image stacks (e.g. 8×, 4×, or 2×) were prepared using resample.exe, part of the Frealign v9.11 distribution. Focused 3D maximum-likelihood classification into 24 classes (using the high-resolution limit of 16 Å and the 8× binned stack, 100–150 rounds) with an 80 Å focus mask covering the A site and the GTPase-activating center of the ribosome (x, y, z=261.73, 312.71, 208.96) resolved ribosomes with different occupancies of translation factors (Figure 2—figure supplements 2 and 3).

Classes were merged using merge_classes.exe from Frealign v9.11, applying a class occupancy threshold of 0.50 and a score of 0, followed by un-binning of particles using Frealign v9.11. Additional subclassifications were performed but did not result in additional high-resolution classes beyond those appearing in the initial classification.

#### RRL

The box size for RRL datasets was set to 800×800×800 pixels. To speed up processing, binned image stacks (e.g. 8×, 4×, or 2×) were prepared using resample.exe, part of the Frealign v9.11 distribution. Focused 3D maximum-likelihood classification into 40 classes (using the high-resolution limit of 16 Å and the 8× binned stack, 100 rounds) with an 80 Å focus mask covering the A site and the GTPase-activating center of the ribosome (x, y, z=309.98, 345.63, 232.03) resolved different ribosome states (Figure 3—figure supplement 2).

For RRL with mRNA, initial classification of 209,874 particles yielded two low-resolution (junk) classes with 13,791 particles (6.6%) that were removed from further classification. Out of nine classes corresponding to 60S subunits, two classes contain eEF2, and the remaining ones represent 60S without additional factors (Supplementary file 1E). 31 classes were 80S ribosomes: the predominant 19 classes (99,843 particles) contain eIF5A, SERBP1, and eEF2, and 3 classes (15,065 particles) represent the codon sampling states with eEF1A and A/T tRNA. One class with 4036 particles has tRNA in the A and P sites and additional density in the A site, which improved upon merging this class with particles from RRL without mRNA that featured nearly identical density. The additional A site density resembles DRG1 or DRG2 (Figure 3—figure supplement 3B). Six classes (22,093 particles) featured eEF2, although different density levels at factor-binding sites suggested that the cryo-EM maps are insufficiently separated. These six classes were merged (with criteria >50% occupancy and Score >0), resulting in a 17,529-particle stack and then subclassified to eight different classes with a mask on the P site. This classification resolved three predominant complexes: with eEF2, IFRD2, and LARP1 in the mRNA tunnel, with eEF2 and two tRNAs, and with eEF2, E-tRNA with SERBP1. For RRL without mRNA, classification of dataset A was performed with the same mask as in the above classification.

To achieve higher resolution, classes representing the same functional states in different RRL datasets were merged using merge_classes.exe from Frealign, applying a class occupancy threshold of 0.50 and the Score of 0, followed by 3D 1× binned reconstruction of particles using FrealignX or *cis*TEM. Initial 3D reconstructions were generated using the Generate 3D tool in *cis*TEM. Further refinement of particle alignments including one cycle of x, y, followed by a cycle of Euler angles and another cycle of defocus and beam tilt refinement, were performed using the *cis*TEM GUI.

For 80S with IFRD2 and LARP1, all representative classes from different datasets were merged in IMOD (71,194 particles). Using a 3D mask for eEF2 (eEF2 from PDB 6MTE was aligned to an 8× binned map containing eEF2 and then a 3D map was created based on the eEF2 model using molmap in ChimeraX Meng et al., 2023, then masked using vop onesmask #newmap onGrid #oldmap and saved as an mrc file), this stack (binned to match the 8× binned stack) was classified into two classes, resulting in two maps, both containing IFRD2 and LARP1, and either with or without eEF2. For 80S with eEF2 and CCDC124, particles were extracted with >50% occupancy and Score >0, and merged (27,910 particles). This stack was 4× binned and subclassified into six classes using a 45 Å mask on the P and E sites. For 60S with eEF2, classes with eEF2 from different datasets were merged in IMOD (38,579 particles) and classified using a 34 Å focus mask on eEF2 domain IV, resulting in 60S with open or compact eEF2.

#### BSC-1

An initial 3D maximum-likelihood classification (without particle alignment) was performed in Frealign v9.11 on the 4× binned stack, using 100 classes and 14 classification cycles (Lyumkis et al., 2013). The high-resolution limit for classification was set to 14 Å. Classes corresponding to equivalent states were then merged using an in-house Python script that selects particles with occupancy greater than 50%. This script preserves the file paths and generates a particle alignment file (.star) compatible with *cis*TEM (Lucas et al., 2021). The latter was used to generate the particle stack (.mrc) in *cis*TEM, which also allowed for refinement of particle alignments for each class in *cis*TEM. The script is available at https://github.com/GrigorieffLab/yafw (Elferich, 2026).

Subclassification was performed using a focus mask and 4× binned data (Figure 2—figure supplement 1), with Frealign v9.11. 3D reconstructions with the un-binned data (physical pixel size of 1.66 Å) were generated using either *cis*TEM or FrealignX.

### Model building and refinement

The cryo-EM structure of (GR)_20_-bound *Oryctolagus cuniculus* 80S ribosome (PDB: 7TOR; Loveland et al., 2022), omitting (GR)_20_ and P-tRNA, was used as the starting model for structure refinement into all maps (Supplementary file 1D). 60S, 40S head, 40S body were rigid body fitted into each cryo-EM map using ChimeraX (Meng et al., 2023) based on PDB 6MTD (Brown et al., 2018) as a reference structure for head-swiveled models. For eEF2 (P13639.EF-HUMAN), IFRD2 (Q12894-IFRD2-HUMAN), uL6 (P32969-RL9-HUMAN), uL14 (P62829.RL2 3_HUMAN), eL30 (P62890.RL30_RAT), uS19 (P62841.RS15_HUMAN), and eS28 (P62857.RS28-HUMAN), AlphaFold (Jumper et al., 2021) models were fitted into the density, using ChimeraX (Meng et al., 2023). The EBP1-ES27L structure was modeled from PDB: 7BHP (Bhaskar et al., 2021), LARP1 from PDB: 8XP2 (Saba et al., 2024), CCDC124 from PDB: 6Z6L (Wells et al., 2020), E-tRNA from PDB: 7OSM (Djumagulov et al., 2021), uL1 and eS12 from PDB: 6MTD. To model open and closed conformations of eEF2 in 60S complexes (Figure 3K–L), cryo-EM maps from both conformations were softened by applying the B-factor of 100 Å^2^ and rigid body fitted in Chimerax (Meng et al., 2023). After rigid-body fitting and manual modeling, structural models were refined using phenix.real_space_refine (Adams et al., 2011; Afonine et al., 2018; Liebschner et al., 2019), yielding structures with good stereochemistry and fits into corresponding cryo-EM maps (Supplementary file 1D).

## Data Availability

Structural models generated in this study have been deposited in the RCSB Protein Data Bank under the following accession codes: 11IQ (Rabbit 80S ribosome with eEF2, eIF5A and SERBP1), 11HG (Rabbit 80S ribosome with eEF2, CCDC124, and LARP1), 11JJ (Rabbit 80S ribosome with eEF2, IFRD2 and LARP1), 11KH (Rabbit 80S ribosome with IFRD2 and LARP1), 11HV (Rabbit 60S with eEF2, domain IV open), 11HE (Rabbit 60S with eEF2, domain IV closed). Cryo-EM maps used to generate these models have been deposited in the Electron Microscopy Data Bank under the following accession codes: EMD-75724 (Rabbit 80S ribosome with eEF2, eIF5A and SERBP1), EMD-75689 (Rabbit 80S ribosome with eEF2, CCDC124 and LARP1), EMD-75740 (Rabbit 80S ribosome with eEF2, IFRD2 and LARP1), EMD-75768 (Rabbit 80S ribosome with IFRD2 and LARP1), EMD-75704 (Rabbit 60S with eEF2, domain IV open), EMD-75687 (Rabbit 60S with eEF2, domain IV closed). Cryo-EM micrographs have been deposited in the Electron Microscopy Public Image Archive (EMPIAR) under the following accession codes: EMPIAR-13593 (merged rabbit reticulocyte lysate data), EMPIAR-13594 (MCF-7 cell lysate), EMPIAR-13595 (MCF-7-starved cell lysate), EMPIAR-13577 (BSC-1 cell lysate). The following datasets were generated: SerajZ ZottigX HuangC-Y LovelandAB DiggsS SholiE GrigorieffN KorostelevAA 2026Rabbit 80S ribosome with eEF2, eIF5a and SERBP1Worldwide Protein Data Bank10.2210/pdb11iq/pdbPMC1354130242689549 SerajZ ZottigX HuangC-Y LovelandAB DiggsS SholiE GrigorieffN KorostelevAA 2026Rabbit 80S ribosome with eEF2, CCDC124 and LARP1Worldwide Protein Data Bank10.2210/pdb11hg/pdbPMC1354130242689549 SerajZ ZottigX HuangC-Y LovelandAB DiggsS SholiE GrigorieffN KorostelevAA 2026Rabbit 80S ribosome with eEF2, IFRD2 and LARP1Worldwide Protein Data Bank10.2210/pdb11jj/pdbPMC1354130242689549 SerajZ ZottigX HuangC-Y LovelandAB DiggsS SholiE GrigorieffN KorostelevAA 2026Rabbit 80S ribosome with IFRD2 and LARP1Worldwide Protein Data Bank10.2210/pdb11kh/pdb SerajZ ZottigX HuangC-Y LovelandAB DiggsS SholiE GrigorieffN KorostelevAA 2026Rabbit 60S with eEF2, domain IV openWorldwide Protein Data Bank10.2210/pdb11hv/pdbPMC1354130242689549 SerajZ ZottigX HuangC-Y LovelandAB DiggsS SholiE GrigorieffN KorostelevAA 2026Rabbit 60S with eEF2, domain IV closedWorldwide Protein Data Bank10.2210/pdb11he/pdbPMC1354130242689549 SerajZ ZottigX HuangC-Y LovelandAB DiggsS SholiE GrigorieffN KorostelevAA 2026Rabbit 80S ribosome with eEF2, eIF5A and SERBP1Electron Microscopy Data BankEMD-7572410.7554/eLife.110114PMC1354130242689549 SerajZ ZottigX HuangC-Y LovelandAB DiggsS SholiE GrigorieffN KorostelevAA 2026Rabbit 80S ribosome with eEF2, CCDC124 and LARP1Electron Microscopy Data BankEMD-75689 SerajZ ZottigX HuangC-Y LovelandAB DiggsS SholiE GrigorieffN KorostelevAA 2026Rabbit 80S ribosome with eEF2, IFRD2 and LARP1Electron Microscopy Data BankEMD-7574010.7554/eLife.110114PMC1354130242689549 SerajZ ZottigX HuangC-Y LovelandAB DiggsS SholiE GrigorieffN KorostelevAA Electron Microscopy Data Bank2026Rabbit 80S with IFRD2 and LARP1, 40S head-swiveledEMD-7576810.7554/eLife.110114PMC1354130242689549 SerajZ ZottigX HuangC-Y LovelandAB DiggsS SholiE GrigorieffN KorostelevAA 2026Rabbit 60S with eEF2, domain IV openElectron Microscopy Data BankEMD-7570410.7554/eLife.110114PMC1354130242689549 SerajZ ZottigX HuangC-Y LovelandAB DiggsS SholiE GrigorieffN KorostelevAA 2026Rabbit 60S with eEF2, domain IV closedElectron Microscopy Data BankEMD-7568710.7554/eLife.110114PMC1354130242689549 SerajZ ZottigX HuangC-Y LovelandAB DiggsS SholiE GrigorieffN KorostelevAA Electron Microscopy Public Image Archive2026merged rabbit reticulocyte lysate dataEMPIAR-13593 SerajZ ZottigX HuangC-Y LovelandAB DiggsS SholiE GrigorieffN KorostelevAA Electron Microscopy Public Image Archive2026In extracto cryo-EM reveals eEF2 as a major hibernation factor on 60S and 80S particlesEMPIAR-1359410.7554/eLife.110114PMC1354130242689549 SerajZ ZottigX HuangC-Y LovelandAB DiggsS SholiE GrigorieffN KorostelevAA Electron Microscopy Public Image Archive2026MCF-7-starved cell lysateEMPIAR-13595 SerajZ ZottigX HuangC-Y LovelandAB DiggsS SholiE GrigorieffN KorostelevAA Electron Microscopy Public Image Archive2026In extracto cryo-EM reveals eEF2 as a major hibernation factor on 60S and 80S particlesEMPIAR-1357710.7554/eLife.110114PMC1354130242689549 The following previously published datasets were used: LovelandAB SvidritskiyE SusorovD LeeS ParkA ZvornicaninS DemoG GaoFB KorostelevAA 2022Mammalian 80S ribosome bound with the ALS/FTD-associated dipeptide repeat protein GR20Worldwide Protein Data Bank10.2210/pdb7tor/pdbPMC912001335589706 BrownA BairdMR YipMCJ MurrayJ ShaoS 2018Rabbit 80S ribosome with eEF2 and SERBP1 (unrotated state with 40S head swivel)Worldwide Protein Data Bank10.2210/pdb6mtd/pdb

## Additional files

Supplementary file 1. Tables A-E. (Table A) Particle picking strategies for the RRL+angiogenin dataset. (Table B) Ribosome states observed in lysates from different cell types. (Table C) Rotation of the 40S small ribosomal subunit head or body relative to non-rotated 80S ribosomes: with an accommodation-like eEF1A•aa-tRNA (PDB ID 5LZS; Shao et al., 2016) or with tRNAs in three classical sites (this work). Negative numbers report on the ‘underrotated’ subunits, i.e., rotated in the direction opposite to that measured from non-rotated (classical) to rotated (hybrid-state) pre-translocation ribosomes. (Table D) Cryo-EM data collection and model refinement statistics for 80S and 60S complexes from RRL. (Table E) Percentages of particles corresponding to 80S or 60S classes in RRL with NLuc mRNA and in RRL without NLuc mRNA.

MDAR checklist

## References

[bib1] Abeyrathne PD, Koh CS, Grant T, Grigorieff N, Korostelev AA (2016). Ensemble cryo-EM uncovers inchworm-like translocation of a viral IRES through the ribosome. eLife.

[bib2] Absmeier E, Chandrasekaran V, O’Reilly FJ, Stowell JAW, Rappsilber J, Passmore LA (2023). Specific recognition and ubiquitination of translating ribosomes by mammalian CCR4–NOT. Nature Structural & Molecular Biology.

[bib3] Adams PD, Afonine PV, Bunkóczi G, Chen VB, Echols N, Headd JJ, Hung LW, Jain S, Kapral GJ, Grosse Kunstleve RW, McCoy AJ, Moriarty NW, Oeffner RD, Read RJ, Richardson DC, Richardson JS, Terwilliger TC, Zwart PH (2011). The Phenix software for automated determination of macromolecular structures. Methods.

[bib4] Afonine PV, Poon BK, Read RJ, Sobolev OV, Terwilliger TC, Urzhumtsev A, Adams PD (2018). Real-space refinement in *PHENIX* for cryo-EM and crystallography. Acta Crystallographica Section D Structural Biology.

[bib5] Akers JF, LaScola M, Bothe A, Suh H, Jung C, Stolp ZD, Ghosh T, Yan LL, Wang Y, Macurak M, Devan A, McKinney MC, Grismer TS, Reyes AV, Ross EJ, Hu T, Xu S-L, Ban N, Kostova KK (2025). ZNF574 is a quality control factor for defective ribosome biogenesis intermediates. Molecular Cell.

[bib6] Ashe MP, De Long SK, Sachs AB (2000). Glucose depletion rapidly inhibits translation initiation in yeast. Molecular Biology of the Cell.

[bib7] Bai X-C, Fernandez IS, McMullan G, Scheres SHW (2013). Ribosome structures to near-atomic resolution from thirty thousand cryo-EM particles. eLife.

[bib8] Banari A, Samanta AK, Munke A, Laugks T, Bajt S, Grünewald K, Marlovits TC, Küpper J, Maia FRNC, Chapman HN, Oberthür D, Seuring C (2025). Advancing time-resolved structural biology: latest strategies in cryo-EM and X-ray crystallography. Nature Methods.

[bib9] Behrmann E, Loerke J, Budkevich TV, Yamamoto K, Schmidt A, Penczek PA, Vos MR, Bürger J, Mielke T, Scheerer P, Spahn CMT (2015). Structural snapshots of actively translating human ribosomes. Cell.

[bib10] Bhaskar V, Desogus J, Graff-Meyer A, Schenk AD, Cavadini S, Chao JA (2021). Dynamic association of human Ebp1 with the ribosome. RNA.

[bib11] Bhatnagar S, Gazin C, Chamberlain L, Ou J, Zhu X, Tushir JS, Virbasius CM, Lin L, Zhu LJ, Wajapeyee N, Green MR (2014). TRIM37 is a new histone H2A ubiquitin ligase and breast cancer oncoprotein. Nature.

[bib12] Bhattacharjee S, Feng X, Maji S, Dadhwal P, Zhang Z, Brown ZP, Frank J (2024). Time resolution in cryo-EM using a PDMS-based microfluidic chip assembly and its application to the study of HflX-mediated ribosome recycling. Cell.

[bib13] Brown A, Baird MR, Yip MC, Murray J, Shao S (2018). Structures of translationally inactive mammalian ribosomes. eLife.

[bib14] Campbell MG, Cheng A, Brilot AF, Moeller A, Lyumkis D, Veesler D, Pan J, Harrison SC, Potter CS, Carragher B, Grigorieff N (2012). Movies of ice-embedded particles enhance resolution in electron cryo-microscopy. Structure.

[bib15] Carbone CE, Loveland AB, Gamper HB, Hou YM, Demo G, Korostelev AA (2021). Time-resolved cryo-EM visualizes ribosomal translocation with EF-G and GTP. Nature Communications.

[bib16] Carter SD, Hampton CM, Langlois R, Melero R, Farino ZJ, Calderon MJ, Li W, Wallace CT, Tran NH, Grassucci RA, Siegmund SE, Pemberton J, Morgenstern TJ, Eisenman L, Aguilar JI, Greenberg NL, Levy ES, Yi E, Mitchell WG, Rice WJ, Wigge C, Pilli J, George EW, Aslanoglou D, Courel M, Freyberg RJ, Javitch JA, Wills ZP, Area-Gomez E, Shiva S, Bartolini F, Volchuk A, Murray SA, Aridor M, Fish KN, Walter P, Balla T, Fass D, Wolf SG, Watkins SC, Carazo JM, Jensen GJ, Frank J, Freyberg Z (2020). Ribosome-associated vesicles: a dynamic subcompartment of the endoplasmic reticulum in secretory cells. Science Advances.

[bib17] Cheng J, Liu T, You X, Zhang F, Sui SF, Wan X, Zhang X (2023). Determining protein structures in cellular lamella at pseudo-atomic resolution by GisSPA. Nature Communications.

[bib18] Choi J, Puglisi JD (2017). Three tRNAs on the ribosome slow translation elongation. PNAS.

[bib19] Chua EYD, Mendez JH, Rapp M, Ilca SL, Tan YZ, Maruthi K, Kuang H, Zimanyi CM, Cheng A, Eng ET, Noble AJ, Potter CS, Carragher B (2022). Better, faster, cheaper: recent advances in cryo-electron microscopy. Annual Review of Biochemistry.

[bib20] Cornish PV, Ermolenko DN, Noller HF, Ha T (2008). Spontaneous intersubunit rotation in single ribosomes. Molecular Cell.

[bib21] Dadhwal P (2024). Mammalian Translation Termination Intermediates Captured Using PDMS Microfluidics-Based Time-Resolved Cryo-EM (TRCEM).

[bib22] Daugeron M-C, Prouteau M, Lacroute F, Séraphin B (2011). The highly conserved eukaryotic DRG factors are required for efficient translation in a manner redundant with the putative RNA helicase Slh1. Nucleic Acids Research.

[bib23] Demo G, Gamper HB, Loveland AB, Masuda I, Carbone CE, Svidritskiy E, Hou Y-M, Korostelev AA (2021). Structural basis for +1 ribosomal frameshifting during EF-G-catalyzed translocation. Nature Communications.

[bib24] de Teresa-Trueba I, Goetz SK, Mattausch A, Stojanovska F, Zimmerli CE, Toro-Nahuelpan M, Cheng DWC, Tollervey F, Pape C, Beck M, Diz-Muñoz A, Kreshuk A, Mahamid J, Zaugg JB (2023). Convolutional networks for supervised mining of molecular patterns within cellular context. Nature Methods.

[bib25] Dever TE, Feng L, Wek RC, Cigan AM, Donahue TF, Hinnebusch AG (1992). Phosphorylation of initiation factor 2 alpha by protein kinase GCN2 mediates gene-specific translational control of GCN4 in yeast. Cell.

[bib26] Djumagulov M, Demeshkina N, Jenner L, Rozov A, Yusupov M, Yusupova G (2021). Accuracy mechanism of eukaryotic ribosome translocation. Nature.

[bib27] Donnelly N, Gorman AM, Gupta S, Samali A (2013). The eIF2α kinases: their structures and functions. Cellular and Molecular Life Sciences.

[bib28] Du M, Li X, Dong W, Zeng F (2024). Implication of Stm1 in the protection of eIF5A, eEF2 and tRNA through dormant ribosomes. Frontiers in Molecular Biosciences.

[bib29] Ekemezie CL, Melnikov SV (2024). Hibernating ribosomes as drug targets?. Frontiers in Microbiology.

[bib30] Elferich J, Kong L, Zottig X, Grigorieff N (2024). CTFFIND5 provides improved insight into quality, tilt, and thickness of TEM samples. eLife.

[bib31] Elferich J (2026). Github.

[bib32] Ermolenko DN, Noller HF (2011). mRNA translocation occurs during the second step of ribosomal intersubunit rotation. Nature Structural & Molecular Biology.

[bib33] Fan HY, Heerklotz H (2017). Digitonin does not flip across cholesterol-poor membranes. Journal of Colloid and Interface Science.

[bib34] Fedry J, Silva J, Vanevic M, Fronik S, Mechulam Y, Schmitt E, des Georges A, Faller WJ, Förster F (2024). Visualization of translation reorganization upon persistent ribosome collision stress in mammalian cells. Molecular Cell.

[bib35] Flis J, Holm M, Rundlet EJ, Loerke J, Hilal T, Dabrowski M, Bürger J, Mielke T, Blanchard SC, Spahn CMT, Budkevich TV (2018). tRNA translocation by the eukaryotic 80S ribosome and the impact of GTP hydrolysis. Cell Reports.

[bib36] Flügel T, Schacherl M, Unbehaun A, Schroeer B, Dabrowski M, Bürger J, Mielke T, Sprink T, Diebolder CA, Guillén Schlippe YV, Spahn CMT (2024). Transient disome complex formation in native polysomes during ongoing protein synthesis captured by cryo-EM. Nature Communications.

[bib37] Fonseca BD, Zakaria C, Jia J-J, Graber TE, Svitkin Y, Tahmasebi S, Healy D, Hoang H-D, Jensen JM, Diao IT, Lussier A, Dajadian C, Padmanabhan N, Wang W, Matta-Camacho E, Hearnden J, Smith EM, Tsukumo Y, Yanagiya A, Morita M, Petroulakis E, González JL, Hernández G, Alain T, Damgaard CK (2015). La-related Protein 1 (LARP1) represses Terminal Oligopyrimidine (TOP) mRNA translation downstream of mTOR Complex 1 (mTORC1). The Journal of Biological Chemistry.

[bib38] Fu Z, Kaledhonkar S, Borg A, Sun M, Chen B, Grassucci RA, Ehrenberg M, Frank J (2016). Key intermediates in ribosome recycling visualized by time-resolved cryoelectron microscopy. Structure.

[bib39] Fu Z, Indrisiunaite G, Kaledhonkar S, Shah B, Sun M, Chen B, Grassucci RA, Ehrenberg M, Frank J (2019). The structural basis for release factor activation during translation termination revealed by time-resolved cryogenic electron microscopy. Biophysical Journal.

[bib40] Gao YG, Selmer M, Dunham CM, Weixlbaumer A, Kelley AC, Ramakrishnan V (2009). The structure of the ribosome with elongation factor G trapped in the posttranslocational state. Science.

[bib41] Gemmer M, Chaillet ML, van Loenhout J, Cuevas Arenas R, Vismpas D, Gröllers-Mulderij M, Koh FA, Albanese P, Scheltema RA, Howes SC, Kotecha A, Fedry J, Förster F (2023). Visualization of translation and protein biogenesis at the ER membrane. Nature.

[bib42] Gohara DW, Yap MNF (2018). Survival of the drowsiest: the hibernating 100S ribosome in bacterial stress management. Current Genetics.

[bib43] Grant T, Rohou A, Grigorieff N (2018). *cis*TEM, user-friendly software for single-particle image processing. eLife.

[bib44] Greber BJ, Gerhardy S, Leitner A, Leibundgut M, Salem M, Boehringer D, Leulliot N, Aebersold R, Panse VG, Ban N (2016). Insertion of the biogenesis factor Rei1 probes the ribosomal tunnel during 60S maturation. Cell.

[bib45] Harding HP, Novoa I, Zhang Y, Zeng H, Wek R, Schapira M, Ron D (2000). Regulated translation initiation controls stress-induced gene expression in mammalian cells. Molecular Cell.

[bib46] Heydari S, Liu J (2025). High-throughput cryo-electron tomography enables multiscale visualization of the inner life of microbes. Current Opinion in Structural Biology.

[bib47] Himes B, Grigorieff N (2021). Cryo-TEM simulations of amorphous radiation-sensitive samples using multislice wave propagation. IUCrJ.

[bib48] Hirashima A, Kaji A (1970). Factor dependent breakdown of polysomes. Biochemical and Biophysical Research Communications.

[bib49] Hoffmann PC, Kreysing JP, Khusainov I, Tuijtel MW, Welsch S, Beck M (2022). Structures of the eukaryotic ribosome and its translational states *in situ*. Nature Communications.

[bib50] Hong S, Freeberg MA, Han T, Kamath A, Yao Y, Fukuda T, Suzuki T, Kim JK, Inoki K (2017). LARP1 functions as a molecular switch for mTORC1-mediated translation of an essential class of mRNAs. eLife.

[bib51] Jaako P, Faille A, Tan S, Wong CC, Escudero-Urquijo N, Castro-Hartmann P, Wright P, Hilcenko C, Adams DJ, Warren AJ (2022). eIF6 rebinding dynamically couples ribosome maturation and translation. Nature Communications.

[bib52] Jaskolowski M, Jomaa A, Gamerdinger M, Shrestha S, Leibundgut M, Deuerling E, Ban N (2023). Molecular basis of the TRAP complex function in ER protein biogenesis. Nature Structural & Molecular Biology.

[bib53] Jenner LB, Demeshkina N, Yusupova G, Yusupov M (2010). Structural aspects of messenger RNA reading frame maintenance by the ribosome. Nature Structural & Molecular Biology.

[bib54] Jumper J, Evans R, Pritzel A, Green T, Figurnov M, Ronneberger O, Tunyasuvunakool K, Bates R, Žídek A, Potapenko A, Bridgland A, Meyer C, Kohl SAA, Ballard AJ, Cowie A, Romera-Paredes B, Nikolov S, Jain R, Adler J, Back T, Petersen S, Reiman D, Clancy E, Zielinski M, Steinegger M, Pacholska M, Berghammer T, Bodenstein S, Silver D, Vinyals O, Senior AW, Kavukcuoglu K, Kohli P, Hassabis D (2021). Highly accurate protein structure prediction with AlphaFold. Nature.

[bib55] Kaledhonkar S, Fu Z, Caban K, Li W, Chen B, Sun M, Gonzalez RL, Frank J (2019). Late steps in bacterial translation initiation visualized using time-resolved cryo-EM. Nature.

[bib56] Kaul G, Pattan G, Rafeequi T (2011). Eukaryotic elongation factor-2 (eEF2): its regulation and peptide chain elongation. Cell Biochemistry and Function.

[bib57] Klebl DP, McMillan SN, Risi C, Forgacs E, Virok B, Atherton JL, Harris SA, Stofella M, Winkelmann DA, Sobott F, Galkin VE, Knight PJ, Muench SP, Scarff CA, White HD (2025). Swinging lever mechanism of myosin directly shown by time-resolved cryo-EM. Nature.

[bib58] Koch PA, Gardner FH, Gartrell JE, Carter JR (1975). Biogenesis of erythrocyte membrane proteins *in vitro* studies with rabbit reticulocytes. Biochimica et Biophysica Acta (BBA) - Biomembranes.

[bib59] Koli S, Shetty S (2024). Ribosomal dormancy at the nexus of ribosome homeostasis and protein synthesis. BioEssays.

[bib60] Korostelev A, Ermolenko DN, Noller HF (2008). Structural dynamics of the ribosome. Current Opinion in Chemical Biology.

[bib61] Korostelev AA (2022). The structural dynamics of translation. Annual Review of Biochemistry.

[bib62] Kosaka Y, Miyawaki Y, Mori M, Aburaya S, Nishizawa C, Chujo T, Niwa T, Miyazaki T, Sugita T, Fukuyama M, Taguchi H, Tomizawa K, Sugase K, Ueda M, Aoki W (2025). Autonomous ribosome biogenesis *in vitro*. Nature Communications.

[bib63] Kremer JR, Mastronarde DN, McIntosh JR (1996). Computer visualization of three-dimensional image data using IMOD. Journal of Structural Biology.

[bib64] Li X, Mooney P, Zheng S, Booth CR, Braunfeld MB, Gubbens S, Agard DA, Cheng Y (2013). Electron counting and beam-induced motion correction enable near-atomic-resolution single-particle cryo-EM. Nature Methods.

[bib65] Liao M, Cao E, Julius D, Cheng Y (2013). Structure of the TRPV1 ion channel determined by electron cryo-microscopy. Nature.

[bib66] Liebschner D, Afonine PV, Baker ML, Bunkóczi G, Chen VB, Croll TI, Hintze B, Hung LW, Jain S, McCoy AJ, Moriarty NW, Oeffner RD, Poon BK, Prisant MG, Read RJ, Richardson JS, Richardson DC, Sammito MD, Sobolev OV, Stockwell DH, Terwilliger TC, Urzhumtsev AG, Videau LL, Williams CJ, Adams PD (2019). Macromolecular structure determination using X-rays, neutrons and electrons: recent developments in Phenix. Acta Crystallographica. Section D, Structural Biology.

[bib67] Ling C, Ermolenko DN (2016). Structural insights into ribosome translocation. Wiley Interdisciplinary Reviews. RNA.

[bib68] Lipońska A, Yap MNF (2021). Hibernation-promoting factor sequesters *Staphylococcus aureus* ribosomes to antagonize RNase R-mediated nucleolytic degradation. mBio.

[bib69] Lodish HF (1973). Biosynthesis of reticulocyte membrane proteins by membrane-free polyribosomes. PNAS.

[bib70] Loveland AB, Demo G, Korostelev AA (2020). Cryo-EM of elongating ribosome with EF-Tu•GTP elucidates tRNA proofreading. Nature.

[bib71] Loveland AB, Svidritskiy E, Susorov D, Lee S, Park A, Zvornicanin S, Demo G, Gao F-B, Korostelev AA (2022). Ribosome inhibition by C9ORF72-ALS/FTD-associated poly-PR and poly-GR proteins revealed by cryo-EM. Nature Communications.

[bib72] Loveland AB, Koh CS, Ganesan R, Jacobson A, Korostelev AA (2024). Structural mechanism of angiogenin activation by the ribosome. Nature.

[bib73] Lucas BA, Himes BA, Xue L, Grant T, Mahamid J, Grigorieff N (2021). Locating macromolecular assemblies in cells by 2D template matching with cisTEM. eLife.

[bib74] Lyumkis D, Brilot AF, Theobald DL, Grigorieff N (2013). Likelihood-based classification of cryo-EM images using FREALIGN. Journal of Structural Biology.

[bib75] Martínez-Pastor MT, Estruch F (1996). Sudden depletion of carbon source blocks translation, but not transcription, in the yeast *Saccharomyces cerevisiae*. FEBS Letters.

[bib76] Mastronarde DN (2005). Automated electron microscope tomography using robust prediction of specimen movements. Journal of Structural Biology.

[bib77] May MB, Lopez-Perez GS, Davis JH (2026). Capturing ribosomal structures in cellular extracts with cryoPRISM: A purification-free cryoEM approach reveals novel structural states. PNAS.

[bib78] Meng EC, Goddard TD, Pettersen EF, Couch GS, Pearson ZJ, Morris JH, Ferrin TE (2023). UCSF ChimeraX: Tools for structure building and analysis. Protein Science.

[bib79] Miyamoto K, Yamashita T, Tsukiyama T, Kitamura N, Minami N, Yamada M, Imai H (2008). Reversible membrane permeabilization of mammalian cells treated with digitonin and its use for inducing nuclear reprogramming by Xenopus egg extracts. Cloning and Stem Cells.

[bib80] Nogales E, Mahamid J (2024). Bridging structural and cell biology with cryo-electron microscopy. Nature.

[bib81] Noller HF (2024). The ribosome comes to life. Cell.

[bib82] Nurullina L, Terrosu S, Myasnikov AG, Jenner LB, Yusupov M (2024). Cryo-EM structure of the inactive ribosome complex accumulated in chick embryo cells in cold-stress conditions. FEBS Letters.

[bib83] Pelham HR, Jackson RJ (1976). An efficient mRNA-dependent translation system from reticulocyte lysates. European Journal of Biochemistry.

[bib84] Pestova TV, Kolupaeva VG (2002). The roles of individual eukaryotic translation initiation factors in ribosomal scanning and initiation codon selection. Genes & Development.

[bib85] Pochopien AA, Beckert B, Kasvandik S, Berninghausen O, Beckmann R, Tenson T, Wilson DN (2021). Structure of Gcn1 bound to stalled and colliding 80S ribosomes. PNAS.

[bib86] Pyle E, Zanetti G (2021). Current data processing strategies for cryo-electron tomography and subtomogram averaging. The Biochemical Journal.

[bib87] Quade N, Boehringer D, Leibundgut M, van den Heuvel J, Ban N (2015). Cryo-EM structure of Hepatitis C virus IRES bound to the human ribosome at 3.9-Å resolution. Nature Communications.

[bib88] Ramrath DJF, Lancaster L, Sprink T, Mielke T, Loerke J, Noller HF, Spahn CMT (2013). Visualization of two transfer RNAs trapped in transit during elongation factor G-mediated translocation. PNAS.

[bib89] Rifo RS, Ricci EP, Decimo D, Moncorge O, Ohlmann T (2007). Back to basics: the untreated rabbit reticulocyte lysate as a competitive system to recapitulate cap/poly(A) synergy and the selective advantage of IRES-driven translation. Nucleic Acids Research.

[bib90] Rodnina MV, Wintermeyer W, Green R (2011). Ribosomes Structure, Function, and Dynamics.

[bib91] Saba JA, Huang Z, Schole KL, Ye X, Bhatt SD, Li Y, Timp W, Cheng J, Green R (2024). LARP1 binds ribosomes and TOP mRNAs in repressed complexes. The EMBO Journal.

[bib92] Salsi E, Farah E, Netter Z, Dann J, Ermolenko DN (2015). Movement of elongation factor G between compact and extended conformations. Journal of Molecular Biology.

[bib93] Scheres SHW (2012). RELION: implementation of a Bayesian approach to cryo-EM structure determination. Journal of Structural Biology.

[bib94] Schuller AP, Wu CCC, Dever TE, Buskirk AR, Green R (2017). eIF5A functions globally in translation elongation and termination. Molecular Cell.

[bib95] Shanmuganathan V, Schiller N, Magoulopoulou A, Cheng J, Braunger K, Cymer F, Berninghausen O, Beatrix B, Kohno K, von Heijne G, Beckmann R (2019). Structural and mutational analysis of the ribosome-arresting human XBP1u. eLife.

[bib96] Shao S, Murray J, Brown A, Taunton J, Ramakrishnan V, Hegde RS (2016). Decoding mammalian ribosome-mRNA states by translational GTPase complexes. Cell.

[bib97] Shedlovskiy D, Zinskie JA, Gardner E, Pestov DG, Shcherbik N (2017). Endonucleolytic cleavage in the expansion segment 7 of 25S rRNA is an early marker of low-level oxidative stress in yeast. The Journal of Biological Chemistry.

[bib98] Shetty S, Hofstetter J, Battaglioni S, Ritz D, Hall MN (2023). TORC1 phosphorylates and inhibits the ribosome preservation factor Stm1 to activate dormant ribosomes. The EMBO Journal.

[bib99] Shi X, Khade PK, Sanbonmatsu KY, Joseph S (2012). Functional role of the sarcin–ricin loop of the 23S rRNA in the elongation cycle of protein synthesis. Journal of Molecular Biology.

[bib100] Smith PR, Loerch S, Kunder N, Stanowick AD, Lou T-F, Campbell ZT (2021). Functionally distinct roles for eEF2K in the control of ribosome availability and p-body abundance. Nature Communications.

[bib101] Smith PR, Pandit SC, Loerch S, Campbell ZT (2022). The space between notes: emerging roles for translationally silent ribosomes. Trends in Biochemical Sciences.

[bib102] Spirin AS (2009). The ribosome as a conveying thermal ratchet machine. The Journal of Biological Chemistry.

[bib103] Squatrito M, Mancino M, Sala L, Draetta GF (2006). Ebp1 is a dsRNA-binding protein associated with ribosomes that modulates eIF2alpha phosphorylation. Biochemical and Biophysical Research Communications.

[bib104] Stephens SB, Nicchitta CV (2007). *In vitro* and tissue culture methods for analysis of translation initiation on the endoplasmic reticulum. Methods in Enzymology.

[bib105] Susorov D, Egri S, Korostelev AA (2020). Termi-Luc: a versatile assay to monitor full-protein release from ribosomes. RNA.

[bib106] Tang G, Peng L, Baldwin PR, Mann DS, Jiang W, Rees I, Ludtke SJ (2007). EMAN2: an extensible image processing suite for electron microscopy. Journal of Structural Biology.

[bib107] Taylor DJ, Nilsson J, Merrill AR, Andersen GR, Nissen P, Frank J (2007). Structures of modified eEF2 80S ribosome complexes reveal the role of GTP hydrolysis in translocation. The EMBO Journal.

[bib108] Thoms M, Buschauer R, Ameismeier M, Koepke L, Denk T, Hirschenberger M, Kratzat H, Hayn M, Mackens-Kiani T, Cheng J, Straub JH, Stürzel CM, Fröhlich T, Berninghausen O, Becker T, Kirchhoff F, Sparrer KMJ, Beckmann R (2020). Structural basis for translational shutdown and immune evasion by the Nsp1 protein of SARS-CoV-2. Science.

[bib109] Usachev KS, Yusupov MM, Validov ShZ (2020). Hibernation as a stage of ribosome functioning. Biochemistry.

[bib110] Voorhees RM, Ramakrishnan V (2013). Structural basis of the translational elongation cycle. Annual Review of Biochemistry.

[bib111] Wasserman MR, Alejo JL, Altman RB, Blanchard SC (2016). Multiperspective smFRET reveals rate-determining late intermediates of ribosomal translocation. Nature Structural & Molecular Biology.

[bib112] Weigel PH, Ray DA, Oka JA (1983). Quantitation of intracellular membrane-bound enzymes and receptors in digitonin-permeabilized cells. Analytical Biochemistry.

[bib113] Wells JN, Buschauer R, Mackens-Kiani T, Best K, Kratzat H, Berninghausen O, Becker T, Gilbert W, Cheng J, Beckmann R (2020). Structure and function of yeast Lso2 and human CCDC124 bound to hibernating ribosomes. PLOS Biology.

[bib114] Willi J, Küpfer P, Evéquoz D, Fernandez G, Katz A, Leumann C, Polacek N (2018). Oxidative stress damages rRNA inside the ribosome and differentially affects the catalytic center. Nucleic Acids Research.

[bib115] Wolin E, Guo JK, Blanco MR, Goronzy IN, Gorhe D, Dong W, Perez AA, Keskin A, Valenzuela E, Abdou AA, Urbinati CR, Kaufhold R, Rube HT, Brito Querido J, Guttman M, Jovanovic M (2025). SPIDR enables multiplexed mapping of RNA-protein interactions and uncovers a mechanism for selective translational suppression upon cell stress. Cell.

[bib116] Xing H, Taniguchi R, Khusainov I, Kreysing JP, Welsch S, Turoňová B, Beck M (2023). Translation dynamics in human cells visualized at high resolution reveal cancer drug action. Science.

[bib117] Xue L, Lenz S, Zimmermann-Kogadeeva M, Tegunov D, Cramer P, Bork P, Rappsilber J, Mahamid J (2022). Visualizing translation dynamics at atomic detail inside a bacterial cell. Nature.

[bib118] Yamamoto Y, Izawa S (2013). Adaptive response in stress granule formation and bulk translational repression upon a combined stress of mild heat shock and mild ethanol stress in yeast. Genes to Cells.

[bib119] Yamamoto H, Collier M, Loerke J, Ismer J, Schmidt A, Hilal T, Sprink T, Yamamoto K, Mielke T, Bürger J, Shaikh TR, Dabrowski M, Hildebrand PW, Scheerer P, Spahn CMT (2015). Molecular architecture of the ribosome-bound Hepatitis C Virus internal ribosomal entry site RNA. The EMBO Journal.

[bib120] Young LN, Villa E (2023). Bringing structure to cell biology with cryo-electron tomography. Annual Review of Biophysics.

[bib121] Zeng F, Li X, Pires-Alves M, Chen X, Hawk CW, Jin H (2021). Conserved heterodimeric GTPase Rbg1/Tma46 promotes efficient translation in eukaryotic cells. Cell Reports.

[bib122] Zhang Z, Wang W, Dong Z, Yang X, Liang F, Chen X, Wang C, Luo C, Zhang J, Wu X, Sun L, Chu J (2022). The trends of *in situ* focused ion beam technology: toward preparing transmission electron microscopy lamella and devices at the atomic scale. Advanced Electronic Materials.

[bib123] Zheng SQ, Palovcak E, Armache J-P, Verba KA, Cheng Y, Agard DA (2017). MotionCor2: anisotropic correction of beam-induced motion for improved cryo-electron microscopy. Nature Methods.

[bib124] Zheng W, Zhang Y, Wang J, Wang S, Chai P, Bailey EJ, Zhu C, Guo W, Devarkar SC, Wu S, Lin J, Zhang K, Liu J, Lomakin IB, Xiong Y (2025). Visualizing the translation landscape in human cells at high resolution. Nature Communications.

[bib125] Zivanov J, Nakane T, Forsberg BO, Kimanius D, Hagen WJ, Lindahl E, Scheres SH (2018). New tools for automated high-resolution cryo-EM structure determination in RELION-3. eLife.

[bib126] Zottig X, Huang CY, Seraj Z, Grigorieff N, Korostelev AA (2025). Structural Basis for Non-AUG Translation Regulation by 5MPs. bioRxiv.

