# Supplementary material for: In extracto cryo-EM reveals eEF2 as a major hibernation factor on 60S and 80S particles
Source: eLife. 2026 Sep 3;15:RP110114. doi: 10.7554/eLife.110114 (PMC13541302; doi:10.7554/eLife.110114)
Supplement: Supplementary file 2 [file elife-110114-supp1.docx]

**SUPPLEMENTARY INFORMATION FOR:**

***In extracto* cryo-EM reveals eEF2 as a major hibernation factor on 60S and 80S particles**

Zahra Seraj^1^, Ximena Zottig^1,2^, Chun-Ying Huang^1^, Anna B. Loveland^1^, Stephen Diggs^1^, Emily Sholi^1^, Nikolaus Grigorieff^1,2#^, Andrei A. Korostelev^1#^

^1^ RNA Therapeutics Institute, UMass Chan Medical School, 368 Plantation Street, Worcester, MA 01605, USA.

^2^ Howard Hughes Medical Institute, UMass Chan Medical School, 368 Plantation Street, Worcester, MA 01605, USA.

^#^ Co-corresponding authors: [niko@grigorieff.org](mailto:niko@grigorieff.org); [Andrei.Korostelev@umassmed.edu](mailto:Andrei.Korostelev@umassmed.edu)

**Contents:**

Supplementary Table 1-5

Supplementary Figures 1-9

Supplementary References

**Table A.** Particle picking strategies for the RRL+angiogenin dataset.

| **Strategy** | **Particles picked** | **Best resolution of classes** | **Classes observed** | |
| --- | --- | --- | --- | --- |
| *cis*TEM (Best 600 micrographs, CTF fit 2.5-3.2 Å) | ~8000 | >30 Å, would not align | none | |
| *cis*TEM (full stack)/ Frealign | 272,478 | >30 Å, would not align | none | |
| Relion v3 (full stack, picked with crYOLO trained on best 6216 particles from handpicking) | 72,431 or subsets | >30 Å, would not align | none | |
| Relion v3 (full stack, LoG picked and culled for bad ice and by lnCtfMaxRes ={1,4}) | 83,055 | 14 Å | 1) 80S+Ang+TC |  |
| Relion v3 (full stack, LoG picked and culled for bad ice and CTF, choosing high-resolution 2D classes) | 52,580 | < 6.6 Å  (binned) | 1) 80S+Ang+TC |  |
| Relion v3 (full stack, LoG picked and culled of bad ice, bad CTF, bad 2D classes, and selecting highest resolution 3D class for 2^nd^ round of subclassification) | 16,848 | 3.2 Å | 1) 80S+Ang+TC 2) 80S+eEF2 stalled  3) 60S | |
| Hand picking (***best*** 600 micrographs, refine picks, recenter...)/ Frealign v9 | 6216 | 3.5 Å | 1) 80S+Ang+TC 2) 80S+Ang+weakTC 3) 80S+eEF2 stalled  4) 60S | |
| 2DTM (~1600 micrographs)/ Frealign v9 | 37,859 | 3.1 Å | 1) 80S+Ang+TC 2) 80S+Ang 3) 80S+TC  4) 80S+2tRNAs+eEF2 5) 80S+eEF2 stalled  6) 60S | |
| 2DTM (full stack)/ Frealign v9 | 88,488 | 3.0 Å | 1) 80S+Ang+TC with substates 2) 80S+Ang 3) 80S+TC with substates 4) 80S+2tRNAs+eEF2 with substates 5) 80S+eEF2 stalled  6) 60S | |

**Table B.** Ribosome states observed in lysates from different cell types.

| **Ribosome status**  *Cell type* | **Elongating**  **(with mRNA and tRNAs and/or eEF1A/eEF2)** | **Hibernating** | **60S** | **Other classes** | **# particles**  **and Resolution (average initial reconstruction)** |
| --- | --- | --- | --- | --- | --- |
| *MCF-7 lysate* | 71.9% | 18% | 9.9% | - | 89,484-2.7 Å |
| *MCF-7 starved, lysate* | 57.6% | 27.3% | 14.9% | - | 88,560-2.6 Å |
| *BSC-1 lysate* | 58.3% | 32.6% | 6.5% | 2.2 %  (ABCE1/eRF1) | 525,827-2.2 Å |
| *RRL with mRNA* | 12.4% | 58.6% | 26% | 3% (initiation) | 209,874-2.2 Å |
| *HeLa cells (2DTM/cryo-EM; Zheng et al, 2024)* [1] | 70.1% | 29.9% | Unknown | - | 2.2 Å |
| *HEK293 cells, ER-bound 80S (cryo-ET, Gemmer et al,* 2023) [2] | 90% | 9.9% | Unknown | - | 4.4 Å |

**Table C.** Rotation of the 40S small ribosomal subunit head or body relative to nonrotated 80S ribosomes: with an accommodation-like eEF1A•aa-tRNA (PDB ID 5LZS [3]) or with tRNAs in three classical sites (this work). Negative numbers report on the “underrotated” subunits, i.e., rotated in the direction opposite to that measured from non-rotated (classical) to rotated (hybrid-state) pre-translocation ribosomes.

|  | Relative to 80S with eEF1A•aa-tRNA and P-tRNA  (PDB 5LZS) | | Relative to 80S with 3 tRNAs:  A, P, E  (this work) | |
| --- | --- | --- | --- | --- |
|  | **Head** | **Body** | **Head** | **Body** |
| **eEF2+eIF5A+SERBP1** | 16.35° | 5.28° | 16.38° | 3.44° |
| **eEF2+CCDC124+LARP1** | 18.50° | -0.62° | 18.61° | -4.60° |
| **eEF2+IFRD2+LARP1** | 18.07° | -1.05° | 18.60° | -4.61° |
| **IFRD2+LARP1** | 18.07° | -1.05° | 18.25° | -5.01° |

**Table D.** Cryo-EM data collection and model refinement statistics for 80S and 60S complexes from RRL.

|  | **80S•eEF2**  **•eIF5A•SERBP1** | **80S•eEF2**  **•IFRD2•LARP1** | **80S•IFRD2**  **•LARP1** | **80S•eEF2**  **•CCDC124•LARP1** | **60S•eEF2 (open)** | **60S•eEF2 (closed)** |  |
| --- | --- | --- | --- | --- | --- | --- | --- |
| **RCSB PDB ID**  **EMDB Accession ID** | 11IQ  EMD-75724 | 11JJ  EMD-75740 | 11KH  EMD-75768 | 11HG  EMD-75689 | 11HV  EMD-75704 | 11HE  EMD-75687 |  |
| **Data collection and processing** | | | | | | | |
| Nominal magnification | 60,241x | 60,241x | 60,241x | 60,241x | 60,241x | 60,241x |  |
| Voltage (kV) | 300 | 300 | 300 | 300 | 300 | 300 |  |
| Electron exposure (e^−^/Å^2^) | 30 | 39 | 39 | 30 | 39 | 39 |  |
| Defocus range (μm) | 0.7-2 | 0.7-2 | 0.7-2 | 0.7-2 | 0.7-2 | 0.7-2 |  |
| Pixel size (Å) | 0.83 | 0.83 | 0.83 | 0.83 | 0.83 | 0.83 |  |
| Symmetry imposed | C1 | C1 | C1 | C1 | C1 | C1 |  |
| Initial particle images (no.) | 512,697 | 1,079,101 | 1,079,101 | 512,697 | 1,079,101 | 1,079,101 |  |
| Final particle images (no.) | 198,920 | 16,727 | 10,932 | 8,501 | 15,960 | 22,323 |  |
| Map resolution (Å)[^**^](https://pmc.ncbi.nlm.nih.gov/articles/PMC11912008/#TFN2) | 2.4 | 3.0 | 3.2 | 2.7 | 2.9 | 2.9 |  |
| FSC threshold | 0.143 | 0.143 | 0.143 | 0.143 | 0.143 | 0.143 |  |
| **Refinement** | | | | | | | |
| Initial model used (PDB code) | 7TOR | 7TOR | 7TOR | 7TOR | 7TOR | 7TOR |  |
| **Model resolution (Å)**[**^*^**](https://pmc.ncbi.nlm.nih.gov/articles/PMC11912008/#TFN1) | | | | | | | |
| FSC threshold | 0.143 | 0.143 | 0.143 | 0.143 | 0.143 | 0.143 |  |
| Correlation Coefficient (cc_mask)[^*^](https://pmc.ncbi.nlm.nih.gov/articles/PMC11912008/#TFN1) | 0.84 | 0.80 | 0.79 | 0.79 | 0.76 | 0.75 |  |
| **Model composition**[**^*^**](https://pmc.ncbi.nlm.nih.gov/articles/PMC11912008/#TFN1) | | | | | | | |
| Non-hydrogen atoms | 224497 | 224042 | 218411 | 221974 | 147591 | 147624 |  |
| Protein residues | 13401 | 13271 | 12433 | 12998 | 8155 | 8159 |  |
| RNA residues | 5467 | 5497 | 5539 | 5497 | 3825 | 3825 |  |
| **R.m.s. deviations from ideal**[**^*^**](https://pmc.ncbi.nlm.nih.gov/articles/PMC11912008/#TFN1)**^,^**[**^§^**](https://pmc.ncbi.nlm.nih.gov/articles/PMC11912008/#TFN4) | | | | | | | |
| Bond lengths (Å) | 0.002 | 0.003 | 0.003 | 0.002 | 0.009 | 0.004 |  |
| Bond angles (°) | 0.62 | 0.61 | 0.76 | 0.60 | 0.88 | 0.64 |  |
| **Validation**[**^*^**](https://pmc.ncbi.nlm.nih.gov/articles/PMC11912008/#TFN1) | | | | | | | |
| MolProbity score | 1.87 | 1.92 | 1.96 | 1.95 | 2.45 | 1.98 |  |
| Clashscore | 6.12 | 7.02 | 7.30 | 7.70 | 8.77 | 6.60 |  |
| **Ramachandran plot**[**^*^**](https://pmc.ncbi.nlm.nih.gov/articles/PMC11912008/#TFN1) | | | | | | | |
| Favored (%) | 97.68 | 97.71 | 97.36 | 97.74 | 95.94 | 96.88 |  |
| **Validation (RNA)**[**^*^**](https://pmc.ncbi.nlm.nih.gov/articles/PMC11912008/#TFN1) | | | | | | | |
| Good sugar pucker (%) | 98.82 | 98.83 | 98.91 | 98.83 | 98.69 | 98.77 |  |
| Good backbone (%)[^#^](https://pmc.ncbi.nlm.nih.gov/articles/PMC11912008/#TFN5) | 99.24 | 99.23 | 99.01 | 99.23 | 99.08 | 99.32 |  |

^*^Statistics calculated in Phenix v.2.0^[4].^

^**^FSC_part from Frealign v. 9.11^[5].^

^#^RNA backbone suites that fall into recognized rotamer conformations defined by MolProbity^[6].^

**Table E.** Percentages of particles corresponding to 80S or 60S classes in RRL with NLuc mRNA and in RRL without NLuc mRNA.

| Ribosome state | Factors^#^ | RRL+mRNA | RRL-mRNA |
| --- | --- | --- | --- |
| Initiation | eIF5B | 2.4% | 5.0% |
| Elongation | eEF1A+3 tRNA | 7.7% | 4.3% |
|  | eEF2+2 tRNA | 1.0% | X^*^ |
|  | A/P and P/E tRNAs | X | 1.2% |
|  | DRG1/2 | 2.0% | 0.7% |
| Hibernation | eEF2+eIF5A+SERBP1 | 51% | 46.5% |
|  | eEF2+E-tRNA+SERBP1 | 5.7% | 2.0% |
|  | eEF2+CCDC124+LARP1 | 1.1% | 5.4% |
|  | eEF2+IFRD2+LARP1 | 1.0% | 3.1% |
|  | IFRD2+LARP1 | X | 1.7% |
| 60S | None | 19.2% | 26.4% |
|  | eEF2 | 8.8% | 3.5% |

^#^ EBP1 was identified in all the classes and is not specified in the table

^*^X denotes the states that were absent after 3D classifications

**Supplementary references**

1.	Zheng, W., et al., *Visualizing the translation landscape in human cells at high resolution.* Nature Communications, 2025. **16**(1): p. 10757.

2.	Gemmer, M., et al., *Visualization of translation and protein biogenesis at the ER membrane.* Nature, 2023. **614**(7946): p. 160-167.

3.	Shao, S., et al., *Decoding mammalian ribosome-mRNA states by translational GTPase complexes.* Cell, 2016. **167**(5): p. 1229-1240. e15.

4.	Liebschner, D., et al., *Macromolecular structure determination using X-rays, neutrons and electrons: recent developments in Phenix.* Biological Crystallography, 2019. **75**(10): p. 861-877.

5.	Lyumkis, D., et al., *Likelihood-based classification of cryo-EM images using FREALIGN.* Journal of structural biology, 2013. **183**(3): p. 377-388.

6.	Williams, C.J., et al., *MolProbity: more and better reference data for improved all‐atom structure validation.* Protein Science, 2018. **27**(1): p. 293-315.

7.	Jaskolowski, M., et al., *Molecular basis of the TRAP complex function in ER protein biogenesis.* Nature Structural & Molecular Biology, 2023. **30**(6): p. 770-777.

8.	Greber, B.J., et al., *Insertion of the biogenesis factor Rei1 probes the ribosomal tunnel during 60S maturation.* Cell, 2016. **164**(1): p. 91-102.

9.	Akers, J.F., et al., *ZNF574 is a quality control factor for defective ribosome biogenesis intermediates.* Molecular Cell, 2025. **85**(10): p. 2048-2060. e9.

10.	Nurullina, L., et al., *Cryo‐EM structure of the inactive ribosome complex accumulated in chick embryo cells in cold‐stress conditions.* FEBS letters, 2024. **598**(5): p. 537-547.
