# Supplementary material for: In extracto cryo-EM reveals eEF2 as a major hibernation factor on 60S and 80S particles
Source: eLife. 2026 Sep 3;15:RP110114. doi: 10.7554/eLife.110114 (PMC13541302; doi:10.7554/eLife.110114)
Supplement: Peer review history

# Editor report: eLife Assessment

**DOI:** 10.7554/eLife.110114.3.sa0

**Authors:** Axel T Brunger

**Affiliations:** Stanford University School of Medicine, Howard Hughes Medical Institute United States

**Keywords:** Compelling, Important

In this **important** work, it is demonstrated that certain high-resolution cryo-EM structures can be obtained by using concentrated cell extracts without purification. The **compelling** results with the mammalian ribosomes demonstrate the utility of this approach for this molecule and complexes with elongation factor 2. Moreover, this work also demonstrates the utility of 2D template matching for particle picking for structure determination by single-particle averaging pipelines.

# Referee report: Reviewer #1 (Public review)

**DOI:** 10.7554/eLife.110114.3.sa1

Summary:

The manuscript by Seraj et al. introduces a transformative structural biology methodology termed "*in extracto* cryo-EM." This approach circumvents the traditional, often destructive, purification processes by performing single-particle cryo-EM directly on crude cellular lysates. By utilizing high-resolution 2D template matching (2DTM), the authors localize ribosomal particles within a complex molecular "crowd," achieving near-atomic resolution (~2.2 Å). The biological centerpiece of the study is the characterization of the mammalian translational apparatus under varying physiological states. The authors identify elongation factor 2 (eEF2) as a nearly universal hibernation factor, remarkably present not only on non-translating 80S ribosomes but also on 60S subunits. The study provides a detailed structural atlas of how eEF2, alongside factors like SERBP1, LARP1, and IFRD2, protects the ribosome's most sensitive functional centers (the PTC, DC, and SRL) during cellular stress.

Strengths:

The "*in extracto*" approach is a significant leap forward. It offers the high resolution typically reserved for purified samples while maintaining the "molecular context" found in *in situ* studies. This addresses a major bottleneck in structural biology: the loss of transiently bound or labile factors during biochemical purification.

The finding that eEF2 binds and sequesters 60S subunits is a major biological insight. This suggests a "pre-assembly" hibernation state that allows for rapid mobilization of the translation machinery once stress is relieved, which was previously uncharacterized in mammalian cells.

The authors successfully captured eIF5A and various hibernation factors in states that are traditionally disrupted. The identification of eIF5A across nearly all translating and non-translating states highlights the power of this method to detect ubiquitous but weakly bound regulators.

The manuscript beautifully illustrates the "shielding" mechanism of the ribosome. By mapping the binding sites of eEF2 and its co-factors, the authors provide a clear chemical basis for how the cell prevents nucleolytic cleavage of ribosomal RNA during nutrient deprivation.

Weaknesses:

While 2DTM is a powerful search tool, it inherently relies on a known structural "template." There is a risk that this methodology may be "blind" to highly divergent or novel macromolecular complexes that do not share sufficient structural similarity with the search model. The authors should discuss the limitations of using a vacant 60S/80S template in identifying highly remodeled stress-induced complexes. For instance, what happens if an empty 40S subunit is used as template? In the current work, while 60S and 80S particles are picked, none are 40S. The authors should comment on this.

In the GTPase center, the authors identify density for "DRG-like" proteins. However, due to limited local resolution in that specific region, they are unable to definitively distinguish between DRG1 and DRG2. While the structural similarity is high, the functional implications differ, and the identification remains somewhat speculative. The authors should acknowledge this in the text.

While "*in extracto*" is superior to purified SPA, the act of cell lysis (even rapid permeabilization) still involves a change in the chemical environment (pH, ion concentration, and dilution of metabolites). The authors could strengthen the manuscript by discussing how post-lysis changes might affect the occupancy of factors like GTP vs. GDP states.

The study provides excellent snapshots of stationary states (translating vs. hibernating), but the kinetic transition-specifically how the 60S-eEF2 complex is recruited back into active translation-is not well discussed. On page 13, the authors present eEF2 bound to 60S but do not mention anything regarding which nucleotide is bound to the factor. It only becomes clear that it is GDP after looking at Figure S9. This should be clarified in the text. Similarly, the observations that eEF2 is bound to GDP in the 60S and 80S raises the questions as to how the factor dissociates from the ribosome. This could also be discussed.

Overall Assessment:

This work reported in this manuscript likely represents the future of structural proteomics. The combination of high-resolution structural biology with minimal sample perturbation provides a new standard for investigating the cellular machines that govern life. After addressing minor points regarding template bias, protein identification, and transition dynamics, this work may become a landmark in the field of translation.

Comments on revisions:

In the revised version of the manuscript, the authors have addressed my prior concerns.

# Referee report: Reviewer #2 (Public review)

**DOI:** 10.7554/eLife.110114.3.sa2

In this manuscript, the authors describe using "*in extracto*" cryo-EM to obtain high-resolution structures of mammalian ribosomes from concentrated cell extracts without further purification or reconstitution. This approach aims to solve two related problems. The first is that purified ribosomes often lose cellular cofactors, which are often reconstituted *in vitro*; this precludes the ability to find novel interactions. The second is that while it is possible to perform cryo-EM on cellular lamella, FIB milling is a slow and laborious process, making it unfeasible to collect datasets sufficiently large to allow for high-resolution structure determination. Extracts should contain all cellular cofactors and allow for grid preparation similar to standard single-particle analysis (SPA) approaches. While cryo-EM of cell extracts is not in itself novel, this manuscript uses 2D template matching (2DTM) for particle picking prior to structure determination using more standard SPA pipelines. This should allow for improved picking over other approaches, in order to obtain in large datasets for high-resolution SPA.

This manuscript has two main results: novel structures of ribosomes in hibernating states; and a proof-of-principle for *in extracto* cryo-EM using 2DTM. Overall, I think the results presented here are strong and serve as a proof-of-principle for an approach that may be useful to many others.

Comments on revisions:

This current draft addresses my prior comments regarding usability for readers through the addition of text describing how parameters were optimized as well as an additional supplementary figure outlining the processing workflow. With these additions, I have no further comments.

# Referee report: Reviewer #3 (Public review)

**DOI:** 10.7554/eLife.110114.3.sa3

Summary:

The authors describe a new structural biology framework termed "*in extracto* cryo-EM," which aims to bridge the gap between single-particle cryo-EM of purified complexes and *in situ* cryo-electron tomography (cryo-ET). By utilizing high-resolution 2D template matching (2DTM) on mammalian cell lysates, the authors sought to visualize the translational apparatus in a near-native environment while maintaining near-atomic resolution. The study identifies elongation factor 2 (eEF2) as a major hibernation factor bound to both 60S and 80S particles and describes a variety of hibernation scenarios involving factors such as SERBP1, LARP1, and CCDC124.

Strengths:

(1) The use of 2DTM effectively overcomes the signal-to-noise challenges posed by the dense and viscous nature of cellular extracts, yielding maps as high as 2.2 Å.

(2) The discovery of eEF2-GDP as a ubiquitous shield for ribosomal functional centers, particularly its unexpected stabilization on the 60S subunit, provides a compelling model for ribosome preservation during stress.

Weaknesses:

(1) Representative nature of cell samples and lower detection limit

The cells used in this study (MCF-7, BSC-1, and RRL) are either fast-growing cancer cell lines or specialized protein-synthetic systems. For cells with naturally low ribosomal abundance (such as quiescent primary cells), achieving the target concentration (e.g., A260 > 1000 ng/uL) would require an exponentially larger starting cell population.

Is there a defined lower limit of ribosomal concentration in the raw lysate below which the 2DTM algorithm fails to yield high-resolution classes? In ribosome-sparse lysates, A260 becomes an unreliable proxy for ribosome density due to the high background of other RNA species and proteins. How do the authors estimate specific ribosome abundance in such heterogeneous fields?

(2) Quantitation in heterogeneous lysates and crowding effects

The authors utilize A260 as a key quality control measure before grid preparation. However, if extreme physical concentration is required to see enough particles, the background concentration of other cytoplasmic components also increases. This may lead to molecular crowding or sample viscosity that interferes with the formation of optimal thin ice. How do the authors calculate or estimate the specific abundance of ribosomes in the cryo-EM field of view when they represent a much smaller percentage of the total cellular content?

(3) Optimization of sample preparation

The authors describe lysates as dense and viscous, requiring multiple blotting steps (2-3 times) for 3-8 seconds. Have the authors tested whether a larger molecular weight cutoff (e.g., 100 kDa) during concentration could improve the ribosome-to-background ratio without losing small factors like eIF5A (approx. 17 kDa)? Could repeated blotting of a concentrated, viscous lysate introduce shearing forces or increased exposure to the air-water interface that perturbs the native conformation of the complexes?

(4) The regulatory switch and mechanism of eEF2

The finding that eEF2-GDP occupies dormant ribosomes is striking. What drives eEF2 from its canonical role in translocation to this hibernation state? Is this transition purely driven by stoichiometry (lack of mRNA/tRNA) and the GDP/GTP ratio, or is there a role for post-translational modifications? How do these eEF2-bound dormant ribosomes rapidly re-enter the translation pool upon stress relief?

(5) Hibernation diversity and LARP1 contextualization

The study reveals that hibernation strategies vary across cell types. Does the high hibernation rate in RRL reflect a physiological state, or does it hint at "preparation-induced stress" due to resource exhaustion or mRNA degradation in the cell-free system? How do the authors reconcile their discovery of LARP1 on 80S particles with recent 2024 reports that primarily describe LARP1 as an SSU-bound repressor?

Comments on revisions:

The authors have addressed the issues I had raised in my initial review. The additional data and clarifications provided in the revision are satisfactory. I have no further recommendations.

Thanks to the authors for their efforts.

# Author comment: Author response

**DOI:** 10.7554/eLife.110114.3.sa4

**Authors:** Zahra Seraj, Ximena Zottig, ChunYing Huang, Anna B Loveland, Stephen Diggs, Emily Sholi, Nikolaus Grigorieff, Andrei A Korostelev

**Affiliations:** University of Massachusetts Chan Medical School Worcester United States; University of Massachusetts Chan Medical School Worcester United States; University of Massachusetts Chan Medical School Worcester United States; University of Massachusetts Chan Medical School Worcester United States; University of Massachusetts Chan Medical School Worcester United States; University of Massachusetts Chan Medical School Worcester United States; Howard Hughes Medical Institute Worcester, MA United States; University of Massachusetts Chan Medical School Worcester United States

The following is the authors’ response to the original reviews.

**eLife Assessment**

In this important work, it is demonstrated that certain high-resolution cryo-EM structures can be obtained by using concentrated cell extracts without purification. The compelling results with the mammalian ribosomes demonstrate the utility of this approach for this molecule and complexes with elongation factor 2. Moreover, this work also demonstrates the utility of 2D template matching for particle picking for structure determination by single-particle averaging pipelines.

We thank the reviewers for their valuable comments and suggestions, which have helped us to improve the manuscript. We provide a response to the referees’ comments below.

**Public Reviews:**

**Reviewer #1 (Public review):**

Summary:

The manuscript by Seraj et al. introduces a transformative structural biology methodology termed "*in extracto* cryo-EM." This approach circumvents the traditional, often destructive, purification processes by performing single-particle cryo-EM directly on crude cellular lysates. By utilizing high-resolution 2D template matching (2DTM), the authors localize ribosomal particles within a complex molecular "crowd," achieving near-atomic resolution (~2.2 Å). The biological centerpiece of the study is the characterization of the mammalian translational apparatus under varying physiological states. The authors identify elongation factor 2 (eEF2) as a nearly universal hibernation factor, remarkably present not only on non-translating 80S ribosomes but also on 60S subunits. The study provides a detailed structural atlas of how eEF2, alongside factors like SERBP1, LARP1, and IFRD2, protects the ribosome's most sensitive functional centers (the PTC, DC, and SRL) during cellular stress.

Strengths:

The "*in extracto*" approach is a significant leap forward. It offers the high resolution typically reserved for purified samples while maintaining the "molecular context" found in *in situ* studies. This addresses a major bottleneck in structural biology: the loss of transiently bound or labile factors during biochemical purification.

The finding that eEF2 binds and sequesters 60S subunits is a major biological insight. This suggests a "pre-assembly" hibernation state that allows for rapid mobilization of the translation machinery once stress is relieved, which was previously uncharacterized in mammalian cells.

The authors successfully captured eIF5A and various hibernation factors in states that are traditionally disrupted. The identification of eIF5A across nearly all translating and non-translating states highlights the power of this method to detect ubiquitous but weakly bound regulators.

The manuscript beautifully illustrates the "shielding" mechanism of the ribosome. By mapping the binding sites of eEF2 and its co-factors, the authors provide a clear chemical basis for how the cell prevents nucleolytic cleavage of ribosomal RNA during nutrient deprivation.

Weaknesses:

(1) While 2DTM is a powerful search tool, it inherently relies on a known structural "template." There is a risk that this methodology may be "blind" to highly divergent or novel macromolecular complexes that do not share sufficient structural similarity with the search model. The authors should discuss the limitations of using a vacant 60S/80S template in identifying highly remodeled stress-induced complexes. For instance, what happens if an empty 40S subunit is used as a template? In the current work, while 60S and 80S particles are picked, none are 40S. The authors should comment on this.

Thank you for your comment. As noted by the reviewer, 2DTM inherently favors particles that share sufficient similarity with the search template and may underrepresent highly remodeled or structurally divergent complexes. Importantly, once particles are identified, subsequent 2D/3D classification and refinement are not constrained by the template used for particle picking. Consistent with this, we observe classes displaying additional or altered densities absent in the original template, indicating that template matching does not preclude the detection of remodeled ribosomal states, although highly divergent species may still escape detection.

Regarding the use of a 40S subunit as a template for 2DTM, we tested two templates: a complete 40S subunit and the 40S body alone. Using these 40S templates, we captured several 40S-, 43S-, and 48S-containing complexes, as well as 80S particles. As expected, no individual 60S classes emerge with 40S-TM. 40S-TM yielded 80S classes similar to those with 60-TM, although the number of particles was lower than that in 60S template matching, resulting in lower resolution of these classes. Since this study focuses on ribosome hibernation, we chose to proceed with the 60S-TM results and do not report results using 40S-TM. We reported 40S-TM results in another study from our groups (Zottig et al., bioRxiv, 2025), which focuses on translation initiation on 40S subunits and was deposited as preprint after this submission.

We have added a comment and reference describing the use of the 40S template in the initial section of Results and Discussion: “This result echoes our concurrent finding that using 40S or partial 40S templates yields a variety of initiation complexes and 80S classes, revealing densities beyond those in the template [44].”

(2) In the GTPase center, the authors identify density for "DRG-like" proteins. However, due to limited local resolution in that specific region, they are unable to definitively distinguish between DRG1 and DRG2. While the structural similarity is high, the functional implications differ, and the identification remains somewhat speculative. The authors should acknowledge this in the text.

We agree with this comment and address it in the main text:

“Whereas the overall shape and secondary structure resemble DRG1 or DRG2, the local resolution is insufficient to distinguish between these or other similarly structured proteins. Both yeast and mammalian counterparts are reported to function with a companion factor (Tma146p or Gir2 in yeast; or DFRP1 and DFRP2 in mammals), but our maps do not contain density that could correspond to DFRP1/2 near the putative DRG1/2 density. Future work will elucidate the function of these or other DRG-like GTPases in the context of an elongation complex.”

(3) While "*in extracto*" is superior to purified SPA, the act of cell lysis (even rapid permeabilization) still involves a change in the chemical environment (pH, ion concentration, and dilution of metabolites). The authors could strengthen the manuscript by discussing how post-lysis changes might affect the occupancy of factors like GTP vs. GDP states.

Thank you for pointing this out. Cell lysis can indeed lead to a change in the chemical environment, although we do not know how post-lysis changes may specifically affect the occupancy of factors, such as GTP- vs. GDP-bound states. We tried to minimize this effect by performing a rapid permeabilization. Our efforts to optimize our protocols are ongoing, and we expect to have a better answer to this question in the future.

Nevertheless, to address this reviewer’s concern, our discussion states: “Additional optimization of buffer conditions may be required to more accurately represent the translation states observed in cells, as ionic conditions are known to affect the conformation of the ribosomes (e.g. rotated/non-rotated) and binding of protein factors”.

(4) The study provides excellent snapshots of stationary states (translating vs. hibernating), but the kinetic transition, specifically how the 60S-eEF2 complex is recruited back into active translation, is not well discussed. On page 13, the authors present eEF2 bound to 60S but do not mention anything regarding which nucleotide is bound to the factor. It only becomes clear that it is GDP after looking at Figure S9. This should be clarified in the text. Similarly, the observations that eEF2 is bound to GDP in the 60S and 80S raise questions as to how the factor dissociates from the ribosome. This could also be discussed.

Thank you for bringing this to our attention. We now state in the main text that eEF2 is bound with GDP on the 60S subunit.

As for the kinetic transitions of 60S-eEF2 complexes, like this reviewer, we are fascinated by the possible roles and mechanisms of the 60S-eEF2 complex. The averaged particle ensembles derived from cryo-EM data do not report on the kinetics or transition pathways directly. We acknowledge in the main text that “Future studies will bring insights into the roles of the protein(s) and into the functions and transitions of 60S•eEF2 complexes to the pool of translating ribosomes”.

Overall Assessment:

The work reported in this manuscript likely represents the future of structural proteomics. The combination of high-resolution structural biology with minimal sample perturbation provides a new standard for investigating the cellular machines that govern life. After addressing minor points regarding template bias, protein identification, and transition dynamics, this work may become a landmark in the field of translation.

**Reviewer #2 (Public review):**

In this manuscript, the authors describe using "*in extracto*" cryo-EM to obtain high-resolution structures of mammalian ribosomes from concentrated cell extracts without further purification or reconstitution. This approach aims to solve two related problems. The first is that purified ribosomes often lose cellular cofactors, which are often reconstituted *in vitro*; this precludes the ability to find novel interactions. The second is that while it is possible to perform cryo-EM on cellular lamella, FIB milling is a slow and laborious process, making it unfeasible to collect datasets sufficiently large to allow for high-resolution structure determination. Extracts should contain all cellular cofactors and allow for grid preparation similar to standard single-particle analysis (SPA) approaches. While cryo-EM of cell extracts is not in itself novel, this manuscript uses 2D template matching (2DTM) for particle picking prior to structure determination using more standard SPA pipelines. This should allow for improved picking over other approaches in order to obtain large datasets for high-resolution SPA.

This manuscript has two main results: novel structures of ribosomes in hibernating states; and a proof-of-principle for *in extracto* cryo-EM using 2DTM. Overall, I think the results presented here are strong and serve as a proof-of-principle for an approach that may be useful to many others. However, without presenting the logic of how parameters were optimized, this manuscript is limited in its direct utility to readers.

Thank you for this valuable comment. We have expanded our Methods section “Optimization of 2DTM in RRL data “to present the logic behind parameter optimization, with the paragraph beginning with “We optimized high-resolution template matching procedures…”

**Reviewer #3 (Public review):**

Summary:

The authors describe a new structural biology framework termed "*in extracto* cryo-EM," which aims to bridge the gap between single-particle cryo-EM of purified complexes and *in situ* cryo-electron tomography (cryo-ET). By utilizing high-resolution 2D template matching (2DTM) on mammalian cell lysates, the authors sought to visualize the translational apparatus in a near-native environment while maintaining near-atomic resolution. The study identifies elongation factor 2 (eEF2) as a major hibernation factor bound to both 60S and 80S particles and describes a variety of hibernation scenarios involving factors such as SERBP1, LARP1, and CCDC124.

Strengths:

(1) The use of 2DTM effectively overcomes the signal-to-noise challenges posed by the dense and viscous nature of cellular extracts, yielding maps as high as 2.2 Å.

(2) The discovery of eEF2-GDP as a ubiquitous shield for ribosomal functional centers, particularly its unexpected stabilization on the 60S subunit, provides a compelling model for ribosome preservation during stress.

Weaknesses:

(1) Representative nature of cell samples and lower detection limit

The cells used in this study (MCF-7, BSC-1, and RRL) are either fast-growing cancer cell lines or specialized protein-synthetic systems. For cells with naturally low ribosomal abundance (such as quiescent primary cells), achieving the target concentration (e.g., A260 > 1000 ng/uL) would require an exponentially larger starting cell population.

Is there a defined lower limit of ribosomal concentration in the raw lysate below which the 2DTM algorithm fails to yield high-resolution classes? In ribosome-sparse lysates, A260 becomes an unreliable proxy for ribosome density due to the high background of other RNA species and proteins. How do the authors estimate specific ribosome abundance in such heterogeneous fields?

We have not tested these specific points, but we found that 2DTM can successfully result in high-resolution reconstructions even with 1-2 particles per micrograph. This would require a substantially larger dataset than in this work yet could provide a viable strategy for diluted or low-abundance samples. Other optimizations, including lysate concentration, may help as well. We have the following text to reflect these points:

“Additional optimization of buffer conditions may be required to more accurately represent the translation states observed in cells, as ionic conditions are known to affect the conformation of the ribosomes (e.g. rotated/non-rotated) and binding of protein factors [91-94]. For cells or samples with lower abundance of ribosomes or other macromolecules/complexes of interest, a lysate concentration step or collection of a larger dataset may be considered.”

(2) Quantitation in heterogeneous lysates and crowding effects

The authors utilize A260 as a key quality control measure before grid preparation. However, if extreme physical concentration is required to see enough particles, the background concentration of other cytoplasmic components also increases. This may lead to molecular crowding or sample viscosity that interferes with the formation of optimal thin ice. How do the authors calculate or estimate the specific abundance of ribosomes in the cryo-EM field of view when they represent a much smaller percentage of the total cellular content?

We reported A260 as a reference that may be useful to achieve particle distributions resembling those in our work, rather than as a key quality control measure. Accordingly, we do not use it to estimate ribosome concentration or the specific abundance of ribosomes; instead, we’d recommend adjusting the sample concentration/dilution by grid screening.

This reviewer mentions the important aspect of ice thickness. We found that the highest population of ribosome particles is found in thicker ice regions, and these particles have been used to make up the majority of our datasets leading to high-resolution reconstructions. We have added this observation to “Optimization of 2DTM in RRL data”.

(3) Optimization of sample preparation

The authors describe lysates as dense and viscous, requiring multiple blotting steps (2-3 times) for 3-8 seconds. Have the authors tested whether a larger molecular weight cutoff (e.g., 100 kDa) during concentration could improve the ribosome-to-background ratio without losing small factors like eIF5A (approx. 17 kDa)? Could repeated blotting of a concentrated, viscous lysate introduce shearing forces or increased exposure to the air-water interface that perturbs the native conformation of the complexes?

We strived to minimize the number of steps in sample preparation, so we did not extensively test concentration steps. We also found that a concentration step can be omitted; the eIF5A-containing structure from the RRL dataset was determined without this step. We agree with the reviewer that repeated blotting may change ribosome complex equilibrium and result in a different distribution of functional states than in cells. However, we did not find evidence of perturbation of the native conformations of complexes, as the positions of ribosomes and factors are nearly identical to those observed in previous studies, including the recent high-resolution structures from cells that we cite.

(4) The regulatory switch and mechanism of eEF2

The finding that eEF2-GDP occupies dormant ribosomes is striking. What drives eEF2 from its canonical role in translocation to this hibernation state? Is this transition purely driven by stoichiometry (lack of mRNA/tRNA) and the GDP/GTP ratio, or is there a role for post-translational modifications? How do these eEF2-bound dormant ribosomes rapidly re-enter the translation pool upon stress relief?

We are glad that this reviewer is fascinated by the eEF2-GDP occupancy on dormant ribosome (just like we are)! These are important open questions that require further research, as our cryo-EM analyses cannot directly address the kinetic or mechanistic aspects of the mentioned processes. We did explore the known modification/phosphorylation sites in eEF2 densities but did not find evidence for such modifications, which does not rule out the possibility of transient or new modifications.

(5) Hibernation diversity and LARP1 contextualization

The study reveals that hibernation strategies vary across cell types. Does the high hibernation rate in RRL reflect a physiological state, or does it hint at “preparation-induced stress” due to resource exhaustion or mRNA degradation in the cell-free system? How do the authors reconcile their discovery of LARP1 on 80S particles with recent 2024 reports that primarily describe LARP1 as an SSU-bound repressor?

Based on the high abundance of hibernating ribosomes in RRL (relative to many other samples we have tested so far), we speculate that this scenario may result from the stresses induced during lysate preparation: first, the rabbits are treated with phenylhydrazine inducing cell stress, then lysates are treated with micrococcal nuclease to degrade endogenous mRNAs. In addition, the specialization of reticulocytes may contribute to the distinct expression of stress/hibernation factors.

As for LARP1, our finding is consistent with the 2024 work by Saba et al, who reported LARP1 binding to both 40S subunits and 80S ribosomes. They also noted that LARP1-bound ribosomes are “non-translating”, consistent with our structures.

**Recommendations for the authors:**

**Reviewer #1 (Recommendations for the authors):**

(1) In Figure 3, it would be easier for the reader if the authors would report the % of particles in each class. Also, indicating body rotation and head swiveling values would help.

Because our high-resolution maps result from a combination of data sets (e.g., RRL with an mRNA and RRL without an mRNA), we specify the particle percentages in the corresponding classification schemes in supplemental figures. To avoid excessive labeling in this figures, body rotation and head swiveling values for the new classes are shown in Figure 4.

(2) Page 16, what is 'elongation factor 1'? It doesn't seem the authors refer to eEF1A?

Thank you for pointing out this inconsistency, this is indeed eEF1A. We have corrected the text.

(3) Page 16, after 'individual 60S subunits', there is a missing full stop.

Thanks. Corrected.

**Reviewer #2 (Recommendations for the authors):**

I am not an expert in ribosome biology and do not have any specific comments on the various states presented here. Instead, I will mainly focus on the image processing aspects of this manuscript.

Major points:

(1) Were any AI-based particle pickers, such as crYOLO, topaz, or warp tested? While more traditional template-based or LoG pickers were shown to be inferior to 2DTM, it is unclear if AI methods would perform just as well. Given that a major point of this manuscript is the image processing pipeline, and that these AI tools have been widely adopted in the field, I think this is an important consideration.

We used other particle pickers before using 2DTM and have listed them in the Supplementary Information: please see Table S1 for a complete list of particle pickers evaluated in this study. Since our present work focuses on a sample preparation method, a more extensive evaluation of particle picking methods is beyond the scope of this study.

(2) While the methods used to obtain the structures presented are detailed, I think it would also be useful to provide some logic for how parameters were determined or optimized. This would serve as a useful foundation for readers who wish to try out this in an extracto approach on their own specimens. Some of these optimizations seem quite specific, such as optimization of angular search parameters, but with no clear logic: e.g., why is the out-plane search coarser than the in-plane search; what is the effect of increasing the angular step sizes? Some seem inconsistent, e.g., why is e2pdb2mrc.py sometimes used and the cisTEM simulate used other times? Some are poorly described, such as "the defocus search turned on for micrographs with thicker ice" where there is no mention of how ice thickness is assessed and how thick is too thick. I think a workflow figure with accompanying text would help the reader understand the logic used in this work and how to apply that logic to their own projects.

To address the comments in (2), we provide separate responses addressing each comment:

(1) Provide some logic for how parameters were determined or optimized:

The logic behind determining and optimizing search parameters is a balance between search precision and computational cost. In practice, users must weigh the benefit of finer sampling against the substantial increase in runtime, particularly for large datasets. For example, enabling defocus searching with a 200 Å step size and a 1000 Å range increases the computational time by approximately 11-fold compared to running the same search with defocus disabled (since each defocus plane in the positive and negative direction are searched), making such increases prohibitive, when GPU resources are limited. In such cases, reducing the defocus search to a 250 Å step size and a 500 Å range can dramatically shorten runtime while preserving nearly the same number of reliable matches. In summary, we found that optimizing the defocus search, in-plane, out-plane angles, and the image/micrograph pixel size can substantially reduce the processing speed while sacrificing only a small percentage of particles.

We have expanded our parameter optimization paragraph in “Optimization of 2DTM in RRL data”, as mentioned in a previous response.

(2) Some seem inconsistent, e.g., why is e2pdb2mrc.py sometimes used and the cisTEM simulate used other times?

e2pdb2mrc.py is simpler to use and was used in the beginning of the project. Later, we switched to using the simulate program since it preformed slightly better. Either software is suitable to generate templates for 2DTM.

(3) Some are poorly described, such as "the defocus search turned on for micrographs with thicker ice" where there is no mention of how ice thickness is assessed and how thick is too thick.

We did not quantitatively assess ice thickness; instead, we tested whether it is advantageous to include the defocus search. To this end, we first performed CTF estimation and grouped micrographs based on their fit resolution. From each group, we selected ten micrographs representing the highest and lowest fit resolutions. Template matching was then performed using identical parameters, once with defocus search enabled and once with it disabled. The number of picked particles for each micrograph under both conditions was compared. When a significant difference was observed most commonly for icy micrographs with low fit resolution we enabled defocus search for that group of images. The difference between having the defocus search on vs off sometimes resulted in having 2x more matches. We found these images/datasets appeared to have a higher background compared to in-vitro reconstituted samples. The template-matching results from these micrographs were subsequently combined with results from groups processed with defocus search disabled.

To address this point, we have included this description in “Optimization of 2DTM in RRL data”.

(4) I think a workflow figure with accompanying text would help the reader understand the logic used in this work and how to apply that logic to their own projects.

Thanks for this suggestion. We have added a workflow figure as Figure 1—figure supplement 2.

Minor Points:

(1) While the image processing described seems appropriate, I think it is still necessary to include Fourier shell correlation plots for the final structures as supplemental data.

Thank you for pointing out this inadvertent omission. We have added FSC curves in Figure 3—figure supplement 3.

(2) One of the initial workflows used is a Relion 3 pipeline, which is, at this point, quite dated. Is there a reason Relion 4 or 5 was not used instead?

The project started when Relion 3 was the latest version.
